# Cell painting and thermal proteome profiling for inference of drug targets and mechanism of action

**DOI:** 10.1038/s44320-026-00214-9

**Published:** 2026-05-15

**Authors:** Camilla Johansson, Martin Johansson, Phanindra Babu Kasi, Mårten Larsson, Per-Johan Jakobsson, Ulf Göransson, Jordi Carreras Puigvert, Ola Spjuth, Erik T Jansson

**Affiliations:** 1 Department of Pharmaceutical Biosciences, Uppsala University, Uppsala, Sweden; 2 Chemical Biology Consortium Sweden, Science for Life Laboratory, Uppsala, Sweden; 3 Division of Rheumatology, Department of Medicine, Karolinska Institutet and Karolinska University Hospital, Solna, Sweden

**Keywords:** Pharmacology & Drug Discovery

## Abstract

Understanding the mechanism of action (MoA) of bioactive compounds is a central challenge in drug discovery and chemical biology. We propose a strategy for integrating morphological data with proteomics to provide deeper insights into the MoA of compounds. We combine the rich phenotypic profiles of Cell Painting (CP) with the unbiased protein target detection of Thermal Proteome Profiling (TPP), and construct protein–protein interaction networks based on potential targets identified using both assays. We validated our method with public TPP datasets for the five compounds (+)-JQ1, I-BET151, Vemurafenib, Crizotinib and Panobinostat, and public Proteome Integral Solubility Alteration (PISA) data for 49 compounds, together with CP data for 5259 drugs on U2OS cells. We show that the combined approach could accurately identify known MoAs for four out of five validation compounds and known targets for 29 out of 49 compounds. Finally, we deployed our method to characterize sinomenine, a compound with elusive knowledge of MoA. Our findings revealed novel facets of sinomenine's biological activity and highlight the value of multimodal profiling for chemical biology.

## Synopsis

This study integrates cell painting imaging with thermal proteome profiling maps to reveal drug mode of action. The authors validate the method using known drug mode of action and uncover affected pathways for the drug sinomenine, showcasing an integrative pipeline for drug discovery.

- Integration of cell painting and thermal proteome profiling data via protein–protein interaction networks provides precise identification of drug targets.
- Using this method, the authors show that sinomenine has potential anticancer effects and modulates biological pathways associated with rheumatoid arthritis.

## Abstract (web summary)

This study integrates cell painting imaging with thermal proteome profiling maps to reveal drug mode of action. The authors validate the method using known drug mode of action and uncover affected pathways for the drug sinomenine, showcasing an integrative pipeline for drug discovery.

## Graphical abstract

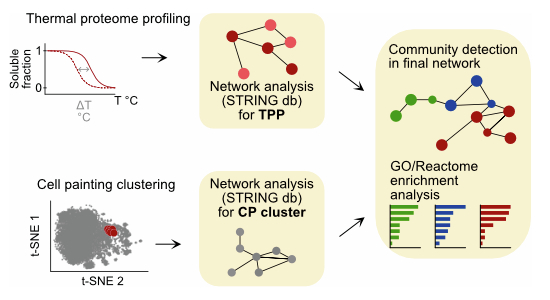

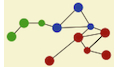

## Introduction

Understanding the complete list of drug targets for a small-molecule compound can help explain adverse toxicity, drug tolerance, and previously unknown mechanisms of action (MoA) that may otherwise cause drugs to fail clinical trials (Lin et al, 2019; Savitski et al, 2014). Studies show that many cancer drugs in clinical trials cause cell death through previously unknown off-target interactions rather than through their intended target (Lin et al, 2019). However, extensive evaluation of the cellular effects of a compound is a complicated and historically expensive undertaking (Schenone et al, 2013). The continuous development of high-throughput omics strategies is currently shifting the drug development workflow from studying single drug–target or drug–phenotype interactions at a time to strategies that assess global drug effects on proteomes, transcriptomes, molecular pathways, or cell morphology, with the advantage of allowing unbiased identification of on- and off-target effects (Schenone et al, 2013).

One milestone has been the development of the cellular thermal shift assay (CETSA) by Martinez Molina et al, 2013, which has allowed researchers to study direct or indirect drug–protein interactions that occur inside intact cells. The method builds on the previous knowledge that small molecules can alter protein melting temperatures during drug–target binding by either stabilizing or destabilizing its target. While this phenomenon had previously been used to confirm drug–target interactions of purified proteins, the invention of CETSA has allowed the study of these interactions in physiological conditions by heating intact cells to different temperatures prior to cell lysis. The fractions of soluble target proteins are then measured at different temperatures or drug concentrations. Shortly after the publication of CETSA, Savitski et al, 2014 demonstrated that the CETSA method could be coupled with mass spectrometry for an unbiased study of protein melting curves on the proteome scale. This method was called Thermal Proteome Profiling (TPP) and has since its conception been used to study drug–target interactions and pathway perturbations for a growing range of compounds (Cigler et al, 2025; Franken et al, 2015; Reinhard et al, 2015; Savitski et al, 2018) in both intact cells and cell extract. Performing TPP on intact cells commonly results in tens to hundreds of proteins with perturbed thermal stability, where the gain or loss of intermolecular interactions due to drug perturbation results in thermal stabilization or destabilization of individual proteins (Cigler et al, 2025; Franken et al, 2015; Reinhard et al, 2015; Savitski et al, 2018). These can be proteins that bind directly to the drug under investigation, but can also be proteins in complex with, or downstream of, the drug target (Savitski et al, 2014). Therefore, additional experiments are often needed to differentiate direct targets from downstream effects.

In the past decade, another high-capacity method has emerged that allows characterizing drug effects based on changes in cell morphology. Cell Painting (CP) is an image-based profiling method in which adherent cells are treated with different drugs or genetically perturbed. The resulting morphological changes are then captured by staining the cells with a fixed set of fluorescent dyes that target different cell compartments. The images of stained cells are processed through software (here we use CellProfiler (Stirling et al, 2021)), where thousands of features are extracted at the single-cell level to describe the morphological perturbation of each treatment. Since CP assays are relatively inexpensive to perform with a high compound multiplexing capacity, especially when combined with automated sample preparation, the method has been used to compare several thousand compounds to each other. Compounds with similar MoAs and drug targets tend to induce similar morphological changes. When there exist well-annotated databases with MoA or targets for a large set of the tested compounds, both supervised and unsupervised machine learning strategies can be employed to make predictions for compounds where this information is missing. Hence, CP has been used for drug discovery, drug repurposing studies, and to predict drug toxicology and efficacy (Seal et al, 2025). Although more and more applications for CP are published every year (Seal et al, 2025), challenges remain when it comes to accurate compound classification. Small-molecule compounds are known to often have many possible targets (Lin et al, 2019), which results in a large set of overlapping MoAs that can differ between cell cycle phases, cell types, and experimental conditions. The accuracy of MoAs and targets predicted through CP will therefore depend on the quality of the available annotated data (Seal et al, 2025).

By integrating CP data with omics strategies, some of these challenges can be tackled. Multiple studies have integrated CP with transcriptomics (Seal et al, 2022), differential expression proteomics (Cigler et al, 2025), chemical structure fingerprints (Sanchez-Fernandez et al, 2023; Seal et al, 2022), and metabolomics (Alijagic et al, 2023); and TPP has been integrated with differential expression proteomics and transcriptomics (Burtscher et al, 2024). Here, we merge information from CP with TPP to combine the power of morphological characterization in CP with the detection of target and pathway perturbation in TPP. To our knowledge, the integration of CP with TPP has only been attempted twice before (Cigler et al, 2025; Wilke et al, 2021), and was limited to comparing putative targets identified with both methods. Our approach focuses on constructing protein–protein interaction (PPI) networks using information from both assays simultaneously to highlight perturbed biological processes. An in-house generated CP dataset that painted 5259 compounds was used in combination with publicly available TPP data for five different compounds: (+)-JQ1, I-BET151, Panobinostat, Vemurafenib, and Crizotinib. We show that our computational pipeline provides enhanced predictability of MoAs compared to either TPP or CP alone. Next, we validated target prediction using our workflow on a 49-compound large public Proteome Integral Solubility Alteration (PISA) dataset. PISA is a high-throughput alternative to TPP, where all different temperature treatments of a sample are pooled prior to TMT-labeling and mass spectrometry analysis (Gaetani et al, 2019). We show that our workflow can successfully integrate PISA data with CP data to improve target prediction for 29 out of 49 compounds. Finally, we conducted a PISA study in-house for the drug sinomenine, a plant-derived alkaloid used in China for the treatment of rheumatoid arthritis and pain, where the MoA is largely unknown (Li et al, 2023) and showcase potential for sinomenine MoAs. The strategy has been summarized into a workflow with code written in Python and is available on GitHub (https://github.com/camilla-johansson/integrate-cp-tpp).

## Results

**Fig. 1:**
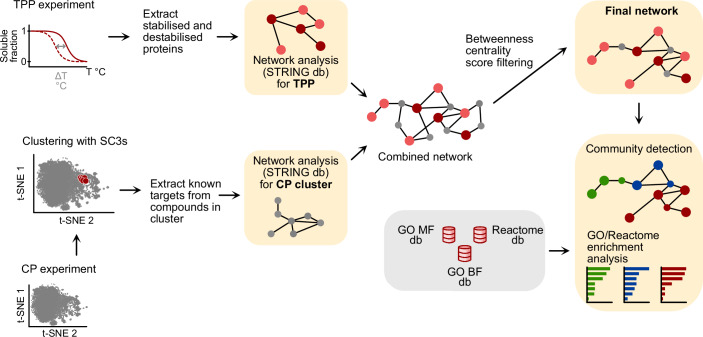
Schematic overview of our computational pipeline. Thermal Proteome Profiling (TPP) experimental data for the compound of interest are processed and analyzed to retrieve a list of significantly stabilized and destabilized proteins. The list of perturbed proteins is then submitted to string.db (Szklarczyk et al, 2023) through their API to retrieve a protein–protein interaction (PPI) network of physically associated proteins. In parallel, processed Cell Painting (CP) data is clustered using Single Cell Consensus Clustering with speed (SC3s) (Quah and Hemberg, 2022) to identify groups of compounds with similar morphological changes as the compound of interest. Next, from the identified cluster, target information is extracted from similar compounds and submitted to string.db to construct another physical PPI network. The proteins identified in TPP and CP cluster are combined into a third PPI network. Betweenness centrality scores (BCSs) are calculated for the combined network and used to filter the CP-derived nodes. Only CP-derived nodes with high BCS, which also contribute to maximizing the number of connected TPP nodes, are kept in the final PPI network. Annotations on subcellular location and cell line-specific protein expression levels are retrieved from the Human Protein Atlas (HPA) and merged with the network. Finally, the network is subdivided into communities using the Clauset–Newman–Moore greedy modularity maximization. Gene Ontology (GO) and Reactome enrichment analysis are performed for each community to detect possible mechanisms of action (MoA) for the compound of interest. The top *n* nodes (with n typically set to 10) with the highest BCS in the final network represent possible drug targets.

Thermal proteome profiling (TPP) on whole cells has been used repeatedly to infer protein–protein interactions (PPI) and protein complexes (Schenone et al, 2013). Figure 1 shows our strategy that combines information from TPP and CP to generate PPI networks. We reasoned that combining information from TPP with curated PPI databases could allow the detection of perturbed biological pathways while allowing for missed interactions. Furthermore, we hypothesized that missing proteins can be added from cell painting (CP) data through similarity to other compounds with known targets or MoAs, thereby improving the PPI networks. We have herein developed a data-driven workflow for integrating CP and TPP data using a PPI network approach.

Our workflow begins with the complete list of stabilized or destabilized proteins from TPP (or PISA) data as input to generate a physical STRING PPI network. Next, the STRING database collects and summarizes data from different sources to create networks of high-confidence protein associations. The physical STRING networks, used in our model, contain interactions based on scored experimental data (IntAct, BioGRID, DIP, PDB, etc.); interactions from databases (KEGG, Reactome, GO complexes, MetaCyc, EBI complex portal); and text mining (Szklarczyk et al, 2023). In turn, the resulting network can be combined with subcellular expression data or cell line-specific mRNA-seq data from the Human Protein Atlas (www.proteinatlas.org) (Thul et al, 2017; Zhang et al, 2023), as well as Gene Ontology (GO) (Ashburner et al, 2000; The Gene Ontology Consortium, 2026) and Reactome annotations (Milacic et al, 2024), to investigate perturbed cellular processes. Finally, we calculated betweenness centrality scores (BCSs) (Freeman, 1977) to propose possible targets. It has been shown that drug targets tend to have higher BCS in STRING networks compared to proteins that are not targets of known drugs (Moya-García et al, 2017), possibly due to their central role in key biological processes. Furthermore, targets and off-targets associated with side effects generally have a higher BCS than targets associated with the desired effect. Hence, we reasoned that the targets and potential off-targets of a drug would most likely be found among the nodes with the highest BCSs, given that these proteins are present within the PPI network.

We performed unsupervised clustering on the entire CP dataset to supplement the TPP network with target information from a CP dataset based on 5259 chemical compounds from the SPECS drug repurposing repository. A consensus clustering strategy was applied based on *k*-means clustering on dimensionally reduced features. Compounds found within the same cluster as the compound in the TPP assay would trigger similar morphological changes. We hypothesized that some, if not all, of these compounds would act on the same target as our investigated drug or alternatively act on targets that are found within the same biological pathways. Hence, these targets could be added to the TPP PPI network to “fill in the gaps” for perturbed proteins that are expected to be engaged but were missing in the dataset. Through a two-step PPI network construction, where the BCSs of all cluster-derived nodes were calculated in the first step, we filtered the final PPI network to only contain CP cluster-derived nodes, which increased the number of connected TPP-derived nodes.

### Development of computational pipeline using compounds (+)-JQ1 and I-BET151

In order to develop, optimize, and finally test the performance of the proposed workflow in Fig. 1, we used the publicly available (+)-JQ1 and I-BET151 2D-TPP datasets (Savitski et al, 2018). (+)-JQ1 and I-BET151 are both BET bromodomain protein inhibitors with shared targets (Savitski et al, 2018). Both compounds display distinct and overlapping morphological effects in cell painting data (mean grit scores of 4.19 over two biological replicates, for both compounds, corresponding to roughly a total morphological change of four standard deviations from untreated negative control cells) and are well characterized in the literature with several known targets and MoAs (Filippakopoulos et al, 2010; Shorstova et al, 2021). In addition, TPP data for (+)-JQ1 were available for both whole cells and cell extracts.

A list of known targets and off-targets for (+)-JQ1 and I-BET151 was constructed from the literature to allow quantification of the specificity and sensitivity of the proposed pipeline (Table 1). (+)-JQ1 targets BRD4 and BRDT, with documented off-target effects on BRD2, BRD3 and HADHA. I-BET151 targets BRD4, BRD3 and BRD2.

**Table 1:** Model performance on publicly available data for five tested compounds

Compound	Known target(s)	Off-target(s)	Cluster with target(s) in CP	TPP data includes known target(s)	Grit score in CP data (rep 1 and 2)	Model predicts target(s)	Model predicts MoA(s)
(+)-JQ1	BRD4, BRDT (Filippakopoulos et al, 2010)	BRD2, BRD3, HADHA, SRRM2 (Filippakopoulos et al, 2010; Trowbridge et al, 2022)	Yes	Yes	4.24 and 4.14	Yes	Yes
I-BET151	BRD2, BRD3, BRD4 (Savitski et al, 2018)		Yes	Yes	3.97 and 4.40	Yes	Yes
Vemurafenib	BRAF^V600E^, RAF1 (Brummer and McInnes, 2020)	FECH (Savitski et al, 2014)	Yes	Yes	2.61 and 2.71	Yes	Yes
Crizotinib	ALK, MET, ROS1 (Musa et al, 2024)	ABL1, BCR, JAK2, ABCB1 (Musa et al, 2024)	No	No^a^	4.40 and 4.49	No^b^	Possibly
Panobinostat	HDAC1, HDAC2, HDAC3, HDAC4, HDAC5, HDAC6, HDAC7, HDAC8, HDAC9, HDAC10, HDAC11 (Atadja, 2009)		Yes	Yes	3.18 and 2.42	Yes	Yes

When running the models, the known targets for the compound of interest were censored from the compound database to simulate that the protein targets were unknown.

^a^Targets are membrane proteins, which are not generally detectable with the TPP method used in the original article.

^b^Model predicts proteins which bind directly to targets.

There are several different protocols for performing TPP, e.g., temperature regression (TPP-TR), concentration regression (TPP-CR), 2D-TPP, isothermal dose-response (ITDR), and Proteome Integral Solubility Alteration (PISA). The experiments can be designed to either be labeled with isobaric tandem mass tags (TMT), or label-free. In addition, there are several computational pipelines to obtain thermal shift, e.g., fitting sigmoidal melt curves or comparing the Area Under Curve (AUC). To accommodate these variations in experimental design, we have developed an analysis workflow that is independent of the TPP protocol used. Hence, the only required input is a list of proteins deemed to be either stabilized or destabilized by the chosen TPP strategy.

**Fig. 2:**
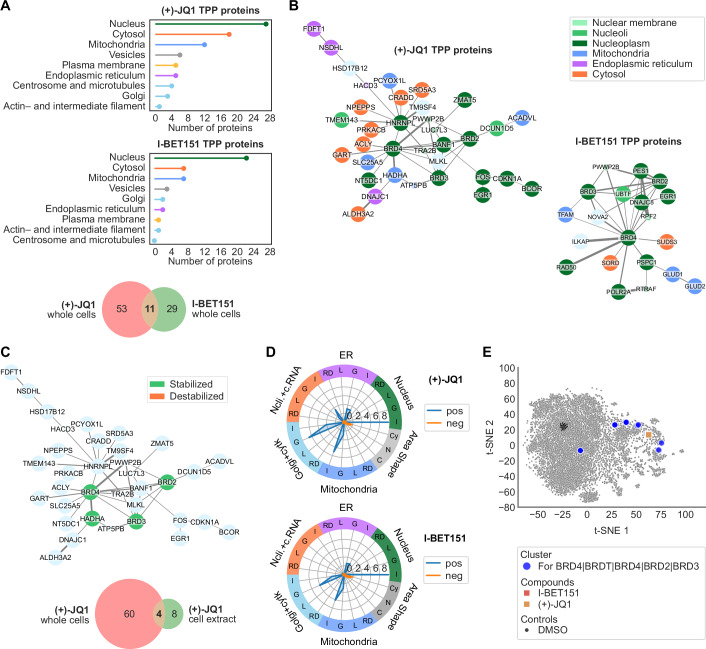
Thermal proteome profiling (TPP) and cell painting analysis of JQ1 or I-BET151-treated cells. (**A**) Lollipop chart for subcellular location of the 67 versus 40 proteins found to be either stabilized or destabilized in TPP for compounds (+)-JQ1 (top) and I-BET151 (middle), respectively. Subcellular location of each protein, based on immunohistochemistry and confocal microscopy, was retrieved from the Human Protein Atlas (proteinatlas.org). When a protein had more than one subcellular location assigned, that protein was included once per subcellular location in the graph. The bottom graph shows a Venn diagram of the overlap between proteins stabilized/destabilized in (+)-JQ1 and I-BET151. Only 11 proteins were found to overlap between the two compounds. (**B)** Physical protein–protein interaction (PPI) networks for the proteins found to be either stabilized or destabilized in TPP for compounds (+)-JQ1 and I-BET151, respectively. PPI networks were retrieved from the STRING db. Nodes are colored by subcellular location (from Human Protein Atlas). The sizes of the nodes indicate whether the proteins have been found in TPP (large size) or CP (small size). Edge thickness corresponds to the confidence score of the interaction. (**C**) Top: Physical PPI networks for (+)-JQ1 (same as (**B**)), except green nodes here indicate proteins found to be stabilized by (+)-JQ1 in cell extract. Bottom: Venn diagram for overlap of proteins detected in whole cells versus cell extract after perturbation with (+)-JQ1. (**D**) Radar chart showing mean z-scores for CellProfiler features in cell painting data for (+)-JQ1 and I-BET151. The blue and orange lines indicate positive or negative mean z-score values, respectively. Note that there is no intrinsic order, and the shading is only providing visual clarity of the features. Features were grouped into categories based on two criteria: (i) Cell Profiler module, i.e., Intensity (I), Granularity (G), Location (L) and RadialDistribution (RD); and (ii) stains, i.e., Nucleus (Hoechst), ER (Concanavalin A), Nucleoli and cytoplasmic RNA (SYTO14), Golgi apparatus and F-actin cytoskeleton (WGA and Phalloidin) and Mitochondria (Mitotracker). Features were only considered for the object Cell, except for features from the stain for Nucleus, which were only considered in the object Nucleus. In addition, area-shape related features were grouped by cell compartment, i.e., Cell (C), Cytoplasm (Cy), and Nucleus (N). (**E**) t-SNE for the morphological features in the SPECS cell painting data on U2OS cells. Blue dots show the location of all cells treated with BET bromodomain inhibitors sharing at least one target with either (+)-JQ1 or I-BET151. Despite several BET bromodomain inhibitors clustering close to (+)-JQ1 or I-BET151, there are also several compounds with distinctly different morphological changes. Source data are available online for this figure.

THP-1 whole cells had been treated with (+)-JQ1 or I-BET151 in DMSO at concentrations of 20.0, 5.0, 1.0, and 0.1 μM, or DMSO vehicle control, and subsequently heated to 12 different temperatures in duplicates, followed by cell lysis and TMT-DDA analysis as described elsewhere (Ray et al, 2019). In total, 67 proteins displayed dose-dependent changes in thermal stability upon treatment with (+)-JQ1 for at least two consecutive temperatures (Appendix Table S1). For I-BET151 treatment, the corresponding number of affected proteins was 40 (Appendix Table S2). For both compounds, the affected proteins are predominantly expressed in the nucleus, cytosol, and mitochondria (Fig. 2A).

Protein–protein interaction networks were constructed for the 67 versus 40 affected proteins, respectively (Fig. 2B). The resulting networks connected 30 proteins for (+)-JQ1 and 17 proteins for I-BET151 (Appendix Tables S3–S4). BRD4, BRD3, and BRD2 were identified in both networks with BCSs of 0.48, 0.05, and 0.13, respectively, for (+)-JQ1 and 0.77, 0.02, and 0.03, respectively, for I-BET151. Additionally, HADHA was identified in the (+)-JQ1 network with a BCS of 0.12. While the two compounds are known to act on similar targets (BRD4, BRD3, and BRD2), there were only 11 affected proteins shared between the two compounds (Fig. 2A), and the PPI networks looked quite different (Fig. 2B). This suggests that (+)-JQ1 and I-BET151 regulate different pathways downstream of target binding.

When performing TPP on intact cells (i.e., heating the cells before cell lysis), the spatial relationship between proteins is preserved during heating, which makes it possible to detect stabilization or destabilization events of proteins that are not directly binding to the compound or target protein. Lysing cells prior to heat treatment reduces the biologically relevant spatial distance between proteins. Hence, stabilized or destabilized proteins detected from TPP conducted on lysed cells are likely to directly bind the compound. Since the dataset for (+)-JQ1 contained TPP data generated on both whole cells and cell extract, we looked at the overlap between these two. Of the 12 proteins perturbed by (+)-JQ1 in cell extract, only 4 overlapped with proteins detected in TPP on whole cells (Fig. 2C; Appendix Table S5).

Although running TPP on whole cells and cell extract appears to be a promising strategy for identifying direct targets and MoA for a compound, these are time-consuming experiments, and researchers often opt to run TPP on whole cells only. Furthermore, TPP has a tendency to miss some perturbed proteins, which could in turn complicate the assessment of perturbed pathways. One reason for proteins being missed can be instrument-specific, e.g., a peptide not being detected due to peptide ionization or instrument-specific lower limit of detection. Other causes for missingness can be attributed to the experimental design of TPP. Proteins with higher thermal stability than the tested thermal range in TPP are likely to be wrongly considered as unaffected (Schenone et al, 2013). Furthermore, membrane-associated proteins can sediment with the cell membrane when centrifuged (Reinhard et al, 2015).

We therefore sought to investigate if combining the information from CP experiments with whole-cell TPP could lead to similar conclusions or even contribute to adding important nodes which were missing from TPP. (+)-JQ1 and I-BET151 displayed highly similar morphological changes in CP (Fig. 2D,E) with the largest changes observed in the nucleus, mitochondria, and Golgi apparatus/cytoskeleton. While the morphological patterns were very similar, overall larger effect was observed in (+)-JQ1 treated cells compared to I-BET151 (Fig. 2D). Further, we also observed that while both compounds showed morphological perturbation nearly identical to other bromodomain inhibitors, not all bromodomain inhibitors in the CP data clustered together (Fig. 2E). We hypothesized that by supplementing the PPI network from TPP with known target information from compounds clustering close to the compound(s) of interest in morphological space, we would get a more comprehensive picture of target binding and MoA.

**Fig. EV1:**
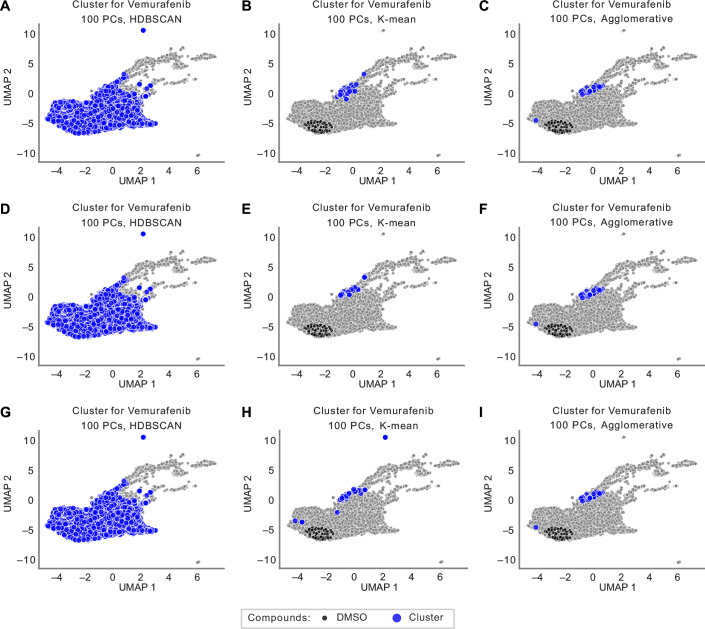
Comparison of clustering algorithms for CP data on the compound Vemurafenib. UMAP representations of clustering Vemurafenib with (**A**, **D**, **G**) HDBSCAN clustering; (**B**, **E**, **H**) *k*-means clustering; (**C**, **F**, **I**) agglomerative clustering. Each panel for HDBSCAN and *k*-means clustering was generated with random number seeds, while agglomerative clustering is deterministic which is showed with three identical panels. HDBSCAN was unable to cluster Vemurafenib while both *k*-means and agglomerative clustering generated clusters of  ~20 compounds.

We considered that a suitable clustering algorithm for our purpose should be able to detect small (about 20–60 samples), tight clusters in CP data with high reproducibility. Preliminary clustering attempts for the CP data using *k*-means, HDBSCAN, and agglomerative clustering showed that the tightest clusters could be achieved with using *k*-means and agglomerative clustering with a prior principal component analysis (PCA) feature reduction. In contrast, HDBSCAN was unable to cluster the drugs at all. Figure EV1 shows a comparison of how the three clustering algorithms behaved for Vemurafenib. We also show similar behavior for I-BET151 and (+)-JQ1 (Appendix Figs. S1 and S2). *K*-means clustering on PCA data with between 100 and 300 *k* clusters performed similarly to agglomerative clustering using the same number of clusters as input parameters.

Consensus clustering can be used to overcome the reproducibility issues of stochastic clustering algorithms by repeatedly cluster the same data a large number of times with different starting parameters and then combine the resulting clusters into a consensus matrix (Quah and Hemberg, 2022). We used the consensus clustering algorithm SC3s (Kiselev et al, 2017; Quah and Hemberg, 2022), which combines PCA feature reduction with *k*-means clustering and finally summarizes the consensus matrix using complete-linkage hierarchical clustering. We ran the algorithm with 2000 runs across five different numbers of *k*-clusters, between 100 and 180. Finally, for each compound of interest, we selected the value of *k* that resulted in the smallest consensus cluster. As a lower cutoff limit, the selected cluster needed to contain at least 20 samples. To check for reproducibility, the whole strategy was repeated 10 times. We could show that even though the calculated clusters had silhouette scores around 0 (Appendix Fig. S3), which indicate poor cluster separation, the consensus clustering strategy still resulted in highly reproducible clusters for all tested compounds except Vemurafenib (Appendix Tables S6–S11).

**Fig. 3:**
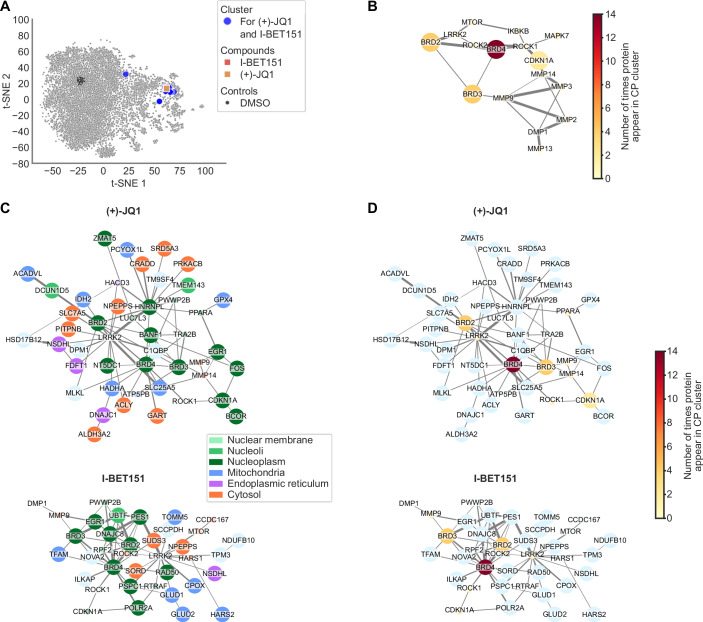
Using data-driven cluster analysis to supplement the TPP PPI network with information from similar compounds in cell painting data improves target prediction. (**A**) t-SNE for CP SPECS data showing (+)-JQ1 (yellow), I-BET151 (red), and a cluster of similar compounds (blue) identified using the consensus clustering algorithm SC3s on 13 principal components. *k* for the *k*-mean clustering algorithm was varied between 100 and 180. (**B**) Physical PPI network for known protein targets of the compounds identified in the cluster in (**A**). (+)-JQ1 and I-BET151 were blinded from the compound list before retrieving known targets. The network is colored by the number of times a protein target is present within the cluster. The sizes of the nodes indicate whether the proteins have been found in TPP (large size) or CP (small size). Edge thickness corresponds to the confidence score of the interaction. (**C**, **D**) Physical PPI network combining proteins identified in TPP and CP cluster, after applying a betweenness centrality calculation on the nodes from CP clustering. The number of CP-supplied nodes to keep was chosen as the minimum number of nodes needed to maximize the connection between the TPP-identified proteins. Top: (+)-JQ1, bottom: I-BET151. The nodes are colored by (**C**) subcellular location and (**D**) number of times a protein target is present within the cluster in (**A**). Source data are available online for this figure.

**Fig. EV2:**
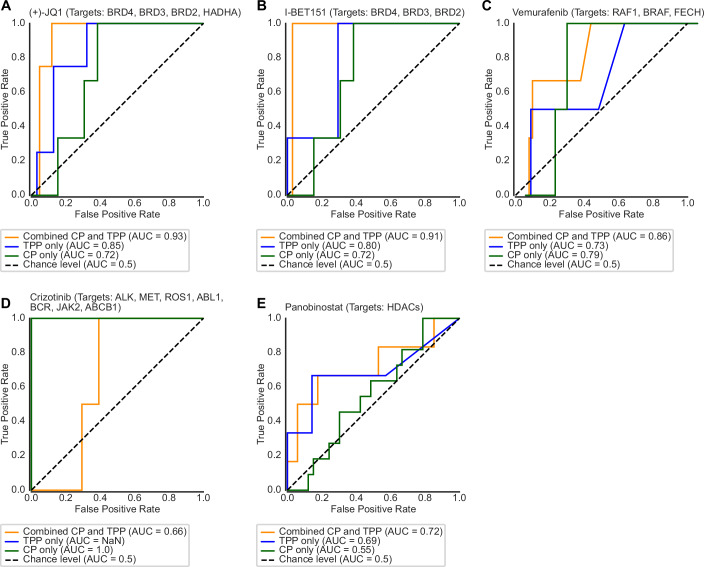
Combining cell painting (CP) and thermal protein profiling (TPP) generally improves target identification using betweenness centrality scores. Receiver operating characteristic curves were constructed from PPI networks using betweenness centrality scores as ranks. (**A**) (+)-JQ1 (known targets: BRD4, BRD3, BRD2 and HADHA), (**B**) I-BET151 (known targets: BRD4, BRD3 and BRD2), (**C**) Vemurafenib (known targets: RAF1, BRAF and FECH), (**D**) Crizotinib (known targets: ALK, MET, ROS1, ABL1, BCR, JAK2 and ABCB1), (**E**) Panobinostat (known targets: HDACs).

The smallest consensus cluster containing both (+)-JQ1 and I-BET151 (Fig. 3A) comprised a total of 21 compounds. Only 9 of these were bromodomain inhibitors (Appendix Fig. S4). After blinding the known information from (+)-JQ1 and I-BET151, we constructed a physical PPI network from the known target information of the clustered compounds (Fig. 3B). BCSs were calculated for CP cluster proteins (Appendix Table S12), and we noted that BRD4, BRD2, and BRD3 could be identified in the network from CP data alone. Hence, in this case, both CP and TPP had the potential to identify three to four of the known targets/off-targets of (+)-JQ1 and I-BET151. The targets identified in the cluster were then combined with the affected proteins from TPP data, and a new PPI network was constructed. Since this network became very large, the betweenness centrality scores for the CP cluster targets were recalculated and sorted. The proteins with the lowest BCS from the CP cluster were iteratively removed as long as the total number of TPP nodes included in the network was not reduced. For (+)-JQ1, six nodes from the CP cluster were kept (LRRK2, MMP14, MMP9, ROCK1, PPARA, and LUC7L3). For I-BET151, eight nodes fulfilled the same criteria (LRRK2, MMP9, ROCK1, MTOR, ROCK2, CDKN1A, DMP1 and HARS1). Figure 3C,D shows PPI networks for all identified TPP nodes combined with the six ([+]-JQ1) versus eight (I-BET151) selected CP nodes. We observed that this strategy overall slightly improved the ranking of BCSs for known BRD4, BRD2, BRD3, and HADHA for (+)-JQ1 (Table 2), and BRD2 and BRD3 for I-BET151 (Appendix Table S13), which was further assessed using ROC curve analysis (Fig. EV2).

**Table 2:** Top ten proteins with the highest betweenness centrality score (BCS) for (+)-JQ1

Uniprot ID	Gene name	Protein name	BCS	BCS rank change from TPP
Q5S007^c^	LRRK2	Leucine-rich repeat serine/threonine-protein kinase 2	0.41	
P14866	HNRNPL	Heterogeneous nuclear ribonucleoprotein L	0.37	–1
O60885^a^	BRD4	Bromodomain-containing protein 4	0.25	–1
P25440^b^	BRD2	Bromodomain-containing protein 2	0.13	+2
P40939^b^	HADHA	Trifunctional enzyme subunit alpha, mitochondrial	0.11	+2
Q07869^c^	PPARA	Peroxisome proliferator-activated receptor alpha	0.07	
O75531	BANF1	Barrier-to-autointegration factor	0.06	–5
O95232	LUC7L3	Luc7-like protein 3	0.06	+1
Q15059^b^	BRD3	Bromodomain-containing protein 3	0.06	+5
P50281^c^	MMP14	Matrix metalloproteinase-14	0.05	

^a^Known target for (+)-JQ1.

^b^Known off-target for (+)-JQ1.

^c^Not present in TPP dataset, added from computational pipeline.

**Fig. 4:**
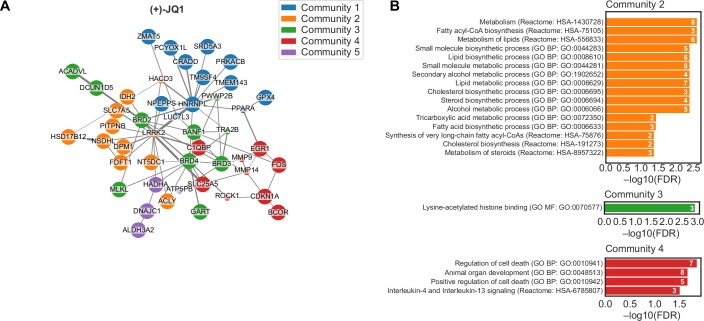
Gene Ontology (GO) and Reactome enrichment analysis of (+)-JQ1 PPI network graph. (**A**) Physical PPI network (same as in Fig. 3C) labeled by community. Communities were identified using Clauset–Newman–Moore greedy modularity maximization. The sizes of the nodes indicate whether the proteins have been found in TPP (large size) or CP (small size). Edge thickness corresponds to the confidence score of the interaction (**B**). GO Molecular function (GO MF), GO Biological Process (GO BP), and Reactome pathway enrichment analysis for each community in (**A**). Only enrichment with FDR ≤0.05 are shown. Source data are available online for this figure.

**Fig. EV3:**
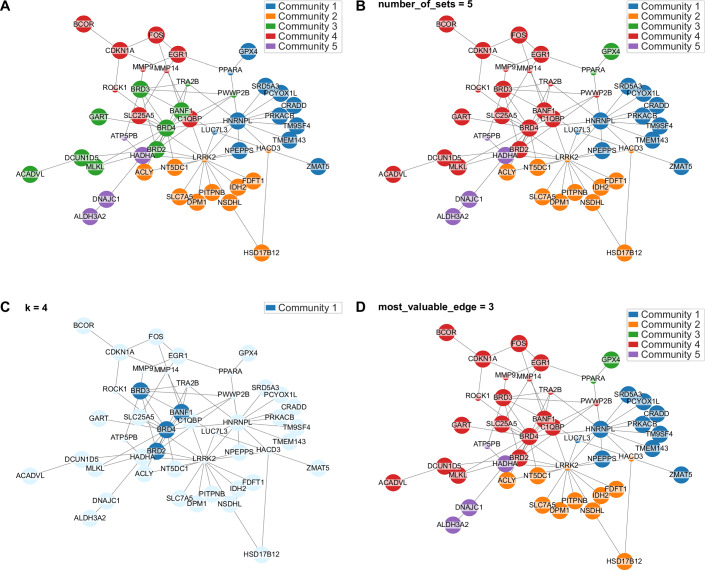
Evaluation of four different community detection algorithms on the final PPI networks of (+)-JQ1. (**A**) Clauset–Newman–Moore greedy modularity maximization. (**B**) Girvan–Newman algorithm with number_of_sets set to 5. (**C**) *k*-clique communities algorithm with *k* set to 4. (**D**) Edge betweenness partition with most_valuable_edge set to 3. All four algorithms were executed using the NetworkX Python package.

The final PPI network in Fig. 3C,D was further classified into five communities using the Clauset–Newman–﻿Moore greedy modularity maximization (Clauset et al, 2004) (Fig. 4A). We additionally evaluated the Girvan–Newman, edge betweenness partition, and *k*-clique communities algorithm, which showed that either very small communities were generated (Girvan–Newman and edge betweenness partition), or nodes were not associated with a community at all (*k*-clique communities). Hence, we finally chose the Clauset–Newman–Moore greedy modularity maximization algorithm that did not require a prior assumption of the number of, or size of, generated communities. This algorithm was able to generate communities with at least two members and did not fail to associate any nodes with a community. Figure EV3 shows a comparison of how these evaluated algorithms perform. Gene Ontology (GO) and Reactome enrichment analysis (Fig. 4B) suggested that (+)-JQ1 possibly acts through mechanisms like lysine-acetylated histone binding (Community 3), activation of cell death (Community 4) or affecting lipid metabolism (Community 2). This was in line with the known MoA for (+)-JQ1 (Filippakopoulos et al, 2010; Shorstova et al, 2021). In contrast, GO and Reactome enrichment analysis for I-BET151 highlighted transferase activity, kinase activity, mitotic cell cycle processes (Community 3), and oxidoreductase activity (Community 1) (Appendix Fig. S5).

### Applying workflow to vemurafenib, crizotinib, and panobinostat allowed rediscovery of known targets and mechanisms of action

Our computational pipeline was applied to publicly available datasets for three more compounds: Vemurafenib (Savitski et al, 2014), Crizotinib (Savitski et al, 2014), and Panobinostat (Franken et al, 2015). For all three of these compounds, TPP data were only available for K-562 whole cells, and the TPP experiment had been run as ITDR-TPP or TPP-TR. Clustering reproducibility was high for Crizotinib (Appendix Table S10) and Panobinostat (Appendix Table S11), but had a low reproducibility for Vemurafenib (Appendix Table S9). This could possibly be explained by the comparably lower grit score for Vemurafenib (Table 1). Despite this, integrating TPP data with CP cluster data for Vemurafenib slightly improved target identification over using TPP or CP alone (Fig. EV2). Notably, the known target BRAF was missing from the TPP data (Appendix Tables S14–S15) while the off-target FECH was missing from the CP cluster (Appendix Table S16). This further highlights the benefit of combining CP with TPP for improving the chances of target identification (Appendix Fig. S6; Table S17). Furthermore, GO and Reactome analysis indicate an involvement of the MAPK pathway as well as RAF1 and BRAF pathways (Appendix Fig. S5A, Community 1). In contrast, if one only considers annotated MoA for the CP cluster, there is no clear enrichment for one MoA over another (Appendix Fig. S7).

For Panobinostat, TPP alone detected three out of eleven known targets (HDAC6, HDAC8, and HDAC10, Appendix Tables S18–S19). CP cluster analysis added another eight known targets (HDAC1, HDAC2, HDAC3, HDAC4, HDAC5, HDAC7, HDAC9, and HDAC11, Appendix Fig. S8 and Appendix Table S20). While CP cluster data contained the highest incidence of known targets (Appendix Table S20), the targets were not necessarily found among the proteins with highest BCSs, which gave a poor target prediction when using CP data alone (AUC = 0.55, Fig. EV2). Combining TPP and CP resulted in six targets being identified (HDAC1, HDAC3, HDAC5, HDAC6, HDAC8, and HDAC10), with four of these being found among the top ten highest BCSs (Appendix Table S21). GO enrichment analysis suggested histone deacetylase activity as an affected pathway (Appendix Fig. S9, Community 1) along with a large set of affected signal transductions and dysregulation of metabolic processes. Several components of the 26S proteasome (PSMC1, PSMC4, PSMC5, PSMD1, PSMD8, PSMD11 and PSMD13) were found to be destabilized (Appendix Tables S18 and S21).

Crizotinib (or more specifically, the (R)-Crizotinib stereoisomer) is FDA approved for c-ros oncogene 1 (ROS1) and anaplastic lymphoma kinase (ALK) inhibition of ROS1-positive or ALK-positive non-small cell lung cancer (NSCLC) (Schenone et al, 2013). The compound is also known to inhibit c-Met/hepatocyte growth factor receptor (MET), which is overexpressed in many cancers (Schenone et al, 2013). These three proteins are all transmembrane proteins. Classical protocols for TPP do not include any detergent and, therefore, have a low likelihood of detecting transmembrane proteins (Reinhard et al, 2015). For this reason, it was not surprising that none of ROS1, ALK, or MET were found as affected proteins in the TPP data for Crizotinib (Table 1 and Appendix Tables S22–23). In CP, compounds acting on ALK, ROS1, or MET displayed highly heterogeneous morphological changes, with no clear enrichment of ALK/MET/ROS1 inhibitors around Crizotinib (Appendix Fig. S10). Instead, two known off-target proteins, ABL1 and BCR, were identified in the cluster (Appendix Table S24). Combining CP and TPP data suggested that Crizotinib possibly acts as a Bcr-Abl kinase inhibitor (Appendix Fig. S11), with a MoA involving cadherin binding (Appendix Fig. S11B, Community 2) or actin cytoskeleton organization (Appendix Fig. S11B, Community 4). None of the known targets of Crizotinib were found among the 10 proteins with the highest BCS (Appendix Table S25) and combining CP with TPP here resulted in reduced target prediction over using CP alone (Fig. EV2). By comparing mRNA expression in U2OS cells (used in the CP assay) and K-562 cells (used in TPP) for known targets and off-targets of Crizotinib (Musa et al, 2024), we found that none of the cell lines expressed ALK or ROS1, and with only U2OS cells expressing MET (Appendix Fig. S12). Instead, the off-target ABL1 was expressed in both cell lines. This suggests that the perturbed proteins in TPP may be caused by off-target pathways rather than through inhibition of ALK, ROS1, or MET.

The high expression of ABL and BCR in K-562 cells (neither of which was detected in the TPP data) along with stabilization of GRB2 suggests that the observed effects in TPP could be caused by Crizotinib interacting with the Bcr-Abl pathway. On the contrary, the morphological changes observed in U2OS cells in CP could be a composite effect of both MET and ABL1 inhibition. For (+)-JQ1, I-BET151, Panobinostat, and Vemurafenib, known targets and off-targets were similarly expressed in both the cell line used in TPP and CP (Appendix Fig. S12). Hence, our analysis model correctly identified the known targets and MoAs of four out of five compounds (Table 1). For the fifth compound, Crizotinib, the model suggested a possible MoA through an off-target interaction.

### Validation of target prediction using 49 compounds

Further, we sought to systematically validate how well our computational pipeline predicted targets in a large public PISA dataset by (Van Vranken et al, 2024), where K-562 cells had been treated with 96 compounds or DMSO negative controls across 32 experiments. For the sake of validating our workflow, the target annotations stated for each compound in the publication were assumed to be the complete set of known true targets. Of the 96 compounds assayed in the PISA data, 64 compounds were also present in our 5259 compound CP data. We selected 49 of these compounds with a grit score above two in the CP data (Appendix Fig. S13A), to ensure that the selected compounds caused a quantifiable morphological change. We re-analyzed the PISA data using the limma R-package to identify significant thermal shifts compared to DMSO controls while simultaneously accounting for batch effects (Appendix Fig. S13B). Compared to the empirically derived strategy for identifying thermal shifts that was employed by Van Vranken et al, 2024, limma generated more significantly affected proteins (adjusted *P* value  < 0.01) per compound. We observed that increasing the number of significant thermal shifts from a median of 21 to 73.5 had a positive effect on the performance of our workflow (Appendix Fig. S13C,D). Next, we analyzed the complete set of 49 compounds from the PISA data with our workflow (Appendix Fig. S13E,F). Known targets were included in the final network for 32 compounds. Of these, our model accurately predicted targets with a ROC AUC ≥0.7 for 21 compounds. Using PISA or CP data alone predicted targets with a ROC AUC ≥0.7 for 15 and 14 compounds, respectively. Of the 32 compounds in which true targets were included in the final network, the combined model outperformed PISA alone in 22 cases and CP alone in 18 cases. For 9 of the 49 compounds tested, target prediction could not be achieved with either of the three models since the true targets were neither included in the PISA data nor the CP cluster. Hence, we show that combining PISA and CP data using our proposed strategy improves target prediction. For all individual ROC curves, see Appendix Figs. S14–S17.

### Characterization of sinomenine MoA

**Fig. 5:**
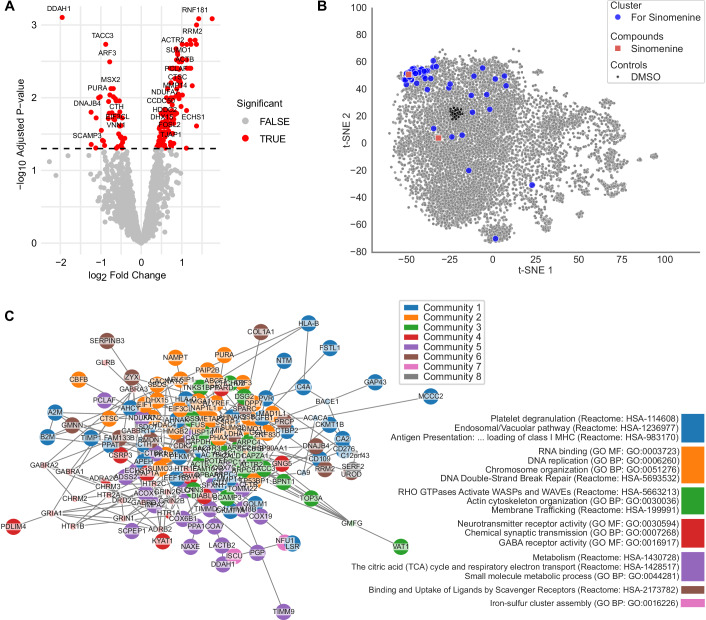
Sinomenine affects protein–protein interactions of molecular pathways related to neurotransmitter signaling, metabolism, and cellular structure. (**A**) Volcano plot of thermal proteome profiling data (*n* = 3 biological replicates), where negative values of log_2_-fold changes indicate a destabilization of the protein and positive values a stabilization, respectively. P values were calculated using the R-package limma and adjusted for multiple hypothesis testing using the Benjamini-Hochberg method. (**B**) t-SNE plot of cell painting data clustered using the unsupervised clustering Python package SC3. Blue highlights the cluster that includes sinomenine. (**C**) Protein–protein interaction network using our workflow for a combination of thermal proteome profiling and cell painting data. The colors indicate communities identified using Clauset–Newman–Moore greedy modularity maximization. The node sizes indicate whether the proteins have been found in TPP (large nodes) or CP (small nodes). Edge thickness corresponds to the confidence score of the interaction. Selected enriched pathways for each community are shown in the annotation panel on the right side. Source data are available online for this figure.

Next, we sought to use our computational pipeline to identify potential MoAs of sinomenine on U2OS cells. We generated PISA data for sinomenine, where cells were treated for 1 h with 10 and 30 μM sinomenine, respectively, resulting in 175 and 121 significant thermal shifts (Dataset EV1). There were 94 thermally affected proteins that were common between both treatments. We combined the stabilized and destabilized proteins from the PISA experiment (Fig. 5A) with CP cluster data (Fig. 5B) for construction of a PPI network with our developed analysis method (Fig. 5C). Table 3 shows the top 10 BCS for the PPI networks for each concentration of sinomenine, highlighting several plasma membrane receptors: *β*-2 adrenergic receptor (ADRB2) and Glutamate Ionotropic Receptor NMDA Type Subunit 2C (GRIN2C), which strongly support that sinomenine acts on receptors involved in the nervous system. U2OS cells express these two membrane protein receptors endogenously. GO and Reactome enrichment analysis following community partitioning on PPI network showed enrichment for terms like “Neurotransmitter receptor activity”, “Chemical synaptic transmission”, and “GABA receptor activity” (Community 4, Fig. 5C), which supports an involvement of the nervous system. Previous studies in mice have shown that sinomenine causes overexpression of *γ*-aminobutyric acid (GABA) (Yoo et al, 2017). Intriguingly, treatment with sinomenine showed Cyclin-dependent kinase 2, (CDK2) among the top BCS, and enrichment analysis (Fig. 5C) revealed that sinomenine impacts pathways related to RNA binding, DNA replication, Chromosome organization, and DNA Double-Strand Repair (Community 2, Fig. 5C). These pathways point to possible anti-tumor effects, corroborating findings in previous studies of sinomenine (Wang et al, 2025; Zhu et al, 2023). Further, we found effects on pathways related to cellular structure and transport, such as Actin cytoskeleton organization, Membrane trafficking, and RHO GTPases Activate WASPs and WAVEs (Community 3, Fig. 5C). This was further corroborated by the high BCS of Actin beta (ACTB). Finally, high BCS for Sideroflexin-1 (SFXN1) was supported by another subcluster of the network that showed effects on metabolism, including the terms Small-molecule metabolic process, the citric acid (TCA) cycle, and respiratory electron transport (Community 5, Fig. 5C). Table 4 shows the clinical impact of the modulation of the proteins and biological pathways affected by sinomenine.

**Table 3:** Top ten proteins with the highest betweenness centrality score (BCS) for sinomenine

Concentration	Uniprot ID	Gene name	Protein name	BCS
10 μM	P60709	ACTB	Actin, cytoplasmic 1	0.380146
	P54578	USP14	Ubiquitin carboxyl-terminal hydrolase 14	0.126951
	P61956	SUMO2	Small ubiquitin-related modifier 2	0.120388
	Q9H9B4	SFXN1	Sideroflexin-1	0.111881
	P24941	CDK2	Cyclin-dependent kinase 2	0.078925
	P07900*	HSP90AA1	Heat shock protein HSP 90-*α*	0.056136
	Q04760	GLO1	Lactoylglutathione lyase	0.048132
	P26583	HMGB2	High mobility group protein B2	0.047893
	P04406	GAPDH	Glyceraldehyde-3-phosphate dehydrogenase	0.046145
	Q13363	CTBP1	C-terminal-binding protein 1	0.045349
30 μM	P60709	ACTB	Actin, cytoplasmic 1	0.319082
	P61956	SUMO2	Small ubiquitin-related modifier 2	0.204303
	P07900*	HSP90AA1	Heat shock protein HSP 90-*α*	0.199073
	P24941	CDK2	Cyclin-dependent kinase 2	0.106614
	P61160	ACTR2	Actin-related protein 2	0.103530
	P13569*	CFTR	Cystic fibrosis transmembrane conductance regulator	0.088788
	O15145	ARPC3	Actin-related protein 2/3 complex subunit 3	0.077054
	P04439	HLA-A	HLA class I histocompatibility antigen, A *α* chain	0.076738
	P07550*	ADRB2	*β*-2 adrenergic receptor	0.073300
	Q14957*	GRIN2C	Glutamate receptor ionotropic, NMDA 2C	0.065824

^*^Not present in TPP dataset, added from computational pipeline.

**Table 4:** Sinomenine effects on biological pathways and their clinical rel3nce.

Biological pathways	Clinical rel3nce
*β*2-AR, NMDA, GABA-B receptor modulation	Neuropathic & inflammatory pain, immune modulation in rheumatoid arthritis
Actin cytoskeleton	Synovial fibroblast migration (rheumatoid arthritis), metastasis (cancer)
RNA binding, DNA replication, CDK2	Anti-proliferative effects (cancer)
Metabolic pathway modulation	Immunometabolism in rheumatoid arthritis, cancer cell metabolism

## Discussion

While high-throughput omics strategies, such as TPP and CP, allow for unbiased investigation of drug–target interactions and MoAs, both of these strategies can generate huge sets of potential targets or MoAs. Researchers ultimately need to narrow down the list of hypotheses before proceeding to verify the proposed targets or MoAs through additional experiments, a process that often involves rigorous literature studies or additional omics experiments. Here, we show that by combining information from CP and TPP data into a single PPI network, known targets are likely to appear among the nodes with the highest BCS.

For validation of our computational pipeline, we turned to publicly available datasets. Here, for 29 out of 49 tested compounds, the known “true” targets could be found among either the proteins with the highest BCS; among the overlap between targets for compounds causing similar morphological perturbation in CP and affected proteins in TPP; or often both. Complementing TPP data with other measurable modalities can be critical, because a typical LC–MS analysis can only cover half the proteome, with identification and quantitation of roughly 10,000 out of 20,000 canonical proteins. Hence, conducting TPP alone has a risk of missing true drug targets. We show that by increasing the accessible information of target engagement with CP, the number of hypothetical targets to test was effectively narrowed down to ten or fewer proteins, compared to the 35–64 targets found for the investigated compounds using only TPP. It is important to note that in order for our workflow to detect a target, it has to either show significant thermal shifts in TPP data or be annotated as a known target for compounds with similar morphological changes in CP as the compound under investigation.

While this strategy does not remove the need for further validation experiments on drug–target interactions, we expect it to drastically reduce the time and cost of such validations. In CP, it is often assumed that compounds causing similar morphological changes can be used to infer target and MoA as long as extensive annotations exist for those compounds (Seal et al, 2025). We observed that for the CP data included in this study, compounds with shared targets or MoA could cause vastly different morphological changes. Crizotinib did not display morphological changes similar to other compounds targeting MET or ALK. Notably, there existed a cluster of MET/ALK inhibitors within the CP data, which did not overlap with Crizotinib, suggesting that inhibition of these targets can indeed cause very distinct morphological changes. The high expression of ABL and BCR in K-562 myelogenous leukemia cells (neither of which were detected in the TPP data), along with stabilization of growth factor receptor-bound protein 2 (GRB2), suggests that the observed effects in TPP could be caused by Crizotinib interacting with the Bcr-Abl pathway. In many forms of leukemia, a genetic translocation event can result in the production of the BCR-ABL oncoprotein (Liu et al, 2022). BCR-ABL can recruit GRB2, which mediates Ras/MAPK signaling and can lead to hyperactivation of cell proliferation in leukemia (Liu et al, 2022). On the contrary, the morphological changes observed in U2OS bone cancer cells in CP could be a composite effect of both MET and ABL1 inhibition. MET activation of RAC1 either cause signaling through the mTORC1 pathway leading to cell growth, or lead to actin reorganization and cell migration (Hervieu and Kermorgant, 2020). Both of these pathways were suggested by GO and Reactome enrichment analysis of the Crizotinib PPI network (Appendix Fig. S11), and RAC1 and GRB2 were identified among the top 10 proteins with the highest BCS (Appendix Table S25). Therefore, we conclude that the analysis strategy proposed in this article provides valuable hints about Crizotinib MoA, despite the large discrepancies in target expression between K-562 and U2OS cells.

Nevertheless, the case of Crizotinib highlights the importance of considering the cell line when designing TPP and CP experiments. Integrating CP and TPP data from different cell lines appears to be a viable strategy, primarily when the targets are similarly expressed in both cell lines. Differences in gene expression between cell lines could potentially result in a compound acting on different pathways depending on the genetic makeup of the cell. For this reason, we recommend using the same cell line for both assays if applying this strategy to a compound with unknown targets or MoA.

Our analysis strategy is highly dependent on the clustering of CP data to infer targets and MoAs from compounds with similar morphological perturbations. There exists a plethora of clustering algorithms today. However, many perform poorly or with low reproducibility on data with overlapping or hard-to-distinguish clusters, which is commonly the case in CP data. Popular methods like *k*-mean clustering and HDBSCAN are stochastic methods which can result in very different cluster structures when repeated on the same input data, especially when the number of clusters is large and poorly separated (Chaudhry et al, 2023). Furthermore, having a large number of features negatively affects cluster reproducibility for these methods, and it has been shown that feature reduction prior to clustering improves performance (Hozumi et al, 2021). In contrast, deterministic clustering algorithms, such as agglomerative hierarchical clustering, are computationally heavy and may converge to a local optimum solution rather than global (Chaudhry et al, 2023). The consensus clustering algorithm SC3, and its later Python implementation SC3s, was developed as an attempt to improve reproducibility of *k*-means clustering on single-cell RNA sequencing (scRNA-seq) data (Kiselev et al, 2017). scRNA-seq, similarly to CP, commonly contains noisy, high-dimensional data which poses great challenges for cluster detection and separation (Zhang et al, 2023).

In our study, we observed that SC3s clustering with 2000 runs of *k*-means clustering on 14 principal components gave highly reproducible clusters for (+)-JQ1, I-BET151, Crizotinib, and Panobinostat. For Vemurafenib, on the other hand, reproducibility remained low. This compound had the lowest grit score of the considered compounds. While our proposed analysis strategy still managed to improve both target and MoA predictions compared to only considering CP or TPP data, the poor reproducibility of the cluster for Vemurafenib highlights a drawback with the method. Future developments of the proposed analysis strategy would need to focus on improving the CP clustering, either by considering other consensus clustering algorithms which do not depend on *k*-means, or by incorporating other means of feature selection for CP data. Recent studies have shown improved classification of chemicals in CP when features are extracted using deep learning algorithms compared to CellProfiler (Seal et al, 2025).

Another challenge observed with our method is that, for the cases where a known target was only detected in CP and not PISA, our model failed to include that target in the final network in 7 out of 15 cases (Appendix Fig. S13F). As our computational workflow was intentionally designed to place a larger trust in network nodes derived from TPP/PISA data over CP-derived nodes, this behavior becomes an unfortunate consequence. We also observed, when merging the Van Vranken et al, 2024 PISA data with CP, that the type of statistical strategy used to detect thermal shifts in the PISA data had a direct impact on the success of our model in identifying known targets (Appendix Fig. S13C,D). While a more relaxed strategy may result in more false positive thermal shifts, a too stringent filtering often do not provide enough thermal shifts to construct a PPI network. Advising on best practice for TPP/PISA data pre-processing is outside the scope of this article. However, for further improvements of our method, it would be interesting to explore the use of Bayesian strategies to incorporate the confidence of each thermal shift into the final PPI network.

As a proof-of-principle, we applied our computational pipeline to a compound with less-known MoAs, sinomenine. Our analysis of sinomenine in U2OS cells reveals a multi-target engagement profile that aligns well with its potential clinical applications in rheumatoid arthritis, pain management, and cancer. The thermal stabilization and destabilization of *β*2-adrenergic, NMDA, and GABA-B receptors support sinomenine’s modulation of neuronal signaling pathways relevant for neuropathic and inflammatory pain, providing mechanistic insight into its traditional use for analgesia. The observed impact on actin cytoskeleton organization suggests potential effects on immune cell migration and synovial fibroblast invasiveness, processes central to joint damage in rheumatoid arthritis. Furthermore, the identification of CDK2 and pathways related to RNA binding and DNA replication indicates that sinomenine may exert anti-proliferative effects, providing a rationale for exploring its use in cancer treatment. Modulation of metabolic pathways, including the citric acid (TCA) cycle, further supports a role in altering immunometabolism and cellular energy balance, which may be beneficial in chronic inflammatory and oncogenic contexts. Collectively, these findings expand the mechanistic understanding of sinomenine, supporting its continued investigation as a multimodal therapeutic candidate across inflammation, pain, and cancer indications.

In conclusion, we have presented a novel strategy for improved target and MoA identification that integrates CP and TPP data. This strategy has the potential to aid studies in drug development or drug repurposing. Our results on Crizotinib suggest that the method is capable of highlighting lesser studied perturbed pathways, even in cases where the direct targets are missing from the TPP data. Finally, we characterized a compound with less understood MoA, sinomenine, where our results further highlight mechanisms of its anti-inflammatory and analgesic effects as well as its potential use as an anti-tumor drug. Our findings suggest its involvement in metabolism and cellular architecture, while we also verify previous findings on sinomenine’s activity on ligand-binding plasma membrane receptors.

## Methods

**Table:** Reagents and tools table

Reagent/resource	Reference or source	Identifier or catalog number
**Chemicals for LCMS**		
Sinomenine	Hunan Zhengqing Pharmaceutical	N/A
DMSO	Sigma-Aldrich	D2438
DMEM (High glucose, L-glutamine, pyruvate)	Gibco	11960-044
Fetal bovine serum, heat-inactivated	Gibco	10500064
Penicillin-Streptomycin	Thermo Fisher Scientific	15140122
DPBS, 1×	Gibco	14190250
TrypLE	Gibco	A1217701
Accutase	Sigma-Aldrich	A6964
Halt Protease Inhibitor Cocktail, 100X	Thermo Fisher Scientific	87786
RapiGest SF surfactant	Waters	186008090
BCA Protein Assay Kit	Thermo Fisher Scientific	23227
EasyPep Mini MS Sample Prep kit	Thermo Fisher Scientific	A40006
Trypsin/Lys-C protease mix	Included in EasyPep kit	(kit component)
Digestion Stop Solution	Included in EasyPep kit	(kit component)
Peptide Cleanup Columns	Included in EasyPep kit	(kit component)
Triethylammonium bicarbonate (TEAB), 1M	Thermo Fisher Scientific	90114
Pierce Quantitative Colorimetric Peptide Assay	Thermo Fisher Scientific	23275
TMT11plex Isobaric Label Reagent	Thermo Fisher Scientific	A37725
Acetonitrile (ACN)	Fisher Chemical	A955-1
Water	Thermo Fisher Scientific	W6-1
Hydroxylamine, 50% (v/v)	Thermo Fisher Scientific	90115
Trifluoroacetic acid (TFA), 99.5% (v/v)	Thermo Fisher Scientific	28904
Triethylamine (TEA), 0.1% (v/v)	Thermo Fisher Scientific	1862986
Basic pH Fractionation Kit	Affinisep	BioSPE PepFrac Spin15-PepFrac.H8.25
PBS	Gibco	18912014
Formic acid (FA), 99.0+% (v/v)	Fisher Chemical	A117-50
**Experimental models**		
**Recombinant DNA**		
**Antibodies**		
**Oligonucleotides and other sequence-based reagents**		
**Chemicals for cell painting**		
MycoAlert	Lonza	#LT07-218
DMEM	Gibco	#11960-044
Pencilin-Streptomycin	ThermoFischer	#15140122
FBS	Gibco	#10500064
DPBS	Gibco	#14190250
TrypLE	Gibco	#A12177-01
FluoroBrite DMEM	Gibco	#A1896701
4% PFA	Histolab	#M22426
PBS	Gibco	#18912014
Triton X-100	Thermo Scientific	#85111
MitoTracker Deep Red	Invitrogen	#M22426
Hoechst 33342	Inivitrogen	#H3570
Wheat Germ Agglutinin CF568	Biotium	#29077
Phalloidin CF568	Biotium Conjugate	#29080
SYTO 13	Invitrogen	#S7575
Concanavalin A750	Biotium Conjugate	#29080
**Software**		
Xcalibur v4.7.69.37	Thermo Fisher Scientific	
Orbitrap Fusion Tune v4.2.4321	Thermo Fisher Scientific	
FragPipe v22.0	https://fragpipe.nesvilab.org	
Affinity Designer 2	https://affinity.serif.com/en-us/designer	
cytominer-eval	https://github.com/cytomining/cytominer-eval	
The Human Protein Atlas v.24.0	https://www.proteinatlas.org/	
jupyterlab v.4.2.3	https://jupyter.org/	
matplotlib v.3.9.0	https://matplotlib.org/	
networkx v.3.5	https://networkx.org/	
numpy v.1.26.4	https://numpy.org/	
pandas v.2.3.0	https://pandas.pydata.org/	
Python 3.11.0rc1 programming language	https://www.python.org/	
Requests v.2.32.3	https://requests.readthedocs.io/en/latest/	
scanpy v.1.11.2	https://scanpy.readthedocs.io/en/stable/	
sc3s v.0.1.1	https://github.com/hemberg-lab/sc3s	
seaborn v.0.13.2	https://seaborn.pydata.org/	
sklearn	https://scikit-learn.org/stable/	
STRING db v.11.9	https://string-db.org/	
umap-learn v.0.5.7	https://umap-learn.readthedocs.io/en/latest/	
unipressed v.1.4.0	https://multimeric.github.io/Unipressed/	
**Other**		
Acclaim PepMap 100, 75 μm × 2 cm	Thermo Fisher Scientific	164946
EASY-Spray PepMap RSLC, C18, 2 μm, 100 Å, 75 μm × 500 mm	Thermo Fisher Scientific	ES903
Orbitrap Tribrid Fusion Mass Spectrometer	Thermo Fisher Scientific	N/A
EASY-Spray Ion Source NG	Thermo Fisher Scientific	ES082
EASY-nLC 1000 Liquid Chromatography System	Thermo Fisher Scientific	LC120

### Methods and protocols

#### Compounds

All compounds (5259 test samples and 7 cell painting reference compounds) were used in assay-ready plates. In brief, chemicals solubilized in dimethyl sulfoxide (DMSO) were spotted using Echo Acoustic Liquid Handler into 384 multiwell plates (PhenoPlateTM Revvity #6057302) at specific volumes to achieve a final treatment concentration of 10 μM for test compounds and 6.25, 10 μM or 20  μM for controls. Test compounds were distributed over 80 plates with two technical replicates per dose and two biological replicates (cells from independent cell cultures). Seven phenotypic reference compounds which are known to induce a distinctive phenotype in a variety of cells were included on each compound plate as reference controls. Specifically, these included: Etoposide (Sigma-Aldrich E1383) at 6.25 μM, Fenbendazole (Sigma-Aldrich F5396) at 6.25 μM, Berberine chloride (Sigma-Aldrich Y0001149) at 20 μM, Fluphenazine dihydrochloride (Sigma-Aldrich F0280000) at 20 μM, Tetrandrine (Sigma-Aldrich Y0001165) at 10 μM, CA-074 methyl ester (Sigma-Aldrich 205531) at 20 μM and Sorbitol (Sigma-Aldrich S1876) at 20  μM. Each compound plate contained ~133 test compounds at two technical replicates, 7 reference compounds at two technical replicates, and 22 wells of DMSO (Sigma-Aldrich D2438) used as a compound vehicle at 0.1% concentration. Compounds were dissolved in 20 μl cell culture medium and incubated on a shaker at RT at 150 rpm for 1 h prior to cell seeding. To reduce bias by positional effects in the microplate wells, the conditions were distributed over the plates using PLAID (Plate Layouts using Artificial Intelligence Design) (Francisco Rodríguez et al, 2023).

#### Cell culture

For the purpose of this study, the U2OS human bone sarcoma cell line (sourced from ATCC U2OS #HTB-96) was expanded and painted at passage number +8. The cells were maintained under sterile conditions at 37 °C, 5% (v/v) CO_2_, 95% humidity, and cultured in DMEM (High glucose, L-glutamine, pyruvate) medium (Gibco #11960-044) supplemented with 10% (v/v) fetal bovine serum, heat-inactivated (Gibco #10500064), and 1% (v/v) Penicillin-Streptomycin (ThermoFisher #15140122). After reaching 90% confluency, cells were washed in DPBS 1× (Gibco #14190250), dissociated with TrypLE (Gibco #A1217701), and reseeded in fresh complete medium in T75 flasks (Thermo Scientific #156499). The cells were tested for Mycoplasma using a luminescence-based MycoAlert kit (Lonza #LT07-218). For the cell painting experiment, cells were seeded at a density of 750 cells/well in 20 μL of medium into 384-well plates containing dissolved compounds totaling 40 μL per well, and incubated for 48 h.

#### Cell painting, image acquisition, and feature extraction

Cell painting experiments were conducted according to a protocol previously described (Bray et al, 2016) with minor modifications. A Biotek MultiFlo FX was used for dispensing solutions, and a BlueWasher (Blue Cat Bio) microplate washer was used for washing steps. A robotic arm (UR10) was used to move plates between the incubator, plate hotel, washer, and dispenser, using tailored software for scheduling (more info: https://github.com/pharmbio/aros). In short, 20 μl MitoTracker Deep Red (Invitrogen #M22426) prewarmed in FluoroBrite DMEM (900 nM, Gibco A1896701) was added to the plates. After 30 min of incubation, cells were fixed by the addition of 40 μl of 4% PFA (Histolab #02176) for 30 min. Thereafter, cells were washed two times with 100 μl PBS (Gibco 18912014). Then 20 μl staining mixture containing 0.1% Triton X-100 (Thermo Scientific #85111) was added to each well reaching a final well-concentration of 1 μg/ml Hoechst 33342 (Invitrogen #H3570), 15 μg/ml Wheat Germ Agglutinin CF568 (Biotium #29077), 5 μg/ml Phalloidin CF568 (Biotium #00044), 4 μg/ml SYTO 13 (Invitrogen #S7575) and 41 μg/ml Concanavalin A CF750 (Biotium Conjugate #29080), and was incubated for 30 min. Stains were removed, and plates were washed two times with 100 μl PBS and stored at 4 °C until image acquisition.

Fluorescence microscopy was carried out using a high-throughput Squid system (Cephla), capturing images from nine sites per well across five fluorescence channels. The resulting images underwent processing and analysis with CellProfiler and CellPose 2.0 (Stringer et al, 2021), which included quality control, illumination correction, segmentation, and feature extraction. Segmentation outputs were stored as binary masks and subsequently used in CellProfiler for feature extraction. A feature extraction pipeline was executed using CellProfiler version 4.3.4 (accessible at 10.17044/scilifelab.21378906.v2 and detailed in (Harrison et al, 2023)). Various quality measures were calculated to represent a wide variety of artifacts. Images deviating more than six standard deviations from the mean for FocusScore, PowerLogLogSlope, MaxIntensity, MeanIntensity, and StdIntensity in any of the image channels were flagged, inspected, and removed if necessary. Removed where also blurred images with PowerLogLogSlope values higher than −1, out-of-focus images with FocusScore above 0.4, and images with few cells (StdIntensity below 0.01 and Count nuclei below 20). This pipeline produced ~1500-dimensional feature vector for each cell. The extracted features represent measurements such as fluorescence intensity, spatial location, granularity, and other cellular attributes, categorized into cell, nucleus, and cytoplasm features. Downstream analysis was performed using Python 3. First, the image medians of the features were computed from all cells on the image. Features were normalized relative to the DMSO (control) images by subtracting the median value for DMSO images and then dividing by the median absolute deviation (MAD) of DMSO images. Features that were invariant or close to invariant in DMSO (MAD < 0.0001) were not used for further analysis.

#### Characterization of sinomenine with TPP

U2OS cells were cultured as described above in T75 flasks. Sinomenine was dissolved in DMSO to prepare a 10 mM stock solution. For treatment, 10 and 30 μL of stock solution were diluted in 9.99 or 9.97 mL of culture medium to achieve a final concentration of 10 or 30 μM, respectively. Vehicle control groups received an equivalent volume of DMSO (final solvent concentration ≤0.1%). Following medium replacement with either 10 μM sinomenine, 30  μM sinomenine, or DMSO control, cells were incubated for 1 h at 37 °C and 5% CO_2_. Post-treatment, cells were washed with PBS, detached using accutase, pelleted by centrifugation at 300×*g* for 5 min (4 °C), washed in cold PBS, and resuspended in PBS containing protease inhibitor (10 μL/mL).

Cell suspensions were adjusted to 4 × 10^6^ cells per 100 μL PBS for TPP analysis with the PISA protocol (Gaetani et al, 2019). For each condition (sinomenine-treated and control), 100 μL aliquots were distributed across seven microcentrifuge tubes in triplicates. Tubes were heated for 3 min at designated temperatures: T  ∈ [37, 42, 47, 52, 57, 62, 67] °C using a preheated heating block (Thermomixer Compact, Eppendorf), cooled at room temperature for 3 min, snap-frozen in liquid nitrogen, and stored on ice. Three freeze-thaw cycles were performed. For each replicate, aliquots from all seven temperatures were pooled into protein low-binding tubes and incubated with shaking (350 rpm) at 4 °C for 30 min. To each pooled lysate, 1 μL of RapiGest SF surfactant (Waters, Manchester, UK) was added per 100 μL sample. Lysis was completed by two freeze-thaw cycles (liquid nitrogen freezing followed by thawing at 25 °C), with vortexing after each thaw. Samples were centrifuged at 14,000 × *g* (4 °C, 90 min) to separate soluble proteins from aggregates. The soluble supernatants were transferred to new tubes, and protein concentrations were measured using the BCA assay (Thermo Fisher Scientific).

Protein digestion and labeling were performed using the EasyPep Mini MS Sample Prep kit (Thermo Fisher Scientific) according to the kit protocol. Briefly, 100 μg of protein sample was mixed with Lysis Solution, reduced, alkylated, and heated at 95 °C for 10 min. A trypsin/Lys-C protease mix was then added and incubated at 37 °C for 3 h. Reactions were stopped with Digestion Stop solution. Peptides were subsequently purified using peptide clean-up columns, washed, and eluted as per the protocol. The eluted peptides were dried using vacuum centrifugation and resuspended in 100 μL of 100 mM triethylammonium bicarbonate (TEAB), pH 8.5. Peptide concentrations were determined using the Pierce Quantitative Colorimetric Peptide Assay (Thermo Fisher Scientific). Each 100 μg peptide sample was labeled with 0.8 mg TMT reagent dissolved in 40 μL acetonitrile (ACN), incubated at room temperature for 1 h, and quenched with 5% (v/v) hydroxylamine for 15 min. Labeled samples were pooled in equal amounts and acidified with 5% (v/v) trifluoroacetic acid (TFA) to pH  < 3, then dried down by vacuum centrifugation. The TMT label efficiency was determined to be greater than 99% based on the relative abundance of unlabeled versus labeled peptides.

The multiplexed TMT-labeled sample was dissolved in 0.1% (v/v) TFA and fractionated using a BioSPE PepFrac-SPE spin column (Affinisep) under basic pH conditions. The column was equilibrated with 0.1% (v/v) TFA, and the added sample was washed with water and 4% (v/v) ACN in 0.1% (v/v) triethylamine (TEA). Peptides were eluted in eight fractions using 8–50% (v/v) ACN in 0.1% (v/v) TEA. Each fraction was then dried down by vacuum centrifugation and resuspended in 0.1% (v/v) formic acid (FA) in water.

The TMT-labeled combined sample was analyzed with an Orbitrap Fusion Tribrid mass spectrometer (Thermo Fisher Scientific) coupled to an EASY-nLC 1000 liquid chromatography system (Thermo Fisher Scientific) using a two-column setup: an Acclaim PepMap 100 (2 cm × 75 μm, 3 μm particles, Thermo Fisher Scientific) pre-column connected in front of an EASY-Spray PepMap RSLC C18 reversed phase analytical column (50 cm × 75 μm, 2 μm particles, Thermo Fisher Scientific) heated to 45 °C. Peptides were loaded with 0.1% (v/v) FA (solvent A), then separated using a 120-min linear gradient from 5 to 32% solvent B (0.1% (v/v) FA in ACN), followed by 10 min at 32–90% solvent B and a 10-min hold at 90% solvent B. The flow rate was 300 nL/min.

The mass spectrometer operated in Top Speed data-dependent mode with a spray voltage of 2.2 kV and ion-transfer tube temperature of 300 °C. Full scans (scan range *m/z* 400–1400) were acquired at profile mode in the Orbitrap at a resolution of 120,000 with an automatic gain control (AGC) target of 4.0e5 and a maximum injection time of 50 ms. Peptide ions with an intensity  >5.0e3 were selected for MS2 collision-induced dissociation (CID) fragmentation in the linear ion trap (scan range 400–1200) at a collision energy of 35%, an AGC target of 1.0e4, and a maximum injection time of 50 ms. Data was collected at centroid mode, and a dynamic exclusion period of 60 s was used. For synchronous precursor selection (SPS)-MS3 analysis (McAlister et al, 2014), the top ten MS2 fragment ions were co-isolated and subjected to higher-energy collisional dissociation (HCD), with a collision energy of 65%, and analyzed in the Orbitrap at a resolution of 50,000 (scan range *m/z* 100–500). The AGC target was set to 1.0e5, and the maximum injection time was 120 ms. The data was collected in centroid mode. Instrument performance was monitored with the Promega 6 × 5 LC–MS/MS Peptide Reference Mix (Promega) and evaluated using PReMiS software (version 1.0.5.1, Promega).

Raw MS files were processed using the FragPipe suite (version 22.0) (Kong et al, 2017) with the TMT10 workflow settings. Spectra were searched against the UniProtKB/SwissProt Homo sapiens proteome database (UP000005640, version 2024-11-21) with a maximum of two missed cleavages per peptide. Fixed modifications included carbamidomethylation of cysteine and TMT labeling of lysine and peptide N-termini. Oxidation of methionine and N-terminal acetylation were set as variable modifications. First search peptide Orbitrap tolerance was set to  ±20 ppm, and the MS2 ion trap tolerance was set to 0.5 Da. An in-house contaminants database was also utilized. A decoy search was made against the reversed database, where the false discovery rates (FDR) were controlled at 1% for the peptide-spectrum match (PSM), peptide, and protein levels. The protein output was filtered by removing reverse database hits and potential contaminants. Protein solubility changes were quantified using TMT reporter ion intensities and the TMT-integrator software (Chang et al, 2026) integrated in the FragPipe suite, with the MS3 mass tolerance set to 20 ppm and using median centering normalization.

Statistical analysis was conducted using the limma package (Ritchie et al, 2015) in R to identify proteins with significantly altered solubility profiles. The *P* values were corrected for multiple testing using the Benjamini-Hochberg method (Benjamini and Hochberg, 1995). Proteins were considered significantly thermally affected if the fold change compared to the DMSO control had an adjusted *P* value  < 0.05. No fold-change cutoff was applied.

#### Data retrieval and processing for public TPP and PISA data

For compounds JQ1 and I-BET151, pre-analyzed 2D-TPP data for whole-cell and cell lysate were retrieved from the Mendeley data supplementary materials of publication Savitski et al (Savitski et al, 2018) (10.17632/8pzhg2tdyb.1) supplementary dataset 1. For compounds Vemurafenib and Crizotinib, pre-analyzed ITDR-TPP data were downloaded from the supplementary materials of Savitski et al (Savitski et al, 2014), Appendix Table S8. Pre-analyzed data for Panobinostat were retrieved from the supplementary materials of Franken et al (Franken et al, 2015), supplementary data 2. All of the downloaded datasets had been processed using the R-package TPP (Childs et al, 2026) or equivalent strategies by the original authors.

For Panobinostat, Vemurafenib, and Crizotinib, significantly stabilized and destabilized proteins were selected based on the criteria outlined in their original publications. For JQ1 and I-BET151, selection of proteins was performed as described in the original publication, but with one exception; a protein was only considered if it had at least two concentration curves at neighboring temperatures pass the quality control filter.

Log2-fold changes and calculated nSD for a 96-compound large PISA dataset were retrieved from the supplementary materials of the publication Van Vranken et al (Van Vranken et al, 2024), Fig. 1– Source data 1, 2, and 3. Proteins with significant thermal shifts were either identified using the authors’ empirically established filtering criteria (∣log2FC∣ ≥ 0.2, ∣nSD∣ ≥ 3.5) or calculated using the R-package limma (Ritchie et al, 2015), where each compound duplicate was compared to all 64 DMSO controls across all 32 batches while accounting for batch effects in the design matrix. The *P* values were corrected for multiple testing using the Benjamini–Hochberg method (Benjamini and Hochberg, 1995). Only thermal shifts with an adjusted *P* value  <0.01 were considered. No fold-change cutoff was applied.

#### Protein localization and mRNA expression

Subcellular location for 17,025 proteins, based on immunofluorescence (ICC-IF) and confocal microscopy, was retrieved from the Human Protein Atlas database (proteinatlas.org) (Thul et al, 2017) (retrieval date: 2024-10-29). Cell line-specific mRNA expression profiles for 1206 human cell lines were downloaded from the Human Protein Atlas database (Jin et al, 2023) (retrieval date: 2024-10-21).

#### Subcellular location in TPP data

For each protein identified in TPP data, all of its corresponding subcellular locations ("main location” and “additional location”) were extracted from the Human Protein Atlas database. Subcellular locations were grouped into nine categories and plotted as lollipop charts: (i) “Nucleus”, consisting of HPA annotations “nuclear membrane”, “nucleoli”, “nucleoli fibrillar center”, “nucleoli rim”, “nucleoplasm”, “nuclear speckles”, “nuclear bodies”, “mitotic chromosome”, and “kinotochore”. (ii) “Cytosol”, consisting of HPA annotations “aggresome”, “cytoplasmic bodies”, “cytosol”, and “rods and rings”. (iii) “Mitochondria”, consisting of HPA annotation “mitochondria”. (iv) “Endoplasmic reticulum”, consisting of HPA annotation “endoplasmic reticulum”. (v) “Golgi”, consisting of HPA annotation “Golgi apparatus”. (vi) “Actin filament and intermediate filament”, consisting of HPA annotations “actin filaments”, “cleavage furrow”, “focal adhesion sites”, and “intermediate filaments”. (vii) “Centrosome and microtubules”, consisting of HPA annotation “centriolar satellite”, “centrosome”, “cytokinetic bridge”, “microtubule ends”, “microtubules”, “midbody”, “midbody ring”, and “mitotic spindle”. (viii) “Vesicles”, consisting of HPA annotations “endosome”, “lipid droplets”, “lysosome”, “peroxisomes”, and “vesicles”. (ix) “Plasma membrane”, consisting of HPA annotations “cell junctions” and “plasma membrane”. For each category, the number of proteins in each subcellular location was counted. If a protein belonged to more than one category, it was counted once per category.

#### Subcellular location in CP data

The Cell Profiler extracted feature z-scores in the CP data were summarized into subcellular locations based on the staining chemicals used and the object where the stain is predicted to occur (either whole cell or nucleus). Features were grouped into categories based on two criteria: (i) Cell Profiler module, i.e., Intensity (I), Correlation (C), Granularity (G), Location (L), and RadialDistribution (RD); and (ii) stains, i.e., Nucleus (Hoechst), ER (Concanavalin A), Nucleoli and cytoplasmic RNA (SYTO14), Golgi apparatus and F-actin cytoskeleton (WGA and Phalloidin) and Mitochondria (Mitotracker). Features were only considered for the object Cell, except for features from the stain for Nucleus, which were only considered in the object Nucleus. Additionally, area-shape related features were grouped by cell compartment, i.e., Cell (C), Cytoplasm (Cy), and Nucleus (N). The median z-score in each category were plotted as radar charts indicating a positive or negative change in z-score for each category.

#### Grit scores in CP data

Grit scores were calculated for each compound and replicate using the cytominer-eval Python package (https://github.com/cytomining/cytominer-eval) as previously described (Trapotsi et al, 2022).

#### TPP and CP data integration workflow and PPI network construction

1. All significantly perturbed (stabilized or destabilized) proteins from the TPP experiment were summarized into a list (referred to here as TPP-only proteins).
2. PPI networks were retrieved for the list of TPP proteins using the STRING db (Szklarczyk et al, 2023) https://version-11-9.string-db.org/api, specifying physical networks with a minimum score of 150. The species parameter was set to homo sapiens (NCBI identifier: 9606) and “add_nodes” set to 0. The networks were visualized in Python using the NetworkX package (Hagberg et al, 2008) and colored by either: subcellular location (retrieved from HPA); protein stabilization/destabilization in TPP; or cell line expression (retrieved from HPA).
3. Betweenness centrality scores (BCSs) for the TPP-only PPI network (Freeman, 1977) were calculated using the NetworkX package (Hagberg et al, 2008).
4. The cell painting (CP) data were clustered using the unsupervised clustering Python package SC3, Single Cell Consensus Clustering with speed (Quah and Hemberg, 2022). SC3s was originally developed for clustering single-cell RNA sequencing data, and builds on a consensus clustering algorithm wrapped around *k*-means clustering. For the CP data, features were first reduced using principal component analysis to 14 principal components, followed by SC3s run using five different *k* number of clusters (*k* = 100, 110, 120, 150, 180) at 2000 repeated runs for each value of *k* using a batch size of 2000. For each investigated *k*, the cluster containing the compound of interest was identified, and the cluster sizes ranked. Finally, the smallest cluster for all considered values of *k*, containing at least 20 compounds, was selected and visualized using t-Distributed Stochastic Neighbor Embedding (t-SNE). The reproducibility of this clustering strategy was assessed by repeating the clustering workflow described above ten times with different random seeds. Reproducibility was calculated as the percentage of samples present in the cluster for at least nine out of ten repeats. Cluster cohesion and separation were evaluated using Silhouette scores (Rousseeuw, 1987).
5. For each CP cluster containing a compound of interest, known target information was retrieved from the Compound Center portal database (see data availability below) for all compounds within the cluster. If target information was available for the investigated compound (which was the case for all investigated compounds except sinomenine), then the target information of that compound was blinded in the database. A list was constructed containing one instance of each unique target detected within the cluster (referred to here as CP-only proteins). Similarly, mode of action (MoA) information was retrieved from the Compound Center portal database for each compound in the cluster and visualized using bar charts.
6. A new PPI network was retrieved from STRING db based on a list containing both the TPP-only and CP-only proteins using the settings described above. The retrieved network or networks from the STRING db only contain proteins which are directly physically connected with a confidence score above 0.15. Hence, the obtained networks rarely contain all submitted proteins. BCSs were calculated for each connected protein in the largest obtained PPI network. This network is here referred to as the CP-TPP combined network.
7. CP-only proteins were further filtered by a process that ranked all CP-only proteins in the CP-TPP combined network based on BCS and iteratively removed the CP-only protein node with the lowest BCS as long as the removal of that node did not affect the number of connected TPP-only protein nodes in the network. For each iterative removal of a CP-only protein node, a new PPI network was retrieved from STRING db based on all TPP-only proteins plus the remaining CP-only proteins. A final PPI network was generated for all TPP-only proteins and the filtered CP-only proteins (referred to as the final combined network).
8. Target prediction was evaluated for TPP-only networks, CP-only networks, and the final combined network using Receiver Operating Characteristic (ROC) curves using BCSs as ranks. ROC curves were generated using the RocCurveDisplay module from the scikit-learn Python package (Pedregosa et al, 2011). True targets for each compound (except sinomenine) were defined based on a literature search.
9. The final combined network was classified into communities using Clauset–Newman–Moore greedy modularity maximization (Clauset et al, 2004) using the NetworkX package (Hagberg et al, 2008). For each community, enrichment analysis was retrieved from STRING db for Gene Ontology (GO) and Reactome annotations.

## Supplementary information

Peer Review File

Appendix

Dataset EV1

Source data Fig. 2

Source data Fig. 3

Source data Fig. 4

Source data Fig. 5

Expanded View Figures

## Data Availability

The code and generated PISA datasets are available at: The proteomic data for sinomenine is available at https://www.ebi.ac.uk/pride/archive/projects/PXD065743. Jupyter notebooks and R code for data analysis and generated figures: GitHub (https://github.com/camilla-johansson/integrate-cp-tpp). The Compound Center portal database is available at https://compoundcenter.scilifelab.se/file/annotations_excel. The source data of this paper are collected in the following database record: biostudies:S-SCDT-10_1038-S44320-026-00214-9.
