# Supplementary material for: Cell painting and thermal proteome profiling for inference of drug targets and mechanism of action
Source: Mol Syst Biol. 2026 May 15;22(8):1270–91. doi: 10.1038/s44320-026-00214-9 (PMC13434728; doi:10.1038/s44320-026-00214-9)
Supplement: Supplementary file 1 [file 44320_2026_214_MOESM1_ESM.pdf]

molecular systems biology

Cell Painting and Thermal Proteome Profiling for Inference of Drug Targets and Mechanism of Action

Camilla Johansson, Martin Johansson, Phanindra Kasi, Mårten Larsson, Per-Johan Jakobsson, Ulf Göransson, Jordi Carreras Puigvert, Ola Spjuth, and Erik Jansson

Corresponding author(s): Erik Jansson (erik.jansson@uu.se)

Review Timeline:

Submission Date:	8th Jul 25
Editorial Decision:	14th Aug 25
Revision Received:	2nd Dec 25
Editorial Decision:	18th Dec 25
Revision Received:	8th Apr 26
Editorial Decision:	16th Apr 26
Revision Received:	17th Apr 26
Accepted:	21st Apr 26

Editor: Jingyi Hou

Transaction Report:

(Note: With the exception of the correction of typographical or spelling errors that could be a source of ambiguity, letters and reports are not edited. Depending on transfer agreements, referee reports obtained elsewhere may or may not be included in this compilation. Referee reports are anonymous unless the Referee chooses to sign their reports.)

14th Aug 2025

Manuscript Number: MSB-2025-13221
Title: Cell Painting and Thermal Proteome Profiling for Inference of Drug Targets and Mechanism of Action
Author: Camilla Johansson
Martin Johansson
Phanindra Kasi
Mårten Larsson
Per-Johan Jakobsson
Ulf Göransson
Jordi Carreras Puigvert
Ola Spjuth
Erik Jansson

Dear Dr. Jansson,

Thank you for submitting your work to Molecular Systems Biology. We have now heard back from two of the three reviewers who agreed to evaluate your manuscript. Unfortunately, after a series of reminders we did not manage to obtain a report from Reviewer #1. In the interest of time, and since the recommendations of the other two reviewers are quite similar, I prefer to make a decision now rather than further delaying the process. If we receive the comments from Reviewer #1, we will send them to you, and you can address the issues raised by Reviewer #1 together with those raised by the other two reviewers. You will see from the comments below that Reviewers #2 and #3 find the manuscript to be of interest. They raise, however, several important points, which should be convincingly addressed in a revision of this work.

I think that the reviewers' recommendations are rather clear and there is no need to reiterate their comments. Importantly, the concerns regarding benchmarking and ablation analyses should be thoroughly addressed, and additional methodological details should be provided as suggested by the reviewers to enhance the clarity of the study.

All other issues raised by the reviewers need to be satisfactorily addressed as well. As you may already know, our editorial policy allows in principle a single round of major revision, so it is essential to provide responses to the reviewers' comments that are as complete as possible. Please feel free to contact me in case you would like to discuss in further detail any of the issues raised by the reviewers.

On a more editorial level, we would ask you to address the following issues:

- Please provide a .docx formatted version of the manuscript text (including legends for main figures, EV figures and tables). Please make sure that the changes are highlighted to be clearly visible.

- Please provide individual production quality figure files as .eps, .tif, .jpg (one file per figure).

-Please provide a .docx formatted letter INCLUDING the reviewers' reports and your detailed point-by-point responses to their comments. As part of the EMBO Press transparent editorial process, the point-by-point response is part of the Review Process File (RPF), which will be published alongside your paper.

-Please note that all corresponding authors are required to supply an ORCID ID for their name upon submission of a revised manuscript.

-We replaced Supplementary Information with Expanded View (EV) Figures and Tables that are collapsible/expandable online (see examples in http://msb.embopress.org/content/11/6/812). A maximum of 5 EV Figures can be typeset. EV Figures should be cited as 'Figure EV1, Figure EV2" etc... in the text and their respective legends should be included in the main text after the legends of regular figures.

Additional Tables/Datasets should be labeled and referred to as Table EV1, Dataset EV1, etc. Legends have to be provided in a separate tab in case of .xls files. Alternatively, the legend can be supplied as a separate text file (README) and zipped together with the Table/Dataset file.
For the figures and tables that you do NOT wish to display as Expanded View figures, they should be bundled together with their legends in a single PDF file called *Appendix*, which should start with a short Table of Content. Each legend should be below the corresponding Figure/Table in the Appendix. Appendix figures and tables should be referred to in the main text as: "Appendix Figure S1, Appendix Figure S2, Appendix Table S1" etc. See detailed instructions regarding expanded view here: https://www.embopress.org/page/journal/17444292/authorguide#expandedview.

-Before submitting your revision, primary datasets (and computer code, where appropriate) produced in this study need to be deposited in an appropriate public database (seehttp://msb.embopress.org/authorguide - dataavailability https://www.embopress.org/page/journal/17444292/authorguide#dataavailability).
Please remember to provide a reviewer password if the datasets are not yet public.
The accession numbers and database should be listed in a formal "Data Availability" section (placed after Materials & Method) that follows the model below (see also https://www.embopress.org/page/journal/17444292/authorguide#dataavailability). Please note that the Data Availability Section is restricted to new primary data that are part of this study.
Data availability
The datasets (and computer code) produced in this study are available in the following databases:
- RNA-Seq data: Gene Expression Omnibus GSE46843 (https://www.ncbi.nlm.nih.gov/geo/query/acc.cgi?acc=GSE46843)
- [data type]: [name of the resource] [accession number/identifier/doi] ([URL or identifiers.org/DATABASE:ACCESSION])
*** Note - All links should resolve to a page where the data can be accessed. ***

- At EMBO Press we ask authors to provide source data for the main manuscript figures. You will receive a separate email with instructions for providing source data with your revised manuscript, including how to upload and organize the files.

Additional information on source data and instruction on how to label the files are available

- Our journal encourages inclusion of *data citations in the reference list* to directly cite datasets that were re-used and obtained from public databases. Data citations in the article text are distinct from normal bibliographical citations and should directly link to the database records from which the data can be accessed. In the main text, data citations are formatted as follows: "Data ref: Smith et al, 2001". In the Reference list, data citations must be labeled with "[DATASET]". A data reference must provide the database name, accession number/identifiers and a resolvable link to the landing page from which the data can be accessed at the end of the reference. Further instructions are available at .

- We updated our journal's competing interests policy in January 2022 and request authors to consider both actual and perceived competing interests. Please review the policy https://www.embopress.org/competing-interests and update your competing interests if necessary.
Please use the heading "Disclosure statement and competing interests".

- All Materials and Methods need to be described in the main text using our 'Structured Methods' format. According to this format, the Methods section includes a Reagents and Tools Table (listing key reagents, experimental models, software and relevant equipment and including their sources and relevant identifiers) followed by a Methods and Protocols section describing the methods, ideally using a step-by-step protocol format. The aim is to facilitate adoption of the methodologies across labs. Please download and fill our Reagents and Tools Table template (.docx), which you can find in our author guidelines: https://www.embopress.org/page/journal/17444292/authorguide#structuredmethods.
When submitting your revised manuscript, please do not include the Reagents and Tools Table in the Methods section of the manuscript but upload it as a separate file choosing the file type "Reagent Table".

An example of a Method paper with Structured Methods can be found here:
https://www.embopress.org/doi/10.15252/msb.20178071.

-Regarding data quantification:
Please ensure to specify the name of the statistical test used to generate error bars and P values, the number (n) of independent experiments (please specify technical or biological replicates) underlying each data point and the test used to calculate p-values in each figure legend. Discussion of statistical methodology can be reported in the materials and methods section, but figure legends should contain a basic description of n, P and the test applied.
Graphs must include a description of the bars and the error bars (s.d., s.e.m.).
Please also include scale bars in all microscopy images.

- Please provide a "standfirst text" summarizing the study in one or two sentences (approximately 250 characters, including space), three to four "bullet points" highlighting the main findings and a "synopsis image" (550px width and 400-600 px height, PNG format) to highlight the paper on our homepage.
Here are a couple of examples:
https://www.embopress.org/doi/10.15252/msb.20199356
https://www.embopress.org/doi/10.15252/msb.20209475
https://www.embopress.org/doi/10.15252/msb.209495

When you resubmit your manuscript, please download our CHECKLIST (https://www.embopress.org/pb-assets/embo-site/EMBO%20Press%20Author%20Checklist-1642513524327.xlsx) and include the completed form in your submission. *Please note* that the Author Checklist will be published alongside the paper as part of the transparent process (https://www.embopress.org/page/journal/17444292/authorguide#transparentprocess).

If you feel you can satisfactorily deal with these points and those listed by the referees, you may wish to submit a revised version

of your manuscript. Please attach a covering letter giving details of the way in which you have handled each of the points raised by the referees. A revised manuscript will be once again subject to review and you probably understand that we can give you no guarantee at this stage that the eventual outcome will be favorable.

I look forward to receiving the revised manuscript soon.

Sincerely,
Jingyi

Jingyi Hou, PhD
Senior Editor
Molecular Systems Biology

We realize that it is difficult to revise to a specific deadline. In the interest of protecting the conceptual advance provided by the work, we recommend a revision within 3 months (12th Nov 2025). Please discuss the revision progress ahead of this time with the editor if you require more time to complete the revisions.

As a matter of course, please make sure that you have correctly followed the instructions for authors as given on the submission website.

*** PLEASE NOTE *** As part of the EMBO Press transparent editorial process initiative (see our Editorial at https://dx.doi.org/10.1038/msb.2010.72), Molecular Systems Biology publishes online a Review Process File with each accepted manuscripts. This file will be published in conjunction with your paper and will include the anonymous referee reports, your point-by-point response and all pertinent correspondence relating to the manuscript. If you do NOT want this File to be published, please inform the editorial office at contact@molsystbiol.org within 14 days upon receipt of the present letter.

Reviewer #2:

Johansson and colleagues present a workflow to characterize the mechanism of action (MoA) of drugs by integrating Cell Painting data, Thermal Proteome Profiling (TPP), and protein-protein interaction networks derived from prior knowledge sources. The manuscript is concise and generally well-written. A major contribution lies in applying the workflow to generate hypotheses on the MoA of Sinomenine, which, based on a brief literature search, appears to be an NF-κB inhibitor. However, this hypothesis is not experimentally validated or explored in depth. While the methodology is tested on previously published data, I believe the manuscript is not yet ready for publication in *Molecular Systems Biology*. With major revisions, particularly in terms of methodological clarity and systematic validation, the work could become a valuable contribution. As a systems biologist, my comments below focus more on the integration of data modalities and methodological rigor, rather than on expanding the experimental validation.

- The authors should acknowledge prior work that combines TPP with network analysis, particularly the study published in *Molecular Systems Biology* (https://pmc.ncbi.nlm.nih.gov/articles/PMC10987601/). This earlier work demonstrates the feasibility of using such integration for MoA discovery. Therefore, the conceptual novelty claimed in the present manuscript should be revised. The main innovative aspect here appears to be the integration of Cell Painting data, which should be emphasized as the unique contribution of this study.

- The structure of the manuscript would benefit from reordering. Currently, the application of the workflow to Sinomenine precedes the methodology's validation, which can confuse the reader. It would be more effective to first explain the conceptual foundation of the workflow, then present its validation using established data, and only afterwards show its application to a compound with an unclear or debated MoA. This reordering would help clarify the logical progression and the robustness of the approach.

- The methodology section needs a clearer explanation and a better schematic. From my reading, the workflow integrates four key data types: TPP data, prior knowledge networks (such as STRING), Cell Painting profiles, and expression data used to contextualize the PPis. These form the inputs to the workflow. The outputs include ranked lists of target proteins based on network metrics like betweenness centrality, and associated MoA hypotheses based on pathway enrichment. A schematic that

explicitly reflects this input-output structure would enhance understanding.

- The manuscript is rich in descriptive results but lacks quantitative evaluation. To rigorously assess the method's performance, metrics such as AUROC, AUPRC, and F1-score should be introduced. For this, expected true positives in terms of known targets and enriched pathways need to be clearly defined, ideally in a supplementary table. This will allow the authors to benchmark how well the workflow identifies known elements of drug MoA, both at the protein and pathway levels, and to compare these results across input combinations.

- A thorough benchmarking and ablation analysis is critical for the manuscript to meet the standards of this journal. It should be clearly demonstrated how each component-TPP data, Cell Painting, network knowledge, expression contextualization-contributes to the overall prediction quality. For example, the authors should explore what happens to performance when using only TPP data or only clustering from Cell Painting. Similarly, the effect of network contextualization should be tested by comparing STRING-based networks to data-driven alternatives such as cross-linking networks or tissue-specific correlation-based networks (e.g., from https://www.nature.com/articles/s41587-025-02659-z). Furthermore, alternative network ranking methods such as network propagation or Steiner tree algorithms should be evaluated. All of these analyses should be supported by accuracy metrics, providing a clear picture of which components are essential and which may be redundant or detrimental.

In summary, while the manuscript presents an exciting and timely approach to MoA prediction, it requires substantial revisions. The novelty claims should be adjusted to reflect prior work, the structure should be reorganized for clarity, the methodology should be better explained and visualized, and quantitative evaluation and benchmarking need to be systematically introduced. With these improvements, the manuscript could be a strong candidate for publication.

Reviewer #3:

Johansson et al. present a novel graph-based approach that integrates Cell Painting and Thermal Proteome Profiling data to better identify drug targets. The methodological innovation is both timely (given the growing interest in multimodal profiling) and compelling. The manuscript is generally well written and well organized, though several sections would benefit from improved clarity and additional explanation. I have a few major and mostly minor comments that primarily pertain to enhancing the readability of the work.

Major comments

- The integration strategy between the two data modalities is not sufficiently described. In particular, the manuscript introduces the combined network without explaining how the Cell Painting features are incorporated into the network construction. Moreover, the mathematical or algorithmic details of the integration procedure are not described in the Methods section. Given that the integration method represents the main contribution of the work, it is essential that this process be described in a reproducible and transparent manner. I recommend expanding both the Results and Methods sections to include a clear explanation of how the datasets were aligned, how features were combined, and what graph-based algorithms or similarity metrics were applied. Figure 1 presents a high-level understanding, but a deeper dive is needed for this manuscript.
- The statement that "the choice of community detection algorithm has a large impact on what potential mechanisms of action (MoAs) are identified in this step" is concerning. High sensitivity to the choice of algorithm raises questions about the robustness and reproducibility of the findings. It would be helpful if the authors could quantify this sensitivity (e.g., how much do the identified communities and MoA candidates vary across different algorithms?) Additionally, the potential for "cherry picking" results should be addressed explicitly, perhaps by discussing how algorithm choice was made and whether multiple algorithms were systematically compared. This would also strengthen confidence in the cited literature supporting their approach.

Minor comments
- Throughout the manuscript, the authors refer to "Cell Painting features," but this term is ambiguous. There are different methods to derive features from Cell Painting data, and it is not immediately clear which method was used. Only in the methods section is it clarified that the features were extracted using CellProfiler. I recommend briefly specifying this earlier in the main text to improve clarity for readers less familiar with the technical details of image-based profiling.
- The Results section would benefit from a more detailed and quantitative characterization of the specific contribution made by the Cell Painting data. While the metaphor of "filling gaps" is helpful, it should be supplemented with a clearer technical explanation of how CP features enhance or complement the TPP-derived network. For instance, are CP features used to infer new edges, refine existing relationships, or improve prioritization? Additionally, in the Discussion section, the authors state: "Here, we show that by combining CP and TPP data we were able to substantially narrow down the list of possible drug targets." However, the quantitative basis for this claim is not clearly presented in the Results section. For example, while it is mentioned in the Discussion that the known target appears within the top ten, this alone does not convincingly support the strength of the integration. It would be helpful to report the actual number of candidates considered before and after integration, and to provide similar metrics for CP alone. This would allow readers to assess the added value of combining modalities versus using TPP or CP data in isolation. Such an analysis would substantially strengthen the manuscript's core claim.
- While it may be beyond the current scope of the manuscript, it could be valuable to consider how the integration method might perform across different TPP protocols, given the variability in experimental design (e.g., number of temperature points,

compound concentrations, or cell types used). At the very least, I recommend expanding the Discussion section to acknowledge these protocol differences and speculate on whether and how the method would need to be adapted. This would help clarify the generalizability of the approach and guide future applications across diverse experimental setups.
- It would be helpful to introduce the known targets and pathways associated with JQ1 and I-BET151 earlier in the manuscript. These compounds are bromodomain inhibitors, but this important contextual information is not provided until later. Including this detail upon first mention (especially in the Results section) would improve clarity for readers who may not be familiar with these compounds and better contextualize the downstream analyses.
- The following statement in the Results section is quite informative but may benefit from clearer presentation: "Preliminary clustering attempts for the CP data using k-means, HDBSCAN, and agglomerative clustering showed that the tightest clusters could be achieved using HDBSCAN with a prior principal component analysis (PCA) feature reduction, but this approach also resulted in the largest variation in cluster size and a high risk that the compound of interest would not be included in any cluster at all. k-means clustering on PCA data with between 100 and 300 k clusters performed similarly to agglomerative clustering using the same number of clusters as input parameters." This is an important methodological comparison and could be clarified and strengthened by including a dedicated figure that visually compares the performance of the different clustering approaches (e.g., cluster size distributions, number of unassigned compounds, or some clustering metric like silhouette score). Although Figure S1 is cited, it does not directly illustrate this analysis. A new supplemental or main figure focused on this comparison would help readers interpret and contextualize the choice of clustering strategy.
- Several figures and tables would benefit from clarification to improve interpretability:
o Figure 2B: The text states that a cluster "behaves similarly," yet the blue points in the t-SNE plot appear widely distributed. It would be helpful if the authors clarified what "behaves similarly" means in this context, and how this is visually supported by the figure.
o Figure 2C: The figure is very dense, and many protein names are difficult to read due to overlapping text and font size. In addition, the authors should clarify what the point sizes and line lengths represent.
o Table 1: The meaning of the asterisk ("Added from CP cluster") is unclear. Does this mean these targets would not have been identified using TPP data alone? Would the table be identical without Cell Painting data, minus the asterisked entries? Clarifying whether the TPP-based rankings change after integration with CP would help readers understand the added value of the multimodal approach. Moreover, the section discussing Table 1 mentions these additional targets but does not elaborate on why Cell Painting, rather than TPP, identifies them. A brief discussion of whether this is expected (e.g., what biological properties CP captures that TPP may miss) would be helpful.
o Figure 3D: The quantification method used to generate this radar plot should be described more clearly. What is the measurement? It is unclear whether the volume formed by the lighter blue region has specific meaning, given that the spokes of a radar plot have no inherent ordering. Additionally, the meanings of "pos" and "neg" in this figure are not explained and should be defined either in the figure or accompanying legend.
o Figure 4A: The color contrast in this panel is suboptimal. The various shades of blue used for different clusters are too similar to distinguish easily, and the dark orange point mentioned in the text is nearly invisible. Adjusting the color palette to improve accessibility and contrast would enhance readability.
o Figure 4C-D: The sentence "Figure 4C-D show PPI networks for all identified TPP nodes combined with the six versus eight selected CP nodes" is difficult to follow. Please clarify whether the two panels represent different node combinations, different compound treatments, or different analysis strategies.
- The gene ontology and Reactome enrichment analysis in Figure 5B is compelling, particularly the proposed mechanism whereby (+)-JQ1 acts through lysine-acetylated histone binding, potentially leading to cell death and alterations in lipid metabolism. However, the manuscript does not clearly explain how the enrichment analysis supports the inferred ordering of pathway activation. It would strengthen the interpretation to clarify whether these results suggest a causal sequence or if this is just conjecture.
- The authors attribute the reproducibility of Vemurafenib to the fact that its morphological profile did not fall within visually separated clusters (Figure S4E). However, upon inspection, Figure S4E appears to show distributions similar to those of other compounds highlighted in Figure S4D, Figure 4A, and Figure 3E. This raises some uncertainty about the basis for this explanation. It would be helpful if the authors could clarify what distinguishes Vemurafenib's profile in these visualizations and provide additional evidence supporting its unique reproducibility characteristics.
- Table 4 presents a key result showing whether the known target is present in the identified cluster. However, simply stating "Yes, the target is in the cluster" may not be a sufficiently informative metric. For instance, if most or all compounds appear in the clusters, this metric lacks specificity and may overstate performance. It would be valuable to include measures of sensitivity, specificity, or other relevant classification metrics to better assess the accuracy and robustness of target identification. Additionally, the phrase "model predicts target or mechanism of action (MOA)" is used without a clear definition of what the model entails or how predictions are generated. Clarifying the nature of this model and the prediction methodology would improve transparency and help readers interpret these results appropriately.

We thank the reviewers for taking their time to read and comment on our manuscript. We believe that the suggestions from the reviewers has helped us to improve the manuscript and hope that it is now well fit for publication in MSB.

*** PLEASE NOTE *** As part of the EMBO Press transparent editorial process initiative (see our Editorial at https://dx.doi.org/10.1038/msb.2010.72), Molecular Systems Biology publishes online a Review Process File with each accepted manuscripts. This file will be published in conjunction with your paper and will include the anonymous referee reports, your point-by-point response and all pertinent correspondence relating to the manuscript. If you do NOT want this File to be published, please inform the editorial office at contact@molsystbiol.org within 14 days upon receipt of the present letter.

Reviewer #2:

Johansson and colleagues present a workflow to characterize the mechanism of action (MoA) of drugs by integrating Cell Painting data, Thermal Proteome Profiling (TPP), and protein-protein interaction networks derived from prior knowledge sources. The manuscript is concise and generally well-written. A major contribution lies in applying the workflow to generate hypotheses on the MoA of Sinomenine, which, based on a brief literature search, appears to be an NF-κB inhibitor. However, this hypothesis is not experimentally validated or explored in depth. While the methodology is tested on previously published data, I believe the manuscript is not yet ready for publication in *Molecular Systems Biology*. With major revisions, particularly in terms of methodological clarity and systematic validation, the work could become a valuable contribution. As a systems biologist, my comments below focus more on the integration of data modalities and methodological rigor, rather than on expanding the experimental validation.

- The authors should acknowledge prior work that combines TPP with network analysis, particularly the study published in *Molecular Systems Biology* (https://pmc.ncbi.nlm.nih.gov/articles/PMC10987601/). This earlier work demonstrates the feasibility of using such integration for MoA discovery. Therefore, the conceptual novelty claimed in the present manuscript should be revised. The main innovative aspect here appears to be the integration of Cell Painting data, which should be emphasized as the unique contribution of this study.

Answer: We thank the reviewer for highlighting important previous literature that is relevant for our study. We have added the suggested reference: "…and TPP has been integrated with differential expression proteomics and transcriptomics [14]".

We next indicate that the approach to merge information from CP and TPP has been tried to some extent before, but with limitations.

"Here, we merge information from CP with TPP to combine the power of morphological characterization in CP with the detection of target and pathway perturbation in TPP. To our knowledge, the integration of CP with TPP has only been attempted twice before [8,15], and were limited to comparing putative targets identified with both methods."

- The structure of the manuscript would benefit from reordering. Currently, the application of the workflow to Sinomenine precedes the methodology's validation, which can confuse the reader. It would be more effective to first explain the conceptual foundation of the workflow, then present its validation using established data, and only afterwards show its application to a compound with an unclear or debated MoA. This reordering would help clarify the logical progression and the robustness of the approach.

Answer: We thank the reviewer for suggesting an improvement of the logical flow of our study. The results for sinomenine have now been placed after the validation of the computational pipeline, and the discussion now follows the same order with a discussion of our findings of sinomenine at the end.

- The methodology section needs a clearer explanation and a better schematic. From my reading, the workflow integrates four key data types: TPP data, prior knowledge networks (such as STRING), Cell Painting profiles, and expression data used to contextualize the PPIs. These form the inputs to the workflow. The outputs include ranked lists of target proteins based on network metrics like betweenness centrality, and associated MoA hypotheses based on pathway enrichment. A schematic that explicitly reflects this input-output structure would enhance understanding.

Answer: We thank the reviewer for this feedback. The methods section has been reorganized and expanded to contain a step-by-step description of the integration workflow. Please see pages 11–15. In particular, we have provided a step-by-step enumerated list for the CP-TPP data integration, please see page 15. Furthermore, Figure 1 has been updated for clarity.

- The manuscript is rich in descriptive results but lacks quantitative evaluation. To rigorously assess the method's performance, metrics such as AUROC, AUPRC, and F1-score should be introduced. For this, expected true positives in terms of known targets and enriched pathways need to be clearly defined, ideally in a supplementary table. This will allow the authors to benchmark how well the workflow identifies known elements of drug MoA, both at the protein and pathway levels, and to compare these results across input combinations.

Answer: We understand what the reviewer is requesting, however we do not frame the method as a supervised classification problem, but rather an unsupervised, and hence it does not allow for most of the suggested quantitative metrics. Further, it is important to take into consideration that this project is based on datasets from only five drugs, and where ground truth comes from target annotations in literature that are valuable but almost certainly incomplete. The target (and off-target) annotations for each compound that we found in literature are summarized in table 4. We have further extended the analysis by including supplementary tables showing all betweenness centrality scores for networks containing only proteins from TPP data and only proteins from CP clusters. ROC curves that use betweenness centrality scores for ranking have been constructed for TPP, CP and integrated CP-TPP (new figure EV1), which show that target identification improves in all

cases except Crizotinib when using both CP and TPP combined. In the main text, we now describe this:

“For Panobinostat, TPP alone detected three out of eleven known targets (HDAC6, HDAC8, and HDAC10, table S18-S19). CP cluster analysis added another eight known targets (HDAC1, HDAC2, HDAC3, HDAC4, HDAC5, HDAC7, HDAC9, and HDAC11, Figure S6F and Table S20). While CP cluster data contained the highest incident of known targets (Table S20), the targets were not necessarily found among the proteins with highest BCSs, which gave a poor target prediction when using CP data alone (AUC = 0.55, Figure EV1). Combining TPP and CP resulted in six targets being identified (HDAC1, HDAC3, HDAC5, HDAC6, HDAC8, and HDAC10), with four of these being found among the top ten highest BCSs (Figures EV1, S6G and Table S21). GO enrichment analysis suggested histone deacetylase activity as an affected pathway (Figure S7, Community 1) along with a large set of affected signal transductions and dysregulation of metabolic processes. Several components of the 26S proteasome (PSMC1, PSMC4, PSMC5, PSMD1, PSMD8, PSMD11 and PSMD13) were found to be destabilized (Table S18 and S21).”

We have, however, not included any list of “ground truth” for MoA, as this is arguably a lot more challenging to define for each compound, especially in terms of GO annotations. One important point is that there is generally a lack of large enough multi-omics datasets (cell morphology and proteomics) with associated ground truth annotations in order to benchmark these types of methods, and that this constitutes an important topic for the research community to develop and publish.

We here provide figure EV1 panels for the convenience of the reviewers.

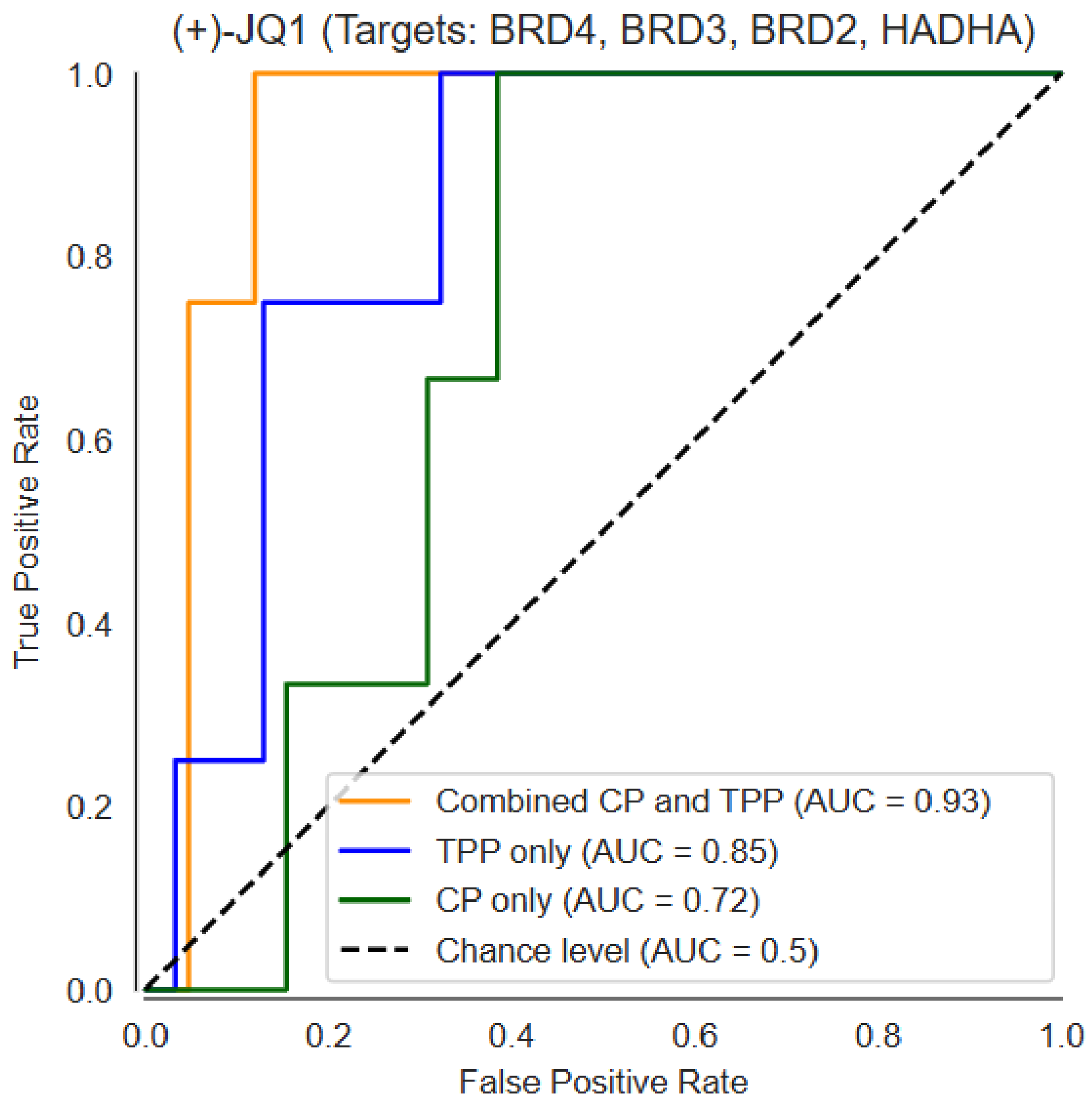
(+)-JQ1 (Targets: BRD4, BRD3, BRD2, HADHA)
1.0
0.8
0.6
0.4
0.2
0.0
True Positive Rate
0.0
0.2
0.4
0.6
0.8
1.0
False Positive Rate
Combined CP and TPP (AUC = 0.93)
TPP only (AUC = 0.85)
CP only (AUC = 0.72)
Chance level (AUC = 0.5)

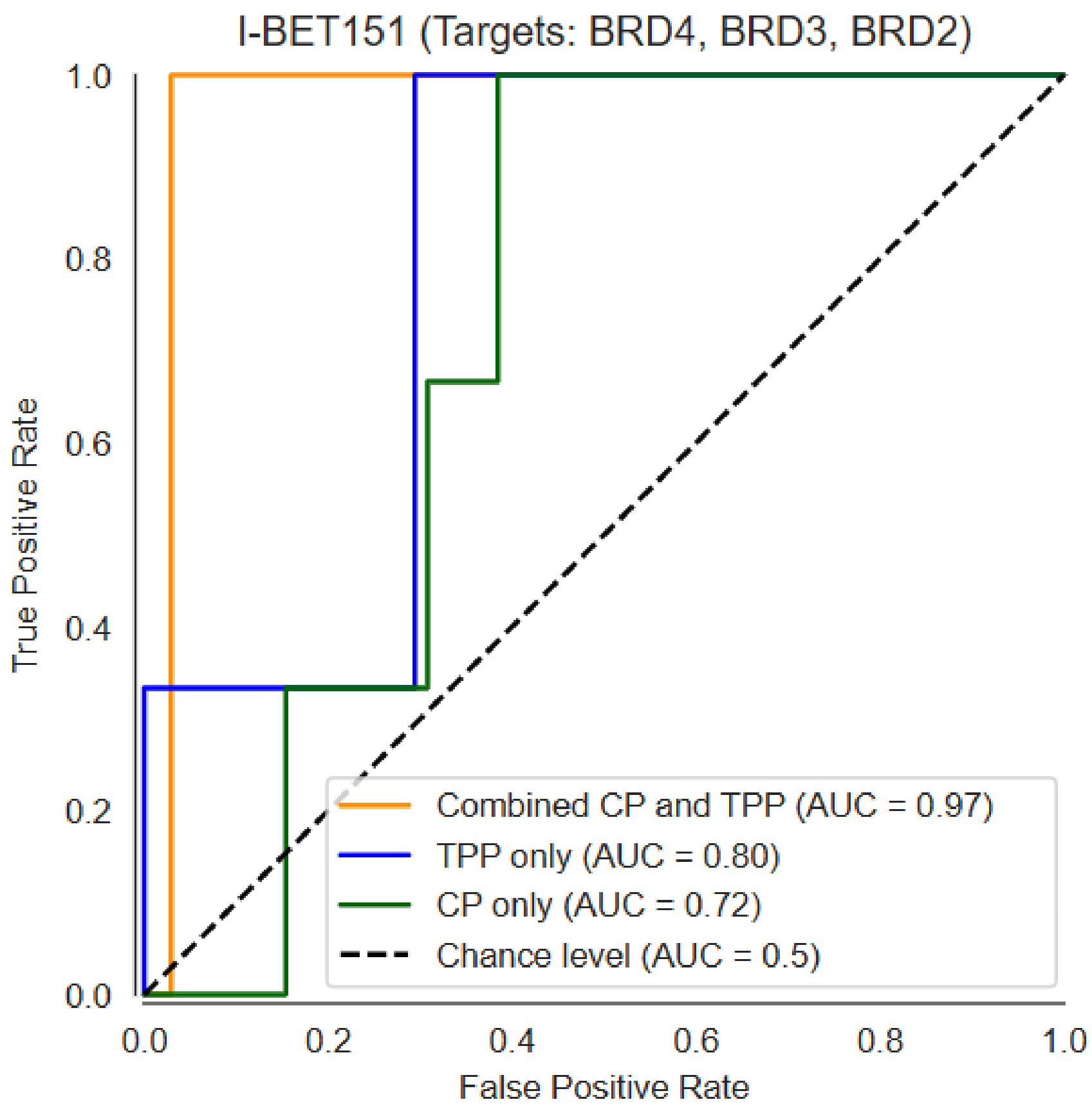
I-BET151 (Targets: BRD4, BRD3, BRD2)
True Positive Rate
False Positive Rate
0.0
0.2
0.4
0.6
0.8
1.0
Combined CP and TPP (AUC = 0.97)
TPP only (AUC = 0.80)
CP only (AUC = 0.72)
Chance level (AUC = 0.5)

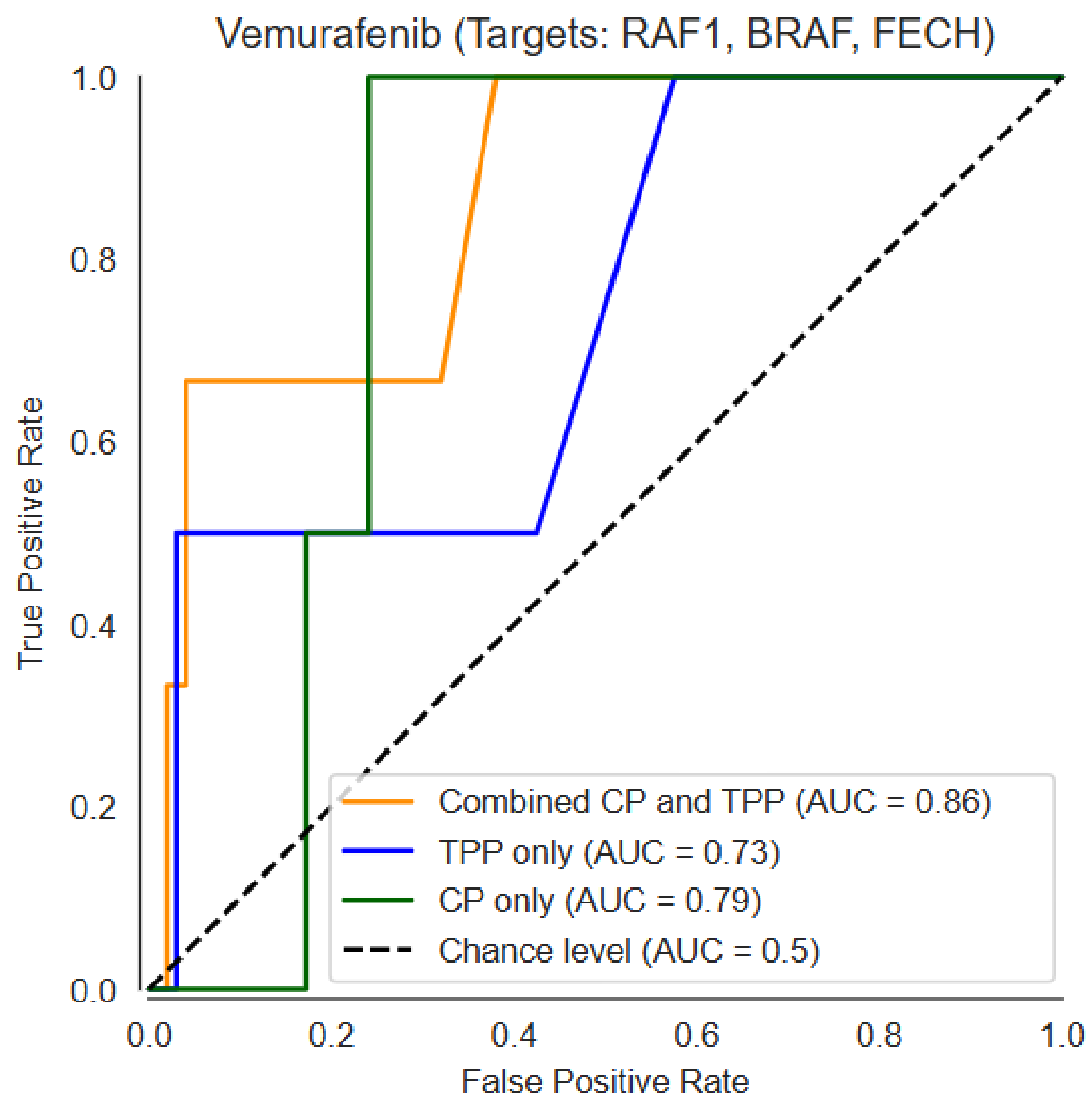
Vemurafenib (Targets: RAF1, BRAF, FECH)
True Positive Rate
False Positive Rate
0.0
0.2
0.4
0.6
0.8
1.0
Combined CP and TPP (AUC = 0.86)
TPP only (AUC = 0.73)
CP only (AUC = 0.79)
Chance level (AUC = 0.5)

- A thorough benchmarking and ablation analysis is critical for the manuscript to meet the standards of this journal. It should be clearly demonstrated how each component-TPP data, Cell Painting, network knowledge, expression contextualization-contributes to the overall prediction quality. For example, the authors should explore what happens to performance when using only TPP data or only clustering from Cell Painting. Similarly, the effect of network contextualization should be tested by comparing STRING-based networks to data-driven alternatives such as cross-linking networks or tissue-specific correlation-based networks (e.g., from https://www.nature.com/articles/s41587-025-02659-z). Furthermore, alternative network ranking methods such as network propagation or Steiner tree algorithms should be evaluated. All of these analyses should be supported by accuracy metrics, providing a clear picture of which components are essential and which may be redundant or detrimental.

Answer: We thank the reviewer for their suggestions of improvements and have introduced comparisons through an ablation analysis between TPP, CP and combined CP-TPP target ranking performance through ROC analysis. We have now added additional figures to highlight the performance of TPP alone, CP alone, and combined CP-TPP datasets (figure EV1). We also thank the reviewer for providing suggestions on other network analysis approaches one could benchmark our data against, however, the suggested article contained hardcoded analysis scripts and no ready-to use notebooks for our own data. Also, the further suggestions of networking ranking methods could be interesting to compare in future studies, however we regard this to be beyond the scope of the current manuscript.

In summary, while the manuscript presents an exciting and timely approach to MoA prediction, it requires substantial revisions. The novelty claims should be adjusted to reflect prior work, the structure should be reorganized for clarity, the methodology should be better explained and visualized, and quantitative evaluation and benchmarking need to be systematically introduced. With these improvements, the manuscript could be a strong candidate for publication.

Answer: We thank the reviewer for their encouraging comments and hope that we with our answers and revised manuscript have addressed all their concerns.

Reviewer #3:

Johansson et al. present a novel graph-based approach that integrates Cell Painting and Thermal Proteome Profiling data to better identify drug targets. The methodological innovation is both timely (given the growing interest in multimodal profiling) and compelling. The manuscript is generally well written and well organized, though several sections would benefit from improved clarity and additional explanation. I have a few major and mostly minor comments that primarily pertain to enhancing the readability of the work.

Major comments

- The integration strategy between the two data modalities is not sufficiently described. In particular, the manuscript introduces the combined network without explaining how the Cell Painting features are incorporated into the network construction. Moreover, the mathematical or algorithmic details of the integration procedure are not described in the Methods section. Given that the integration method represents the main contribution of the work, it is essential that this process be described in a reproducible and transparent manner. I recommend expanding both the Results and Methods sections to include a clear explanation of how the datasets were aligned, how features were combined, and what graph-based algorithms or similarity metrics were applied. Figure 1 presents a high-level understanding, but a deeper dive is needed for this manuscript.

Answer: We thank the reviewer for this feedback. The methods section has been re-organized and expanded to contain a step-by-step description of the integration workflow, please see pages 11–15 and in particular, an enumerated description of the CP-TPP data integration on page 15. Furthermore, Figure 1 has been updated for clarity.

- The statement that "the choice of community detection algorithm has a large impact on what potential mechanisms of action (MoAs) are identified in this step" is concerning. High sensitivity to the choice of algorithm raises questions about the robustness and reproducibility of the findings. It would be helpful if the authors could quantify this sensitivity (e.g., how much do the identified communities and MoA candidates vary across different algorithms?) Additionally, the potential for "cherry picking" results should be addressed explicitly, perhaps by discussing how algorithm choice was made and whether multiple algorithms were systematically compared. This would also strengthen confidence in the cited literature supporting their approach.

Answer: We agree with the reviewer that this phrasing causes unintentional concern. We have revised this paragraph in the results section and now write:

"The final PPI network in Figure 3C–D was further classified into five communities using the Clauset-Newman-Moore greedy modularity maximization [25] (Figure 4A). We additionally evaluated the Girvan-Newman, edge betweenness partition, and k-clique communities algorithm, which showed that either communities with only one single member were generated (Girvan-Newman and edge betweenness partition), or nodes were not associated with a community at all (k-clique communities). Hence, we finally chose the Clauset-Newman-Moore greedy modularity maximization algorithm that did not require a prior assumption of the number of, or size of, generated communities. This algorithm was able to generate communities with at least two members and did not fail to associate any nodes with a community. Figure EV2 shows a comparison of how these evaluated algorithms perform. "

Hence, we have removed the sentence "It should be noted that the choice of community detection algorithm has a large impact on what potential MoAs are identified in this step."

We believe this should provide a more clear description of our choice of clustering algorithm.

Minor comments

- Throughout the manuscript, the authors refer to "Cell Painting features," but this term is

ambiguous. There are different methods to derive features from Cell Painting data, and it is not immediately clear which method was used. Only in the methods section is it clarified that the features were extracted using CellProfiler. I recommend briefly specifying this earlier in the main text to improve clarity for readers less familiar with the technical details of image-based profiling.

Answer: We thank the reviewer for raising the need for this clarification. In the introduction we state "The images of stained cells are processed through software (here we use CellProfiler [9]), where thousands of features are extracted at a single-cell level to describe the morphological perturbation of each treatment."

- The Results section would benefit from a more detailed and quantitative characterization of the specific contribution made by the Cell Painting data. While the metaphor of "filling gaps" is helpful, it should be supplemented with a clearer technical explanation of how CP features enhance or complement the TPP-derived network. For instance, are CP features used to infer new edges, refine existing relationships, or improve prioritization? Additionally, in the Discussion section, the authors state: "Here, we show that by combining CP and TPP data we were able to substantially narrow down the list of possible drug targets." However, the quantitative basis for this claim is not clearly presented in the Results section. For example, while it is mentioned in the Discussion that the known target appears within the top ten, this alone does not convincingly support the strength of the integration. It would be helpful to report the actual number of candidates considered before and after integration, and to provide similar metrics for CP alone. This would allow readers to assess the added value of combining modalities versus using TPP or CP data in isolation. Such an analysis would substantially strengthen the manuscript's core claim.

Answer: We thank the reviewer for these suggestions, which is also similar to concerns from reviewer 2 (please see our reply above). We have conducted an ablation analysis where results from TPP alone, CP alone and the combined CP-TPP dataset are compared (Figure EV1). We have added an enumerated explanation of the method (page 15). Further, in the results section we have generated tables showing results for BCS from CP alone, TPP alone and compare with the integration, please see new tables S4, S5, S12, S13, S15, S16, S17, S19, S20, S21, S23, S24, S25. We refer to these additional tables in the result sections for the five drugs we validated our computational pipeline with. Together with the ablation analysis (ROC curves, figure EV1), we show that integration of CP-TPP data results in known targets having generally higher BCS.

- While it may be beyond the current scope of the manuscript, it could be valuable to consider how the integration method might perform across different TPP protocols, given the variability in experimental design (e.g., number of temperature points, compound concentrations, or cell types used). At the very least, I recommend expanding the Discussion section to acknowledge these protocol differences and speculate on whether and how the method would need to be adapted. This would help clarify the generalizability of the approach and guide future applications across diverse experimental setups.

Answer: The reviewer is correct that many various TPP protocols exist. In addition, there are also several ways to infer thermal shifts from the data, including fitting sigmoid curves and calculating AUCs. Several fit criteria also exist, which ultimately impacts the final list of shifted proteins. However, our method only utilizes the information whether a protein has been shifted or not, and is therefore generally applicable to any form of TPP data.

- It would be helpful to introduce the known targets and pathways associated with JQ1 and I-BET151 earlier in the manuscript. These compounds are bromodomain inhibitors, but this important contextual information is not provided until later. Including this detail upon first mention (especially in the Results section) would improve clarity for readers who may not be familiar with these compounds and better contextualize the downstream analyses.

Answer: We thank the reviewer for this observation and have revised the beginning of the results section to clarify that JQ1 and I-BET151 are bromodomain inhibitors and which targets they are known to interact with.

"(+)-JQ1 and I-BET151 are both BET bromodomain protein inhibitors with shared targets [5]."

"A list of known targets and off-targets for (+)-JQ1 and I-BET151 was constructed from literature to allow quantification of the specificity and sensitivity of the proposed pipeline (Table 2). (+)-JQ1 targets BRD4 and BRDT, with documented off-target effects on BRD2, BRD3 and HADHA. I-BET151 targets BRD4, BRD3 and BRD2. "

- The following statement in the Results section is quite informative but may benefit from clearer presentation: "Preliminary clustering attempts for the CP data using k-means, HDBSCAN, and agglomerative clustering showed that the tightest clusters could be achieved using HDBSCAN with a prior principal component analysis (PCA) feature reduction, but this approach also resulted in the largest variation in cluster size and a high risk that the compound of interest would not be included in any cluster at all. k-means clustering on PCA data with between 100 and 300 k clusters performed similarly to agglomerative clustering using the same number of clusters as input parameters." This is an important methodological comparison and could be clarified and strengthened by including a dedicated figure that visually compares the performance of the different clustering approaches (e.g., cluster size distributions, number of unassigned compounds, or some clustering metric like silhouette score). Although Figure S1 is cited, it does not directly illustrate this analysis. A new supplemental or main figure focused on this comparison would help readers interpret and contextualize the choice of clustering strategy.

Answer: During initial pipeline development we noted that HDBSCAN had difficulty to deliver a consistent clustering. Upon reception of the reviewer's comments on this we agree that visualizing how the clustering algorithms behave is useful to the readers. Hence, we show now how different clustering behaves for Vemurafenib (new figure EV3), JQ1 and I-BET151 (new appendix figures S11, S12). When we evaluated the algorithms for these drugs we found that in contast to early attempts on a single drug, when we now evaluated HDBSCAN with Vemurafenib, JQ1 and I-BET151 we were repeatedly unable to cluster around the drugs at all, while individual runs of k-means clustering and agglomerative clustering delivered

consistent clusters with the size range of 15-25 drugs. The clustering behavior for the three different clustering algorithms are now showed in Figure EV3 and we have amended the prior text to reflect our findings.

“We considered that a suitable clustering algorithm for our purpose should be able to detect small (about 20-60 samples), tight clusters in CP data with high reproducibility. Preliminary clustering attempts for the CP data using k-means, HDBSCAN and agglomerative clustering showed that the tightest clusters could be achieved with using k-means and agglomerative clustering with a prior principal component analysis (PCA) feature reduction. In contrast, HDBSCAN was unable to cluster the drugs at all. Figure EV3 shows a comparison of how the three clustering algorithms behaved. k-means clustering on PCA data with between 100 and 300 k clusters performed similarly to agglomerative clustering using the same number of clusters as input parameters.“

- Several figures and tables would benefit from clarification to improve interpretability:

o Figure 2B: The text states that a cluster "behaves similarly," yet the blue points in the t-SNE plot appear widely distributed. It would be helpful if the authors clarified what "behaves similarly" means in this context, and how this is visually supported by the figure.

Answer: We agree that the phrasing is misleading, instead we now state “t-SNE plot of cell painting data that in color highlight the cluster that results from k-means clustering and that include Sinomenine. ” We would like to emphasize that the clusters are generated with k-means clustering on PCA data and not with t-SNE. Hence, the dots in t-SNE space is not necessarily representing cluster similarities the same way as the multidimensional PCA plot.

o Figure 2C: The figure is very dense, and many protein names are difficult to read due to overlapping text and font size. In addition, the authors should clarify what the point sizes and line lengths represent.

Answer: We agree with the reviewer and have revised the figure. When networks get very large it is difficult to make every single node label readable, but we believe the revised figure is clearer. We now also explain in all network figures in the manuscript that the sizes of the nodes indicate whether the proteins have been found in TPP (large size) or CP (small size), and that edge thickness corresponds to the confidence score of the interaction. The length of the edges has no representation.

o Table 1: The meaning of the asterisk ("Added from CP cluster") is unclear. Does this mean these targets would not have been identified using TPP data alone? Would the table be identical without Cell Painting data, minus the asterisked entries? Clarifying whether the TPP-based rankings change after integration with CP would help readers understand the added value of the multimodal approach. Moreover, the section discussing Table 1 mentions these additional targets but does not elaborate on why Cell Painting, rather than TPP, identifies them. A brief discussion of whether this is expected (e.g., what biological properties CP captures that TPP may miss) would be helpful.

Answer: The reviewer has identified a diffuse statement that now is better explained in the methods sections (see page 15). We hope that the methodology and the ablation analysis

we now show comparing TPP alone, CP alone and the combination of CP and TPP (figure EV1), provide a clearer explanation of the pipeline. In the table footnote we now explicitly state that this protein was not present in TPP data. "*Not present in TPP dataset, added from computational pipeline"

The reviewer is correct that these highlighted proteins would not have been found in a TPP dataset alone, but are found after computation using both TPP and CP datasets. Since modern proteomics workflows based on LC–MS only cover half the proteome (10,000 out of 20,000 canonical proteins) we expect that there exist drug targets that are not currently detectable with TPP. We have extended the discussion with the following sentences:

"Complementing TPP data with other measurable modalities can be critical, because a typical LC--MS analysis can only cover half the proteome, with identification and quantitation of roughly 10,000 out of 20,000 canonical proteins. Hence, conducting TPP alone has a risk to miss true drug targets. We show that by increasing the accessible information of target engagement with CP, the number of hypothetical targets to test were effectively narrowed down to ten or fewer proteins, compared to the between 35--64 targets found for the investigated compounds using only TPP."

o Figure 3D: The quantification method used to generate this radar plot should be described more clearly. What is the measurement? It is unclear whether the volume formed by the lighter blue region has specific meaning, given that the spokes of a radar plot have no inherent ordering. Additionally, the meanings of "pos" and "neg" in this figure are not explained and should be defined either in the figure or accompanying legend.

Answer: We have improved the figure caption and now state " Radar chart showing mean z-scores for CellProfiler features in cell painting data for (+)-JQ1 and I-BET151. The blue and orange lines indicate positive or negative mean z-score values, respectively. Note that there is no intrinsic order and the shading is only providing visual clarity of the features. "

o Figure 4A: The color contrast in this panel is suboptimal. The various shades of blue used for different clusters are too similar to distinguish easily, and the dark orange point mentioned in the text is nearly invisible. Adjusting the color palette to improve accessibility and contrast would enhance readability.

Answer: We agree and want to clarify that (+)-JQ1 and I-BET151 appear in the same cluster. The figure legend has been updated to reflect this.

o Figure 4C-D: The sentence "Figure 4C-D show PPI networks for all identified TPP nodes combined with the six versus eight selected CP nodes" is difficult to follow. Please clarify whether the two panels represent different node combinations, different compound treatments, or different analysis strategies.
Answer: We clarify in the results what the six and eight nodes are with the following updated description

"The targets identified in the cluster were then combined with the affected proteins from TPP data and a new PPI network was constructed. Since this network became very large, the betweenness centrality scores for the CP cluster targets were recalculated and sorted. The

proteins with the lowest BCS from the CP cluster were iteratively removed as long as the total number of TPP nodes included in the network was not reduced. For (+)-JQ1, six nodes from the CP cluster were kept (LRRK2, MMP14, MMP9, ROCK1, PPARA and LUC7L3). For I-BET151, eight nodes fulfilled the same criteria (LRRK2, MMP9, ROCK1, MTOR, ROCK2, CDKN1A, DMP1 and HARS1). Figure 3C–D show PPI networks for all identified TPP nodes combined with the six ([+]-JQ1) versus eight (I-BET151) selected CP nodes"

- The gene ontology and Reactome enrichment analysis in Figure 5B is compelling, particularly the proposed mechanism whereby (+)-JQ1 acts through lysine-acetylated histone binding, potentially leading to cell death and alterations in lipid metabolism. However, the manuscript does not clearly explain how the enrichment analysis supports the inferred ordering of pathway activation. It would strengthen the interpretation to clarify whether these results suggest a causal sequence or if this is just conjecture.

Answer: We thank the reviewer for this observation. The proposed method does not propose ordering of pathway activation. We have revised the sentence which now reads " Gene Onthology (GO) and Reactome enrichment analysis (Figure 4B) suggested that (+)-JQ1 possibly through mechanisms like lysine-acetylated histone binding (Community 3), activation of cell death (Community 4) or affecting lipid metabolism (Community 2)."

- The authors attribute the reproducibility of Vemurafenib to the fact that its morphological profile did not fall within visually separated clusters (Figure S4E). However, upon inspection, Figure S4E appears to show distributions similar to those of other compounds highlighted in Figure S4D, Figure 4A, and Figure 3E. This raises some uncertainty about the basis for this explanation. It would be helpful if the authors could clarify what distinguishes Vemurafenib's profile in these visualizations and provide additional evidence supporting its unique reproducibility characteristics.

Answer: We thank the reviewer for this comment. In the manuscript, we referred to the fact that there are more compounds in close proximity to JQ1/I-BET151, Crizotinib and Panibonistat in the UMAP space that share targets with these compounds than there are for Vemurafenib. However, as this is not clearly visible in the figures since the close data points are often "hidden" underneath the points for JQ1/I-BET151, Crizotinib and Panibonistat in the figures due to figure/marker size. Since the statement was not supported by any additional quantitative measure, we have removed the statement from the manuscript to avoid confusion.

- Table 4 presents a key result showing whether the known target is present in the identified cluster. However, simply stating "Yes, the target is in the cluster" may not be a sufficiently informative metric. For instance, if most or all compounds appear in the clusters, this metric lacks specificity and may overstate performance. It would be valuable to include measures of sensitivity, specificity, or other relevant classification metrics to better assess the accuracy and robustness of target identification. Additionally, the phrase "model predicts target or mechanism of action (MOA)" is used without a clear definition of what the model entails or how predictions are generated. Clarifying the nature of this model and the prediction methodology would improve transparency and help readers interpret these results appropriately.

Answer: We thank the reviewer for the suggestions and have added a ROC analysis to compare target identification between TPP only, CP only and CP-TPP combined (figure EV1) as well as updated the results and discussion. Defining an accurate “ground truth” for MOA from literature is highly challenging and prone to either result in an incomplete list of MOAs or lists overpopulated with false positives. We have therefore opted to tone down the discussion around MoA predictions in the manuscript and have added a discussion about the challenges of validating MoA predictions.

EDITORIAL REQUIREMENTS

All Materials and Methods need to be described in the main text using our 'Structured Methods' format

On a more editorial level, we would ask you to address the following issues:

- Please provide a .docx formatted version of the manuscript text (including legends for main figures, EV figures and tables). Please make sure that the changes are highlighted to be clearly visible.

- Please provide individual production quality figure files as .eps, .tif, .jpg (one file per figure).

-Please provide a .docx formatted letter INCLUDING the reviewers' reports and your detailed point-by-point responses to their comments. As part of the EMBO Press transparent editorial process, the point-by-point response is part of the Review Process File (RPF), which will be published alongside your paper.

-Please note that all corresponding authors are required to supply an ORCID ID for their name upon submission of a revised manuscript.

-We replaced Supplementary Information with Expanded View (EV) Figures and Tables that are collapsible/expandable online (see examples inhttp://msb.embopress.org/content/11/6/812). A maximum of 5 EV Figures can be typeset. EV Figures should be cited as 'Figure EV1, Figure EV2" etc... in the text and their respective legends should be included in the main text after the legends of regular figures.

Additional Tables/Datasets should be labeled and referred to as Table EV1, Dataset EV1, etc. Legends have to be provided in a separate tab in case of .xls files. Alternatively, the legend can be supplied as a separate text file (README) and zipped together with the Table/Dataset file.
For the figures and tables that you do NOT wish to display as Expanded View figures, they should be bundled together with their legends in a single PDF file called *Appendix*, which should start with a short Table of Content. Each legend should be below the corresponding Figure/Table in the Appendix. Appendix figures and tables should be referred to in the main text as: "Appendix Figure S1, Appendix Figure S2, Appendix Table S1" etc. See detailed instructions regarding expanded view here:https://www.embopress.org/page/journal/17444292/authorguide#expandedview.

-Before submitting your revision, primary datasets (and computer code, where appropriate) produced in this study need to be deposited in an appropriate public database (seehttp://msb.embopress.org/authorguide - dataavailability https://www.embopress.org/page/journal/17444292/authorguide#dataavailability).

Please remember to provide a reviewer password if the datasets are not yet public.

The accession numbers and database should be listed in a formal "Data Availability" section (placed after Materials & Method) that follows the model below (see alsohttps://www.embopress.org/page/journal/17444292/authorguide#dataavailability). Please note that the Data Availability Section is restricted to new primary data that are part of this study.

Data availability

The datasets (and computer code) produced in this study are available in the following databases:

- RNA-Seq data: Gene Expression Omnibus GSE46843 (https://www.ncbi.nlm.nih.gov/geo/query/acc.cgi?acc=GSE46843)
- [data type]: [name of the resource] [accession number/identifier/doi] ([URL or identifiers.org/DATABASE:ACCESSION])

*** Note - All links should resolve to a page where the data can be accessed. ***

- At EMBO Press we ask authors to provide source data for the main manuscript figures. You will receive a separate email with instructions for providing source data with your revised manuscript, including how to upload and organize the files.

Additional information on source data and instruction on how to label the files are available https://www.embopress.org/page/journal/14693178/authorguide#sourcedata

- Our journal encourages inclusion of *data citations in the reference list* to directly cite datasets that were re-used and obtained from public databases. Data citations in the article text are distinct from normal bibliographical citations and should directly link to the database records from which the data can be accessed. In the main text, data citations are formatted as follows: "Data ref: Smith et al, 2001". In the Reference list, data citations must be labeled with "[DATASET]". A data reference must provide the database name, accession number/identifiers and a resolvable link to the landing page from which the data can be accessed at the end of the reference. Further instructions are available at https://www.embopress.org/page/journal/17444292/authorguide#referencesformat >.

- We updated our journal's competing interests policy in January 2022 and request authors to consider both actual and perceived competing interests. Please review the policy https://www.embopress.org/competing-interests and update your competing interests if necessary.

Please use the heading "Disclosure statement and competing interests".

- All Materials and Methods need to be described in the main text using our 'Structured Methods' format. According to this format, the Methods section includes a Reagents and Tools Table (listing key reagents, experimental models, software and relevant equipment and including their sources and relevant identifiers) followed by a Methods and Protocols section describing the methods, ideally using a step-by-step protocol format. The aim is to facilitate adoption of the methodologies across labs.

Please download and fill our Reagents and Tools Table template (.docx), which you can find in our author guidelines:https://www.embopress.org/page/journal/17444292/authorguide#structuredmethods.

When submitting your revised manuscript, please do not include the Reagents and Tools Table in the Methods section of the manuscript but upload it as a separate file choosing the file type "Reagent Table".

An example of a Method paper with Structured Methods can be found here: https://www.embopress.org/doi/10.15252/msb.20178071.

-Regarding data quantification:

Please ensure to specify the name of the statistical test used to generate error bars and P values, the number (n) of independent experiments (please specify technical or biological replicates) underlying each data point and the test used to calculate p-values in each figure legend. Discussion of statistical methodology can be reported in the materials and methods section, but figure legends should contain a basic description of n, P and the test applied.

Graphs must include a description of the bars and the error bars (s.d., s.e.m.).

Please also include scale bars in all microscopy images.

- Please provide a "standfirst text" summarizing the study in one or two sentences (approximately 250 characters, including space), three to four "bullet points" highlighting the main findings and a "synopsis image" (550px width and 400-600 px height, PNG format) to highlight the paper on our homepage.

Here are a couple of examples:

https://www.embopress.org/doi/10.15252/msb.20199356

https://www.embopress.org/doi/10.15252/msb.20209475

https://www.embopress.org/doi/10.15252/msb.209495

When you resubmit your manuscript, please download our CHECKLIST (https://www.embopress.org/pb-assets/embo-site/EMBO%20Press%20Author%20Checklist-1642513524327.xlsx) and include the completed form in your submission.

Please note that the Author Checklist will be published alongside the paper as part of the transparent process (https://www.embopress.org/page/journal/17444292/authorguide#transparentprocess).

18th Dec 2025

Manuscript Number: MSB-2025-13221R
Title: Cell Painting and Thermal Proteome Profiling for Inference of Drug Targets and Mechanism of Action
Author: Camilla Johansson
Martin Johansson
Phanindra Kasi
Mårten Larsson
Per-Johan Jakobsson
Ulf Göransson
Jordi Carreras Puigvert
Ola Spjuth
Erik Jansson

Dear Dr. Jansson,

Thank you again for submitting the revised version of your manuscript. We have now received feedback from both reviewers who evaluated your revision. As you will see below, while Reviewer #3 is overall satisfied with the changes made, Reviewer #2 notes that an important issue raised in the previous round of review -regarding 'systematic validation'- remains insufficiently addressed.

In principle, our editorial policy permits only a single round of major revision. However, given that the reviewer has provided constructive suggestions, we are willing to allow you to address this issue in an exceptional second round of major revision. Please note that this will be the final revision round, so it is essential that all remaining concerns are fully addressed.

Please also address the remaining minor issues raised by Reviewer #3.

On a more editorial level:

1. Remove all figures from the manuscript file and upload them as individual files. Place the figure legends below the "References" section.

2. Please ensure that all figure callouts are listed sequentially and add the missing callout for Figure 3D.

3. Please also make sure that all funding information mentioned in the manuscript text is entered in the submission system.

4. Update all source file names, titles, legends, and manuscript callouts to Dataset EV1-EV4 instead of Appendix file 1-4. Legends should be uploaded as a separate tab/sheet within each Excel file.

5. Please provide a "standfirst text" summarizing the study in one or two sentences (approximately 250 characters, including space), three to four "bullet points" highlighting the main findings.

6. The references need to be formatted according to the Molecular Systems Biology reference style. Please list up to 10 co-authors of a paper before adding et al. in the reference list. Citations should be listed in alphabetical order.

7. "Materials and Methods" should be renamed to "Methods".

8. The section "Supplementary information. Supplementary material (PDFdocument)." should be removed from manuscript file.

9. "Data availability": Please note that this section should include only primary data and code generated by the current study. All previously published datasets should be removed from this section. Please provide the specific URL for PXD065743, remove the reviewer access code, and ensure that the dataset will be made publicly available upon acceptance of the manuscript.

10. Please address the following issues in figure legends:

- Please indicate the statistical test used for data analysis in the legend of figure 5A2. Please note that information related to n is missing in the legend of figure 5A.

- Please note that information related to n is missing in the legend of figure 5A.

11. Please upload Appendix (currently named as Supplementary file) in a single pdf file. The Appendix file should begin with a

title page ("Appendix for [manuscript title]"), followed by a Table of Contents that includes page numbers for all listed items. Please also make sure all the Appendix Tables are called out.

12. Sections need to be named and the order should be corrected to : Title page - Abstract - Keywords - Introduction - Results - Discussion - Methods - Data Availability - Acknowledgements - Disclosure and Competing Interests Statement - References - Figure Legends - Table(s) - Expanded View Figure Legends.

If you feel you can satisfactorily deal with these points and those listed by the referees, you may wish to submit a revised version of your manuscript. Please attach a covering letter giving details of the way in which you have handled each of the points raised by the referees. A revised manuscript will be once again subject to review and you probably understand that we can give you no guarantee at this stage that the eventual outcome will be favorable.

We look forward to receiving your revised manuscript soon.

Kind regards,
Jingyi

Jingyi Hou, PhD
Senior Editor
Molecular Systems Biology

We realize that it is difficult to revise to a specific deadline. In the interest of protecting the conceptual advance provided by the work, we recommend a revision within 3 months (18th Mar 2026). Please discuss the revision progress ahead of this time with the editor if you require more time to complete the revisions.

IMPORTANT: Please read our guidelines for revised submissions: https://link.springer.com/journal/44320/submission-guidelines#cms-Revised-submissions

*** PLEASE NOTE *** As part of the EMBO Press transparent editorial process initiative (see our Editorial at https://dx.doi.org/10.1038/msb.2010.72), Molecular Systems Biology publishes online a Review Process File with each accepted manuscripts. This file will be published in conjunction with your paper and will include the anonymous referee reports, your point-by-point response and all pertinent correspondence relating to the manuscript. If you do NOT want this File to be published, please inform the editorial office at contact@molsystbiol.org within 14 days upon receipt of the present letter.

Reviewer #2:

The authors have addressed most of my comments; however, the most critical one, the systematic validation of the added value of cell painting data to their methodology, remains unresolved and insufficiently addressed. While I understand their decision not to explore the alternative methodological approaches suggested in the first review, I would recommend at minimum supplementing their benchmark with publicly available data. As they themselves note, using only the five compounds in the paper is not sufficient for a systematic validation.

A concrete option would be to leverage the TPP atlas published here: https://elifesciences.org/articles/95595, and integrate it with a public cell painting dataset that includes at least 20 to 30 kinase inhibitors. For these inhibitors, primary targets (and thus the proteins that should appear in the MoA prediction) can be extracted from the kinobeads data and treated as true positives. In my view, this additional systematic analysis is necessary to demonstrate that the ablation study results are robust.

Reviewer #3:

The authors have adequately addressed my concerns, and the manuscript is suitable for publication.

One minor, easy fix is to read the manuscript carefully for necessary copy-editing as there are some grammar mistakes and incomplete sentences. For example, one of the updated edits from the first review introduced the following incomplete sentence: "Gene Onthology (GO) and Reactome enrichment
analysis (Figure 4B) suggested that (+)-JQ1 possibly through mechanisms like lysine-acetylated histone binding (Community 3),

activation of cell death (Community 4) or affecting lipid metabolism (Community 2)."

Reviewer #2:

The authors have addressed most of my comments; however, the most critical one, the systematic validation of the added value of cell painting data to their methodology, remains unresolved and insufficiently addressed. While I understand their decision not to explore the alternative methodological approaches suggested in the first review, I would recommend at minimum supplementing their benchmark with publicly available data. As they themselves note, using only the five compounds in the paper is not sufficient for a systematic validation.

A concrete option would be to leverage the TPP atlas published here: https://elifesciences.org/articles/95595, and integrate it with a public cell painting dataset that includes at least 20 to 30 kinase inhibitors. For these inhibitors, primary targets (and thus the proteins that should appear in the MoA prediction) can be extracted from the kinobeads data and treated as true positives. In my view, this additional systematic analysis is necessary to demonstrate that the ablation study results are robust.

We thank the reviewer for this valuable suggestion to improve our manuscript and we have now analysed 49 compounds from the suggested PISA atlas using our pipeline. The new results are added as Figure S13-S18 and to the Results section under the subheading "Validation of target prediction using 49 compounds".

The abstract has been re-written to mention the results from the 49 compounds while keeping within the 175 word limit.

The Discussion has been reordered and edited on paragraph 2 and 7 to include discussion around the added data analysis

Reviewer #3:

The authors have adequately addressed my concerns, and the manuscript is suitable for publication.

We thank the reviewer for this approval and the previous comment which helped us improve the manuscript.

One minor, easy fix is to read the manuscript carefully for necessary copy-editing as there are some grammar mistakes and incomplete sentences. For example, one of the updated edits from the first review introduced the following incomplete sentence: "Gene Onthology (GO) and Reactome enrichment

analysis (Figure 4B) suggested that (+)-JQ1 possibly through mechanisms like lysine-acetylated histone binding (Community 3), activation of cell death (Community 4) or affecting lipid metabolism (Community 2)."

The manuscript has now been revised for grammatical errors, incomplete sentences and spelling errors.

Other edits

Figure EV3, as well as Appendix figure S11 and S12 have been edited to have text font, text size and line thickness adhere to journal standard. Vectorized UMAP plots with high density data have been rasterized to reduce file size while maintaining readability.

The abstract has been re-written to contain the added data analysis while staying within the 175 word limit.

We unfortunately discovered an error with the Sinomenine PISA data where the MS2 spectra had accidentally been searched with a tolerance of 20 ppm instead of 0.5 Da (as the experiment had been run on a different MS instrument than we regularly use). The data has now been re-searched and re-analysed using our pipeline. Figure 5A, Figure 5C, Table 4 and Table 5 have been updated with the changes to the data, along with Dataset EV1 (formerly called Appendix data 1 and 2). Former Appendix data 3 and 4 have been added as source data to figure 5C. The results and discussion have been updated to reflect these new results. This change slightly decreased the number of identified thermal shifts from 179 to 175 for 10 uM sinomenine and 131 to 129 for 30 uM sinomenine. While the appearance of the final PPI network, the calculated betweenness centrality scores and order of identified GO-terms have been updated, the overall conclusions for Sinomenine remain largely the same.

We deeply apologize for this additional change to the manuscript.

Further, we have addressed all the editorial requests below:

1. Remove all figures from the manuscript file and upload them as individual files. Place the figure legends below the "References" section.

   2. Please ensure that all figure callouts are listed sequentially and add the missing callout for Figure 3D.

   3. Please also make sure that all funding information mentioned in the manuscript text is entered in the submission system.

   4. Update all source file names, titles, legends, and manuscript callouts to Dataset EV1-EV4 instead of Appendix file 1-4. Legends should be uploaded as a separate tab/sheet within each Excel file.

5. Please provide a "standfirst text" summarizing the study in one or two sentences (approximately 250 characters, including space), three to four "bullet points" highlighting the main findings.

6. The references need to be formatted according to the Molecular Systems Biology reference style. Please list up to 10 co-authors of a paper before adding et al. in the reference list. Citations should be listed in alphabetical order.

7. "Materials and Methods" should be renamed to "Methods".

8. The section "Supplementary information. Supplementary material (PDFdocument)." should be removed from manuscript file.

9. "Data availability": Please note that this section should include only primary data and code generated by the current study. All previously published datasets should be removed from this section. Please provide the specific URL for PXD065743, remove the reviewer access code, and ensure that the dataset will be made publicly available upon acceptance of the manuscript.

10. Please address the following issues in figure legends:

- Please indicate the statistical test used for data analysis in the legend of figure 5A2. Please note that information related to n is missing in the legend of figure 5A.

- Please note that information related to n is missing in the legend of figure 5A.

11. Please upload Appendix (currently named as Supplementary file) in a single pdf file. The Appendix file should begin with a title page ("Appendix for [manuscript title]"), followed by a Table of Contents that includes page numbers for all listed items. Please also make sure all the Appendix Tables are called out.

12. Sections need to be named and the order should be corrected to : Title page - Abstract - Keywords - Introduction - Results - Discussion - Methods - Data Availability - Acknowledgements - Disclosure and Competing Interests Statement - References - Figure Legends - Table(s) - Expanded View Figure Legends.

We thank again the editor and reviewers for their constructive feedback and comments. We hope that our manuscript is now well fitted for publication in MSB.

16th Apr 2026

Manuscript Number: MSB-2025-13221RR
Title: Cell Painting and Thermal Proteome Profiling for Inference of Drug Targets and Mechanism of Action
Author: Camilla Johansson
Martin Johansson
Phanindra Kasi
Mårten Larsson
Per-Johan Jakobsson
Ulf Göransson
Jordi Carreras Puigvert
Ola Spjuth
Erik Jansson

Dear Dr. Jansson,

Thank you for submitting the revised version of your manuscript. We have now received feedback from the reviewer who was invited to re-evaluate your study. As you will see from the comments below, the reviewer is generally satisfied with the revisions you have made, while still raising several minor issues.

Before we can formally accept your manuscript, please address these reviewer comments carefully, as well as the following editorial issues:

1. Funding information:The comments box in the submission system should not be used for funding details. Instead, these funders must be entered as separate entries. Please ensure that the following funders are listed in the system: Chemical Biology Consortium Sweden (CBCS), the node at Uppsala University. CBCS is a national research infrastructure funded by the Swedish Research Council (2021-00179) and SciLifeLab.

2. Please provide a "standfirst" text in a separate .docx file summarizing the study in one or two sentences (approximately 250 characters, including spaces), along with three to four bullet points highlighting the main findings.

3. Please clearly indicate corresponding author on the cover page of your manuscript and include email address.

4. Appendix:
- Please update the nomenclature and callouts to "Appendix Figure Sx" and "Appendix Table Sx."
- Please remove the author list and affiliations from the cover page.

5. When listing legends for EV figures, please use the format "Figure EV1: legend" rather than "EV1: legend."

6. Please remove the "Reagents and Tools" table title from the manuscript file.

As a matter of course, please make sure that you have correctly followed the instructions for authors as given on the submission website.

Thank you for submitting this paper to Molecular Systems Biology.

Kind regards,
Jingyi

Jingyi Hou, PhD
Senior Editor
Molecular Systems Biology

IMPORTANT: Please read our guidelines for revised submissions: https://link.springer.com/journal/44320/submission-

guidelines#cms-Revised-submissions

*** PLEASE NOTE *** As part of the EMBO Press transparent editorial process initiative (see our Editorial at https://dx.doi.org/10.1038/msb.2010.72 , Molecular Systems Biology will publish online a Review Process File to accompany accepted manuscripts. When preparing your letter of response, please be aware that in the event of acceptance, your cover letter/point-by-point document will be included as part of this File, which will be available to the scientific community. More information about this initiative is available in our Instructions to Authors. If you have any questions about this initiative, please contact the editorial office (msb@embo.org).

Reviewer #2:

I thank the authors for preprocessing and analyzing a larger dataset (Van Vranken et al., 2024) in response to the point I raised previously. This addition strengthens the paper. I have two minor comments. First, it would be helpful to complement the authors' overview of the results with a clear summary sentence stating, out of the 49 drugs tested, in how many cases the combined approach outperformed CP alone and PISA alone. Second, I do not find the empty ROC curves in Supplementary Figure S15K, Figure S16C, E, F, I, and K, and Figure S17H and L informative. Their inclusion gives the impression that the figures were not carefully checked prior to submission.

Dear Dr. Hou,

Thank you for your positive response and for forwarding the reviewer's comments. We are pleased to hear that the reviewer is generally satisfied with the revisions.

Reviewer #2

I thank the authors for preprocessing and analyzing a larger dataset (Van Vranken et al., 2024) in response to the point I raised previously. This addition strengthens the paper. I have two minor comments.

First, it would be helpful to complement the authors' overview of the results with a clear summary sentence stating, out of the 49 drugs tested, in how many cases the combined approach outperformed CP alone and PISA alone.

Answer: We agree with the reviewer's suggestion and have added a statement on page 7.

Second, I do not find the empty ROC curves in Supplementary Figure S15K, Figure S16C, E, F, I, and K, and Figure S17H and L informative. Their inclusion gives the impression that the figures were not carefully checked prior to submission.

Answer: We thank the reviewer for observing this. The Appendix Figures S15, S16 and S17 have been updated so that the empty ROC curves are now removed. Since the total number of subfigures are now reduced from 49 to 40, Appendix Figure S18 (which before contained the 49th compound) is no longer needed and has been removed.

Editorial comments

We have carefully addressed all editorial points raised in your letter and outline our actions below:

1. Funding information: We have updated the submission system to include the required funders as separate entries: Chemical Biology Consortium Sweden (CBCS), the node at Uppsala University, funded by the Swedish Research Council (grant 2021-00179) and SciLifeLab.

2. Standfirst text: A separate .docx file containing a one-to-two sentence summary (within the ~250-character limit) and three to four bullet points highlighting the main findings is included with this submission.

3. Corresponding author: The corresponding author is now clearly indicated on the cover page of the manuscript, including their email address.

4. Appendix:

- Nomenclature and callouts have been updated throughout to "Appendix Figure Sx" and "Appendix Table Sx" as requested.

- The author list and affiliations have been removed from the appendix cover page.

5. EV figure legends: All legends for Extended View figures have been updated to follow the format "Figure EV1: legend."

6. Reagents and Tools table: The "Reagents and Tools" table title has been removed from the manuscript file.

We have also carefully reviewed and followed the journal's instructions for authors. The revised manuscript has been submitted via the link provided.

We hope the manuscript is now ready for formal acceptance and look forward to your response.

Kind regards,

Erik Jansson

21st Apr 2026
Manuscript number: MSB-2025-13221RRR
Title: Cell Painting and Thermal Proteome Profiling for Inference of Drug Targets and Mechanism of Action

Dear Dr. Jansson,

Thank you again for sending us your revised manuscript. We are now satisfied with the modifications made and I am pleased to inform you that your paper has been accepted for publication.

Your manuscript will be processed for publication by EMBO Press. It will be copy edited and you will receive page proofs prior to publication. Please note that you will be contacted by Springer Nature Author Services to complete licensing and payment information.

You may qualify for financial assistance for your publication charges - either via a Springer Nature fully open access agreement or an EMBO initiative. Check your eligibility: https://link.springer.com/journal/44320/how-to-publish-with-us

Should you be planning a Press Release on your article, please get in contact with embo_production@springernature.com as early as possible in order to coordinate publication and release dates.

If you have any questions, please do not hesitate to contact the Editorial Office. Thank you for your contribution to Molecular Systems Biology.

Sincerely,
Jingyi

Jingyi Hou, PhD
Senior Editor
Molecular Systems Biology

>>> Please note that it is Molecular Systems Biology policy for the transcript of the editorial process (containing referee reports and your response letter) to be published as an online supplement to each paper. If you do NOT want this, you will need to inform the Editorial Office via email immediately. More information is available here: https://link.springer.com/partners/embo-press/editorial-policies#Peer%20review

EMBO Press Author Checklist

Corresponding Author Name: Erik Jansson	
Journal Submitted to: Molecular Systems Biology	
Manuscript Number: MSB-2025-13221	

USEFUL LINKS FOR COMPLETING THIS FORM
The EMBO Journal - Author Guidelines
EMBO Reports - Author Guidelines
Molecular Systems Biology - Author Guidelines
EMBO Molecular Medicine - Author Guidelines

Reporting Checklist for Life Science Articles (updated January
This checklist is adapted from Materials Design Analysis Reporting (MDAR) Checklist for Authors. MDAR establishes a minimum set of requirements in transparent reporting in the life sciences (see Statement of Task: 10.31222/osf.io/9sm4x). Please follow the journal's guidelines in preparing your manuscript.
Please note that a copy of this checklist will be published alongside your article.

Abridged guidelines for figures
1. Data
The data shown in figures should satisfy the following conditions:
- the data were obtained and processed according to the field's best practice and are presented to reflect the results of the experiments in an accurate and unbiased manner.
- ideally, figure panels should include only measurements that are directly comparable to each other and obtained with the same assay.
- plots include clearly labeled error bars for independent experiments and sample sizes. Unless justified, error bars should not be shown for technical
- if n<5, the individual data points from each experiment should be plotted. Any statistical test employed should be justified.
- Source Data should be included to report the data underlying figures according to the guidelines set out in the authorship guidelines on Data

2. Captions
Each figure caption should contain the following information, for each panel where they are relevant:
- a specification of the experimental system investigated (eg cell line, species name).
- the assay(s) and method(s) used to carry out the reported observations and measurements.
- an explicit mention of the biological and chemical entity(ies) that are being measured.
- an explicit mention of the biological and chemical entity(ies) that are altered/varied/perturbed in a controlled manner.
- the exact sample size (n) for each experimental group/condition, given as a number, not a range;
- a description of the sample collection allowing the reader to understand whether the samples represent technical or biological replicates (including how many animals, litters, cultures, etc.).
- a statement of how many times the experiment shown was independently replicated in the laboratory.
- definitions of statistical methods and measures:
  - common tests, such as t-test (please specify whether paired vs. unpaired), simple χ2 tests, Wilcoxon and Mann-Whitney tests, can be unambiguously identified by name only, but more complex techniques should be described in the methods section;
  - are tests one-sided or two-sided?
  - are there adjustments for multiple comparisons?
  - exact statistical test results, e.g., P values = x but not P values < x;
  - definition of 'center values' as median or average;
  - definition of error bars as s.d. or s.e.m.

Please complete ALL of the questions below.
Select "Not Applicable" only when the requested information is not relevant for your study.

Materials

Newly Created Materials	Information included in the manuscript?	In which section is the information available? (Reagents and Tools Table, Materials and Methods, Figures, Data Availability Section)
New materials and reagents need to be available; do any restrictions apply?	Not Applicable	

Antibodies	Information included in the manuscript?	In which section is the information available? (Reagents and Tools Table, Materials and Methods, Figures, Data Availability Section)
For **antibodies** provide the following information:<br>- Commercial antibodies: RRID (if possible) or supplier name, catalogue number and or/clone number<br>- Non-commercial: RRID or citation	Not Applicable	

DNA and RNA sequences	Information included in the manuscript?	In which section is the information available? (Reagents and Tools Table, Materials and Methods, Figures, Data Availability Section)
**Short novel DNA or RNA including primers, probes**: provide the sequences.	Not Applicable	

Cell materials	Information included in the manuscript?	In which section is the information available? (Reagents and Tools Table, Materials and Methods, Figures, Data Availability Section)
**Cell lines:** Provide species information, strain. Provide accession number in repository **OR** supplier name, catalog number, clone number, and/**OR** RRID.	Yes	Materials and methods page 12
**Primary cultures:** Provide species, strain, sex of origin, genetic modification status.	Not Applicable	
Report if the cell lines were recently **authenticated** (e.g., by STR profiling) and tested for mycoplasma contamination.	Yes	Materials and methods page 12

Experimental animals	Information included in the manuscript?	In which section is the information available? (Reagents and Tools Table, Materials and Methods, Figures, Data Availability Section)
**Laboratory animals or Model organisms:** Provide species, strain, sex, age, genetic modification status. Provide accession number in repository **OR** supplier name, catalog number, clone number, **OR** RRID.	Yes	Materials and methods page 12
**Animal observed in or captured from the field:** Provide species, sex, and age where possible**.**	Not Applicable	
Please detail **housing and husbandry conditions**.	Not Applicable	

Plants and microbes	Information included in the manuscript?	In which section is the information available? (Reagents and Tools Table, Materials and Methods, Figures, Data Availability Section)
**Plants:** provide species and strain, ecotype and cultivar where relevant, unique accession number if available, and source (including location for collected wild specimens).	Not Applicable	
**Microbes:** provide species and strain, unique accession number if available, and source.	Not Applicable	

Human research participants	Information included in the manuscript?	In which section is the information available? (Reagents and Tools Table, Materials and Methods, Figures, Data Availability Section)
If collected and within the bounds of privacy constraints report on age, sex and gender or ethnicity for all study participants.	Not Applicable	

Core facilities	Information included in the manuscript?	In which section is the information available? (Reagents and Tools Table, Materials and Methods, Figures, Data Availability Section)
If your work benefited from core facilities, was their service mentioned in the acknowledgments section?	Yes	Acknowledgements page 16

Design

Study protocol	Information included in the manuscript?	In which section is the information available? (Reagents and Tools Table, Materials and Methods, Figures, Data Availability Section)
If study protocol has been **pre-registered, provide DOI in the manuscript**. For clinical trials, provide the trial registration number **OR** cite DOI.	Not Applicable	
Report the **clinical trial registration number** (at ClinicalTrials.gov or equivalent), where applicable.	Not Applicable	

Laboratory protocol	Information included in the manuscript?	In which section is the information available? (Reagents and Tools Table, Materials and Methods, Figures, Data Availability Section)
Provide DOI OR other citation details if **external detailed step-by-step protocols** are available.	Not Applicable	

Experimental study design and statistics	Information included in the manuscript?	In which section is the information available? (Reagents and Tools Table, Materials and Methods, Figures, Data Availability Section)
Include a statement about **sample size** estimate even if no statistical methods were used.	Yes	Materials and methods page 13 describe sample size and statistics
Were any steps taken to minimize the effects of subjective bias when allocating animals/samples to treatment (e.g. **randomization procedure**)? If yes, have they been described?	Not Applicable	
Include a statement about **blinding** even if no blinding was done.	Not Applicable	
Describe **inclusion/exclusion criteria** if samples or animals were excluded from the analysis. Were the criteria pre-established? If sample or data points were omitted from analysis, report if this was due to attrition or intentional exclusion and provide justification.	Not Applicable	
For every figure, are **statistical tests** justified as appropriate? Do the data meet the assumptions of the tests (e.g., normal distribution)? Describe any methods used to assess it. Is there an estimate of variation within each group of data? Is the variance similar between the groups that are being statistically compared?	Yes	Materials and methods page 13 describe sample size and statistics

Sample definition and in-laboratory replication	Information included in the manuscript?	In which section is the information available? (Reagents and Tools Table, Materials and Methods, Figures, Data Availability Section)
In the figure legends: state number of times the experiment was **replicated** in laboratory.	Yes	Materials and methods page 13 and figure 5 caption
In the figure legends: define whether data describe **technical or biological replicates**.	Yes	Materials and methods page 13 and figure 5 caption

Ethics

Ethics	Information included in the manuscript?	In which section is the information available? (Reagents and Tools Table, Materials and Methods, Figures, Data Availability Section)
Studies involving **human participants**: State details of **authority granting ethics approval** (IRB or equivalent committee(s), provide reference number for approval.	Not Applicable	
Studies involving **human participants**: Include a statement confirming that **informed consent** was obtained from all subjects and that the experiments conformed to the principles set out in the WMA Declaration of Helsinki and the Department of Health and Human Services Belmont Report.	Not Applicable	
Studies involving **human participants:** For publication of **patient photos**, include a statement confirming that consent to publish was obtained.	Not Applicable	
Studies involving experimental **animals**: State details of **authority granting ethics approva**l (IRB or equivalent committee(s), provide reference number for approval. Include a statement of compliance with ethical regulations.	Not Applicable	
Studies involving **specimen and field samples:** State if relevant **permits** obtained, provide details of authority approving study; if none were required, explain why.	Not Applicable	

Dual Use Research of Concern (DURC)	Information included in the manuscript?	In which section is the information available? (Reagents and Tools Table, Materials and Methods, Figures, Data Availability Section)
Could your study fall under dual use research restrictions? Please check biosecurity documents and list of **select agents and toxins** (CDC): https://www.selectagents.gov/sat/list.htm	Not Applicable	
If you used a select agent, is the security level of the lab appropriate and reported in the manuscript?	Not Applicable	
If a study is subject to dual use research of concern regulations, is the name of the **authority granting approval and reference number** for the regulatory approval provided in the manuscript?	Not Applicable	

Reporting

The MDAR framework recommends adoption of discipline-specific guidelines, established and endorsed through community initiatives. Journals have their own policy about requiring specific guidelines and recommendations to complement MDAR.

Adherence to community standards	Information included in the manuscript?	In which section is the information available? (Reagents and Tools Table, Materials and Methods, Figures, Data Availability Section)
State if relevant guidelines or checklists (e.g., **ICMJE, MIBBI, ARRIVE, PRISMA**) have been followed or provided.	Not Applicable	
For **tumor marker prognostic studies,** we recommend that you follow the **REMARK** reporting guidelines (see link list at top right). See author guidelines, under 'Reporting Guidelines'. Please confirm you have followed these guidelines.	Not Applicable	
For **phase II and III randomized controlled trials**, please refer to the **CONSORT** flow diagram (see link list at top right) and submit the CONSORT checklist (see link list at top right) with your submission. See author guidelines, under 'Reporting Guidelines'. Please confirm you have submitted this list.	Not Applicable	

Data Availability

Data availability	Information included in the manuscript?	In which section is the information available? (Reagents and Tools Table, Materials and Methods, Figures, Data Availability Section)
Have **primary datasets** been deposited according to the journal's guidelines (see 'Data Deposition' section) and the respective accession numbers provided in the Data Availability Section?	Yes	Data availability section page 16
Were **human clinical and genomic datasets** deposited in a public access-controlled repository in accordance to ethical obligations to the patients and to the applicable consent agreement?	Not Applicable	
Are **computational models** that are central and integral to a study available without restrictions in a machine-readable form? Were the relevant accession numbers or links provided?	Yes	Data availability section page 16
If publicly available data were reused, provide the respective **data citations in the reference list**.	Yes	The data is cited with corresponding articles
