# Supplementary material for: Cell painting and thermal proteome profiling for inference of drug targets and mechanism of action
Source: Mol Syst Biol. 2026 May 15;22(8):1270–91. doi: 10.1038/s44320-026-00214-9 (PMC13434728; doi:10.1038/s44320-026-00214-9)
Supplement: Supplementary file 2 [file 44320_2026_214_MOESM2_ESM.pdf]

Appendix for Cell Painting and Thermal Proteome Profiling for Inference of Drug Targets and Mechanism of Action

List of Figures

Appendix Figure S1 Comparison of clustering algorithms for I-BET151 . . . . . S7
Appendix Figure S2 Comparison of clustering algorithms for (+)-JQ1 . . . . . S8
Appendix Figure S3 Heatmap for Silhouette Coefficients S9
Appendix Figure S4 Prevalence (count) of mechanism of action (MoA) annotations for compounds in CP cluster for (+)-JQ1 and I-BET151 . . . . . S16
Appendix Figure S5 Gene Onthology (GO) and Reactome enrichment analysis of I-BET151 PPI network graph. S17
Appendix Figure S6 Thermal proteome profiling (TPP) and Cell Painting analysis of Vemurafenib treated cells. S23
Appendix Figure S8 Thermal proteome profiling (TPP) and Cell Painting analysis of Panobinostat treated cells. S31
Appendix Figure S9 Gene Onthology (GO) and Reactome enrichment analysis of Panobinostat PPI network graph. S34
Appendix Figure S10 Thermal proteome profiling (TPP) and Cell Painting analysis of Crizotinib treated cells. S39
Appendix Figure S11 Gene Onthology (GO) and Reactome enrichment analysis of Crizotinib PPI network graph. S40
Appendix Figure S12 mRNA expression of known target and off-target proteins in the cell lines used for TPP and CP experiments for (+)-JQ1, I-BET151, Vemurafenib, Panobinostat and Crizotinib. S41
Appendix Figure S13 Validation of workflow performance using a high-throughput PISA data set [1]. S43
Appendix Figure S14 Receiver operating characteristic (ROC) curves for detection of true targets using our workflow on compounds 1–12 out of 40 compounds in the PISA data from Van Vranken et al where targets were detected by at lest one method. S44
Appendix Figure S15 Receiver operating characteristic (ROC) curves for detection of true targets using our workflow on compounds 13–24 out of 40 compounds in the PISA data from Van Vranken et al where targets were detected by at lest one method. S45
Appendix Figure S16 Receiver operating characteristic (ROC) curves for detection of true targets using our workflow on compounds 25–36 out of 40 compounds in the PISA data from Van Vranken et al. where targets were detected by at lest one method. S46
Appendix Figure S17 Receiver operating characteristic (ROC) curves for detection of true targets using our workflow on compounds 37–48 out of 40 compounds in the PISA data from Van Vranken et al. where targets were detected by at lest one method. S47

List of Tables

Appendix Table S1 Proteins with dose-dependent changes in thermal stability upon treatment with (+)-JQ1 in whole cells. S3
Appendix Table S2 Proteins with dose-dependent changes in thermal stability upon treatment with I-BET151 in whole cells. S4
Appendix Table S3 Betweenness centrality scores (BSC) and ranks for only TPP data, (+)-JQ1. . . . . S5
Appendix Table S4 Betweenness centrality scores (BSC) and ranks for only TPP data, I-BET151. . . . . S5
Appendix Table S5 Proteins with dose-dependent changes in thermal stability upon treatment with (+)-JQ1 in cell extract. S6
Appendix Table S6 Repeatability for SC3s clustring on CP data for (+)-JQ1. . . . . S10
Appendix Table S7 Repeatability for SC3s clustring on CP data for (+)-JQ1. . . . . S11
Appendix Table S8 Repeatability for SC3s clustring on CP data for I-BET151. . . . . S12
Appendix Table S9 Repeatability for SC3s clustring on CP data for Vemurafenib. S13
Appendix Table S10 Repeatability for SC3s clustring on CP data for Crizotinib. S14
Appendix Table S11 Repeatability for SC3s clustring on CP data for Panobinostat. S15
Appendix Table S12 Betweenness centrality scores (BSC) and ranks for only CP cluster data, (+)-JQ1 and I-BET151. . . . . S16
Appendix Table S13 Top 10 proteins with highest betweenness centrality score (BCS) for I-BET151. . . . . S18
Appendix Table S14 Significantly stabilized/destabilized proteins in ITDR TPP upon treatment with Vemurafenib. S19
Appendix Table S15 Betweenness centrality scores (BSC) and ranks for only TPP data, Vemurafenib. S20
Appendix Table S16 Betweenness centrality scores (BSC) and ranks for only CP cluster data, Vemurafenib. S21
Appendix Table S17 Top 10 proteins with highest betweenness centrality score (BCS) for Vemurafenib. S24
Appendix Table S18 Significantly stabilized/destabilized proteins in TR-TPP upon treatment with Panobinostat. S27
Appendix Table S19 Betweenness centrality scores (BSC) and ranks for only TPP data, Panobinostat. S28
Appendix Table S20 Betweenness centrality scores (BSC) and ranks for only CP cluster data, Panobinostat. S29
Appendix Table S21 Top 10 proteins with highest betweenness centrality score (BCS) for Panobinostat. S32
Appendix Table S22 Significantly stabilized/destabilized proteins in ITDR TPP upon treatment with Crizotinib. S35
Appendix Table S23 Betweenness centrality scores (BSC) and ranks for only TPP data, Crizotinib. S36
Appendix Table S24 Betweenness centrality scores (BSC) and ranks for only CP cluster data, Crizotinib. S37
Appendix Table S25 Top 10 proteins with highest betweenness centrality score (BCS) for Crizotinib. S41

Appendix Table S1: **Proteins with dose-dependent changes in thermal stability upon treatment with (+)-JQ1 in whole cells.** Proteins had to display a dose-dependent change for at least two consecutive temperature points to be considered. 67 proteins were detected in total, with 43 proteins being stabilized and 24 proteins destabilized.

Gene name	Uniprot ID	Stabilized/ Destabilized	pEC_{50}	No. neighb. temp. curves
GPX4	R4GNE4	Stabilized	5.2 - 5.2	2
NT5DC1	Q9H2R1	Stabilized	5.2 - 5.23	2
PITPNB	B3KYB7	Stabilized	5.27 - 5.28	2
ALDH3A2	K7EN73	Stabilized	5.2 - 6.45	5
TNFSF9	A0A0U5J8I0	Stabilized	6.34 - 7.17	3
YPEL5	P62699	Stabilized	5.2 - 6.61	2
ACADVL	K7EQP4	Stabilized	5.2 - 5.2	2
GLUD2	Q9BSD0	Stabilized	5.26 - 5.33	2
UGT8	D6RFW2	Stabilized	5.2 - 5.65	3
FNTA	H0YCW1	Stabilized	5.2 - 5.74	2
HARS2	D6RJE6	Stabilized	5.2 - 5.55	4
CDC42EP1	B0QYC8	Stabilized	5.2 - 5.69	2
FDFT1	Q6IAX1	Stabilized	5.2 - 5.4	3
C1QBP	I3L3Q7	Stabilized	5.2 - 5.21	4
PCYOX1L	E7EVZ5	Stabilized	5.2 - 5.22	2
FOS	Q6FG41	Stabilized	5.2 - 5.33	2
LOC650561	NA	Stabilized	5.2 - 6.27	2
TMEM143	M0R325	Stabilized	6.49 - 7.39	2
BRD2	X5CF57	Stabilized	5.22 - 7.5	6
MLKL	I3L4Z5	Stabilized	5.2 - 6.41	2
15 KDA PROTEIN.	NA	Stabilized	5.2 - 5.78	2
CRADD	Q8IY43	Stabilized	5.2 - 5.99	2
SLMO2	Q9Y3B1	Stabilized	5.61 - 6.44	2
HSD17B12	E9PI21	Stabilized	5.23 - 5.29	2
NSDHL	C9JDR0	Stabilized	5.22 - 5.34	3
SLC25A5	Q6NVC0	Stabilized	5.24 - 5.35	2
GART	Q71VH3	Stabilized	5.2 - 5.97	2
BANF1	E9PJJ8	Stabilized	5.2 - 5.21	2
EGR1	Q546S1	Stabilized	5.83 - 7.43	12
ADH5	Q9H1A0	Stabilized	5.2 - 5.24	2
KSR1	J3QSG8	Stabilized	5.2 - 5.72	2
MARS2	Q96GW9	Stabilized	5.65 - 5.81	2
CDKN1A	P38936	Stabilized	5.46 - 6.62	6
GSTM1	X5DR03	Stabilized	5.29 - 5.95	3
BRD3	L8E9I5	Stabilized	5.2 - 6.7	6
NPEPPS	H0YDG0	Stabilized	5.22 - 5.27	3
SLC7A5	Q2MCL6	Stabilized	5.2 - 5.31	2
HADHA	H0YFD6	Stabilized	5.2 - 5.45	2
YBX2	L8EAU5	Stabilized	5.2 - 6.09	2
BRD4	W8JJB1	Stabilized	5.22 - 6.67	6
GGPS1	C9J7M1	Stabilized	5.23 - 5.33	2
DNAJC1	Q5T1X2	Destabilized	5.2 - 5.24	2
SFRS10	P62995	Destabilized	5.2 - 5.86	2
PRKACB	B2RB89	Destabilized	5.54 - 6.34	3
HNRNPL	Q6NTA2	Destabilized	5.93 - 7.5	2
ACLY	K7ESG8	Destabilized	5.2 - 5.2	2
NDUFA11	K7EQ77	Destabilized	6.18 - 6.51	2
CLPTM1L	G5E9Z2	Destabilized	5.2 - 5.2	2
ZNF367	Q7RTV3	Destabilized	7.02 - 7.17	2
TM9SF4	F2Z2L1	Destabilized	5.2 - 5.62	4
SRD5A3	H0Y9P9	Destabilized	5.2 - 5.2	2
IDH2	H0YLL5	Destabilized	5.2 - 5.2	2
SLC35A2	Q6ICV6	Destabilized	5.2 - 5.2	2
MBOAT7	M0R1Z5	Destabilized	5.2 - 5.22	3
DCUN1D5	J3QQL8	Destabilized	5.22 - 5.24	2
ZMAT5	Q9UDW3	Destabilized	5.53 - 7.17	2
ERGIC2	H0YI58	Destabilized	5.2 - 5.23	2
AP3S1	F5H459	Destabilized	5.2 - 5.32	2
DPM1	Q5QPK2	Destabilized	5.2 - 5.2	2
CROP	Q86Y74	Destabilized	5.2 - 6.39	3
BCOR	H7C2V9	Destabilized	5.54 - 6.12	2
C7ORF42	Q9NWD8	Destabilized	5.26 - 5.62	2
ATP5F1	P24539	Destabilized	5.2 - 5.2	2
C9ORF5	Q9H330	Destabilized	5.22 - 5.3	3
PTPLAD1	Q9P035	Destabilized	5.85 - 6.13	2

Appendix Table S2: **Proteins with dose-dependent changes in thermal stability upon treatment with I-BET151 in whole cells.** Proteins had to display a dose-dependent change for at least two consecutive temperature points to be considered. 40 proteins were detected in total, with 18 proteins being stabilized and 22 proteins destabilized.

Gene name	Uniprot ID	Stabilized/ Destabilized	pEC_{50}	No. neighb. temp. curves
MARS2	Q96GW9	Stabilized	5.33 - 6.22	2
EGR1	Q546S1	Stabilized	5.2 - 5.4	6
HARS	B3KWE1	Stabilized	5.42 - 5.59	2
CPOX	D6RER6	Stabilized	5.21 - 5.24	3
NUDT1	C9J361	Stabilized	5.2 - 5.31	4
BRD3	L8E9I5	Stabilized	5.21 - 6.42	5
NPEPPS	H0YDG0	Stabilized	5.31 - 5.64	2
GLUD1	Q9UQV0	Stabilized	5.2 - 5.44	2
BRD2	X5CF57	Stabilized	5.24 - 7.0	4
SORD	H0YKB3	Stabilized	5.21 - 5.23	2
NSDHL	C9JDR0	Stabilized	5.21 - 5.44	2
ACADVL	K7EQP4	Stabilized	5.2 - 5.54	2
BRD4	W8JJB1	Stabilized	5.21 - 6.7	4
GLUD2	Q9BSD0	Stabilized	5.22 - 5.3	2
ADH5	Q9H1A0	Stabilized	5.22 - 5.22	2
SCCPDH	A0A384NPM7	Stabilized	5.27 - 5.27	2
HARS2	D6RJE6	Stabilized	5.2 - 5.47	6
SDR39U1	Q86TZ5	Stabilized	5.51 - 5.52	2
C6ORF129	Q9P0B6	Destabilized	5.4 - 7.06	2
NOVA2	Q9HDB7	Destabilized	5.2 - 7.03	6
PSPC1	X6RDA4	Destabilized	6.76 - 7.3	2
TFAM	Q6LES8	Destabilized	6.49 - 7.17	2
A6NLS3_HUMAN	NA	Destabilized	5.9 - 7.5	2
C8ORF83	Q629K1	Destabilized	6.74 - 7.33	2
DNAJC8	S4R3J5	Destabilized	6.12 - 7.5	2
NDUFB10	Q96RX5	Destabilized	6.7 - 7.5	2
TOR2A	Q8N2E6	Destabilized	5.53 - 7.13	2
BXDC1	Q9H7B2	Destabilized	7.23 - 7.26	2
NUCB2	Q2L696	Destabilized	5.83 - 7.22	2
SUDS3	Q52LB7	Destabilized	5.5 - 6.24	2
UBTF	Q9BQR2	Destabilized	7.48 - 7.5	2
CCDC49	Q9NXE8	Destabilized	6.76 - 7.5	2
RAD50	Q32P42	Destabilized	5.2 - 7.5	2
PES1	H7C267	Destabilized	6.54 - 6.61	2
CHMP6	I3L4G8	Destabilized	5.71 - 7.5	2
TOMM5	H0YFG9	Destabilized	6.47 - 7.5	2
ILKAP	H7C2I8	Destabilized	5.41 - 7.05	2
TNFAIP8L2	Q6P589	Destabilized	5.5 - 7.5	2
TPM3	Q5VU62	Destabilized	5.87 - 7.17	2
C14ORF166	Q549M8	Destabilized	5.86 - 7.5	2
POLR2A	A0AAG2TJB2	Destabilized	6.98 - 7.5	2

Appendix Table S3: **Betweenness centrality scores (BSC) and ranks for only TPP data, (+)-JQ1.**

Gene name	Uniprot ID	BSC	Rank	Known target
HNRNPL	P14866	0.559715	1	
BRD4	O60885	0.484848	2	Yes
BANF1	O75531	0.251931	3	
FOS	P01100	0.169340	4	
HACD3	Q9P035	0.165775	5	
BRD2	P25440	0.125074	6	Yes
HADHA	P40939	0.124480	7	Yes
HSD17B12	Q53GQ0	0.114082	8	
LUC7L3	O95232	0.094474	9	
DCUN1D5	Q9BTE7	0.058824	10	
NSDHL	Q15738	0.058824	11	
CDKN1A	P38936	0.058824	12	
DNAJC1	Q96KC8	0.058824	13	
BRD3	Q15059	0.048425	14	Yes
TRA2B	P62995	0.048128	15	
PWWP2B	Q6NUJ5	0.044563	16	
ATP5PB	P24539	0.002674	17	
SLC25A5	P05141	0.000000	18	
EGR1	P18146	0.000000	18	
ALDH3A2	P51648	0.000000	18	
GART	P22102	0.000000	18	
PRKACB	P22694	0.000000	18	
TMEM143	Q96AN5	0.000000	18	
MLKL	Q8NB16	0.000000	18	
NT5DC1	Q5TFE4	0.000000	18	
FDFT1	P37268	0.000000	18	
SRD5A3	Q9H8P0	0.000000	18	
BCOR	Q6W2J9	0.000000	18	
ACADVL	P49748	0.000000	18	
CRADD	P78560	0.000000	18	
NPEPPS	P55786	0.000000	18	
ACLY	P53396	0.000000	18	
TM9SF4	Q92544	0.000000	18	
ZMAT5	Q9UDW3	0.000000	18	
PCYOX1L	Q8NBM8	0.000000	18	

Appendix Table S4: **Betweenness centrality scores (BSC) and ranks for only TPP data, I-BET151.**

Gene name	Uniprot ID	BSC	Rank	Known target
BRD4	O60885	0.772904	1	Yes
PSPC1	Q8WXF1	0.242690	2	
DNAJC8	O75937	0.109162	3	
GLUD1	P00367	0.105263	4	
POLR2A	P24928	0.043860	5	
BRD2	P25440	0.029240	6	Yes
BRD3	Q15059	0.017544	7	Yes
RTRAF	Q9Y224	0.008772	8	
UBTF	P17480	0.005848	9	
PES1	O00541	0.003899	10	
TFAM	Q00059	0.000000	11	
GLUD2	P49448	0.000000	11	
RAD50	Q92878	0.000000	11	
PWWP2B	Q6NUJ5	0.000000	11	
NOVA2	Q9UNW9	0.000000	11	
ILKAP	Q9H0C8	0.000000	11	
EGR1	P18146	0.000000	11	
RPF2	Q9H7B2	0.000000	11	
SORD	Q00796	0.000000	11	
SUDS3	Q9H7L9	0.000000	11	

Appendix Table S5: Proteins with dose-dependent changes in thermal stability upon treatment with (+)-JQ1 in cell extract. Proteins had to display a dose-dependent change for at least two consecutive temperature points to be considered. 12 proteins were detected in total, with 7 proteins being stabilized and 5 proteins destabilized.

Gene name	Uniprot ID	Stabilized/ Destabilized	pEC_{50}	No. neighb. temp. curves
BRD3	L8E9I5	Stabilized	5.2 - 6.11	4
HADHA	H0YFD6	Stabilized	5.2 - 5.22	2
BRD2	X5CF57	Stabilized	5.2 - 6.28	4
GRB10	Q75MT1	Stabilized	5.2 - 5.53	2
HADHB	F5GZQ3	Stabilized	5.2 - 5.2	2
NRM	H0Y6T6	Stabilized	5.63 - 5.83	3
BRD4	W8JJB1	Stabilized	5.2 - 5.96	6
HMGA1	Q6IPL9	Destabilized	5.28 - 6.84	2
HMGN4	O00479	Destabilized	5.2 - 6.97	2
RNF41	F8VVY2	Destabilized	5.75 - 6.68	2
TOMM6	Q96B49	Destabilized	7.17 - 7.17	2
ANKRD40	K7ERW4	Destabilized	5.2 - 5.53	2

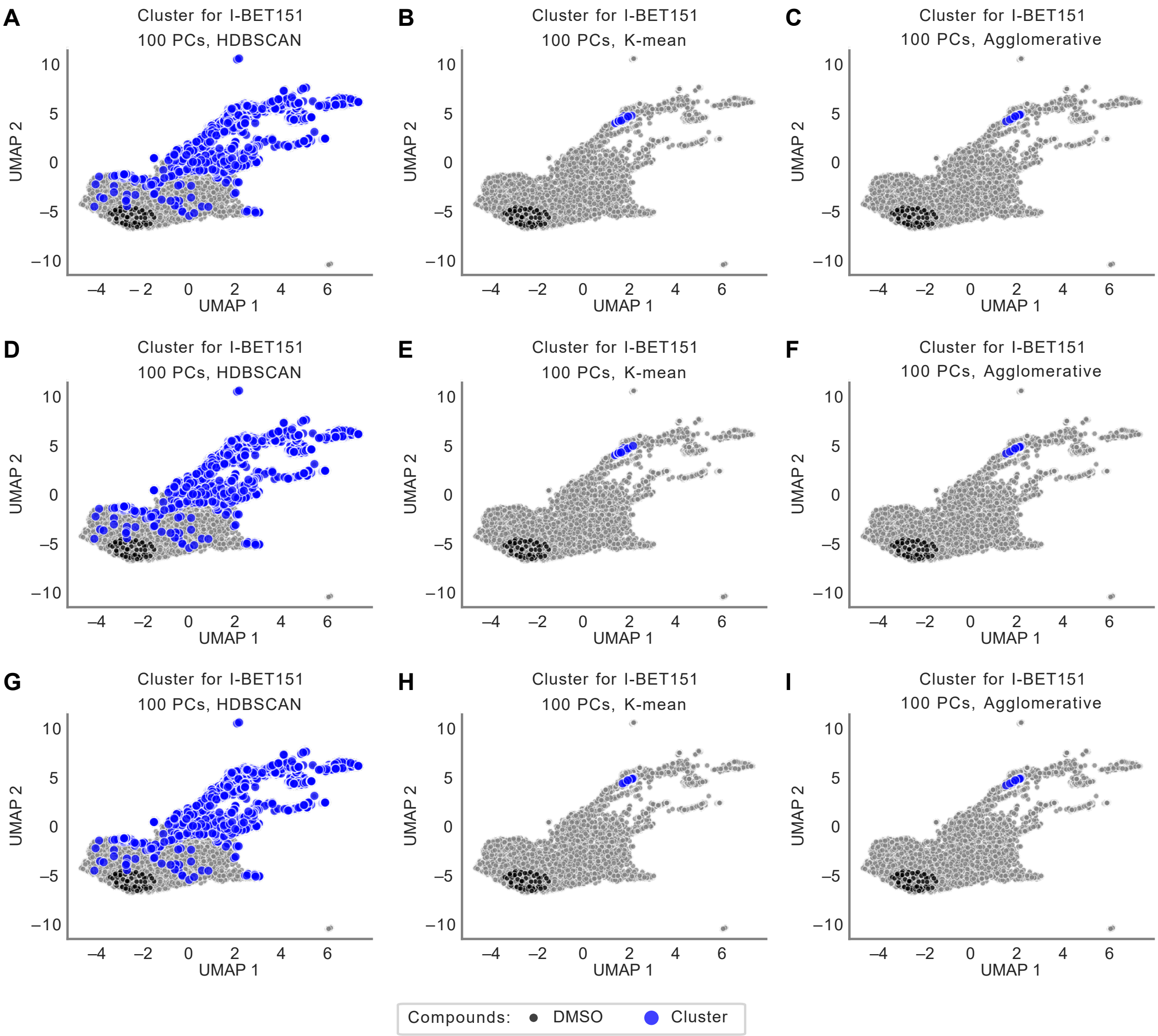

Appendix Figure S1: **Comparison of clustering algorithms for I-BET151** UMAP representations of clustering I-BET151 with **A, D, G**: HDBSCAN clustering; **B, E, H**: k-means clustering; **C, F, I**: agglomerative clustering. Each panel for HDBSCAN and k-means clustering was generated with random number seeds, while agglomerative clustering is deterministic which is showed with three identical panels. HDBSCAN was unable to cluster I-BET151 while both k-means and agglomerative clustering generated clusters of $\sim$20 compounds.

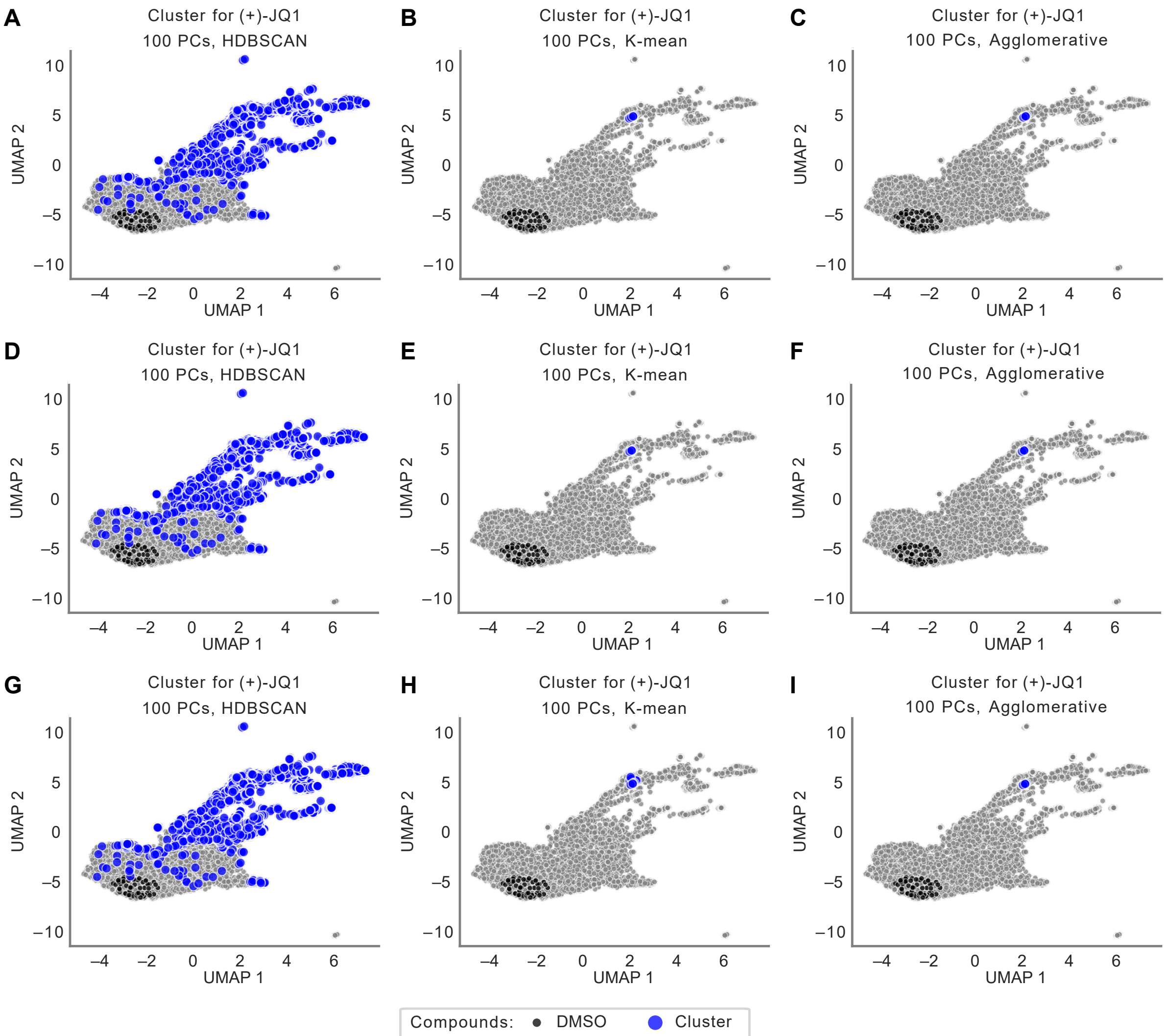

Appendix Figure S2: **Comparison of clustering algorithms for (+)-JQ1)** UMAP representations of clustering (+)-JQ1 with **A, D, G**: HDBSCAN clustering; **B, E, H**: *k*-means clustering; **C, F, I**: agglomerative clustering. Each panel for HDBSCAN and *k*-means clustering was generated with random number seeds, while agglomerative clustering is deterministic which is showed with three identical panels. HDBSCAN was unable to cluster (+)-JQ1 while both *k*-means and agglomerative clustering generated clusters of ∼20 compounds.

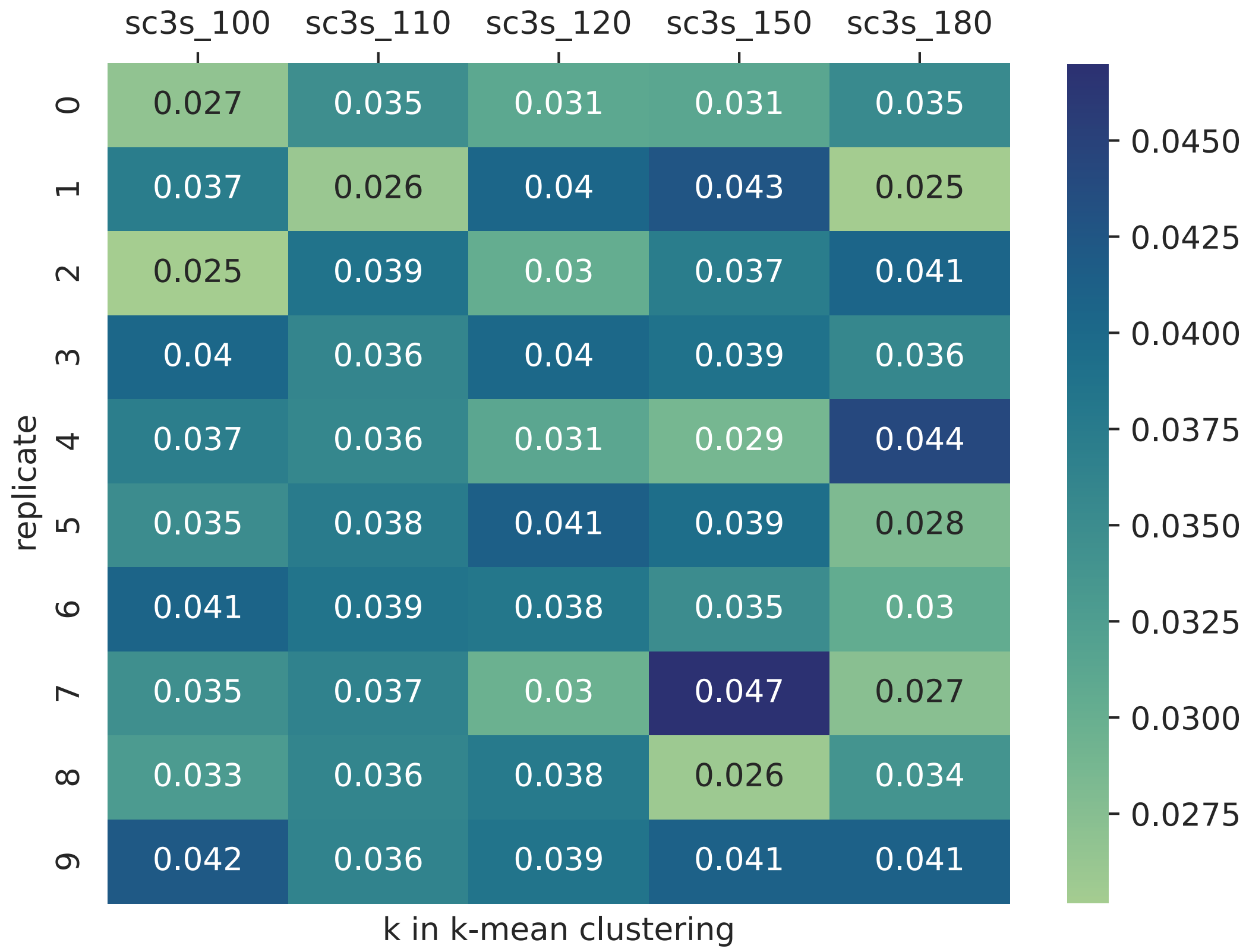

Appendix Figure S3: Heatmap for Silhouette Coefficients in 10 replicates of the SC3s clustering algorithm over five values of k (k: 100-180). The clustering was run on 14 principal components with n_runs = 2000.

Appendix Table S6: **Repeatability for SC3s clustring on CP data for (+)-JQ1.** The whole clustring was run on 14 principal components with n_runs = 1000.

	Percentage of samples present in cluster for at least 9 of 10 replicates, %					Number of samples in cluster				
Replicate	sc3s_100	sc3s_110	sc3s_120	sc3s_150	sc3s_180	sc3s_100	sc3s_110	sc3s_120	sc3s_150	sc3s_180
1	95.3	49.3	28.8	63.3	93.9	43	69	118	49	33
2	95.3	68.0	38.2	48.3	91.2	43	50	89	58	34
3	100.0	19.2	45.9	75.6	96.7	41	177	74	41	30
4	100.0	30.1	38.1	83.8	63.3	41	113	42	37	49
5	18.7	12.7	46.6	72.1	45.0	75	268	73	43	69
6	50.0	73.9	36.5	93.8	70.5	82	46	85	32	44
7	87.2	45.9	72.3	91.2	75.6	47	74	47	34	41
8	91.1	50.0	35.4	96.9	93.9	45	68	96	32	33
9	42.7	63.0	34.0	47.0	81.6	96	54	100	66	38
10	100.0	87.2	58.6	96.9	75.6	41	39	58	32	41

Appendix Table S7: **Repeatability for SC3s clustring on CP data for (+)-JQ1.** The whole clustring was run on 14 principal components with n_runs = 2000.

	Percentage of samples present in cluster for at least 9 of 10 replicates, %					Number of samples in cluster				
Replicate	sc3s_100	sc3s_110	sc3s_120	sc3s_150	sc3s_180	sc3s_100	sc3s_110	sc3s_120	sc3s_150	sc3s_180
0	6.8	46.1	97.6	83.7	48.8	133	76	41	49	43
1	34.9	100.0	93.0	95.3	4.5	86	41	43	43	314
2	64.7	80.4	74.1	95.3	40.5	51	51	54	43	37
3	71.7	87.2	88.9	91.1	33.3	46	47	45	45	63
4	73.3	28.7	15.6	95.3	52.5	45	143	256	43	40
5	68.8	18.8	81.6	59.4	70.0	48	208	49	69	30
6	27.3	95.3	69.0	89.1	62.5	121	43	58	46	32
7	42.9	100.0	53.3	95.3	44.7	77	41	75	43	47
8	27.3	95.3	93.0	100.0	51.2	121	43	43	40	41
9	76.7	42.7	88.9	46.0	33.3	43	96	45	87	63

Appendix Table S8: **Repeatability for SC3s clustring on CP data for I-BET151.** The whole clustring was run on 14 principal components with n_runs = 2000.

	Percentage of samples present in cluster for at least 9 of 10 replicates, %					Number of samples in cluster				
Replicate	sc3s_100	sc3s_110	sc3s_120	sc3s_150	sc3s_180	sc3s_100	sc3s_110	sc3s_120	sc3s_150	sc3s_180
0	8.6	46.1	97.6	83.7	32.6	243	76	41	49	43
1	34.9	100.0	93.0	95.3	9.9	86	41	43	43	71
2	58.8	80.4	74.1	95.3	37.8	51	51	54	43	37
3	65.2	87.2	88.9	91.1	22.2	46	47	45	45	63
4	66.7	28.7	15.6	95.3	35.0	45	143	256	43	40
5	62.5	18.8	81.6	59.4	46.7	48	208	49	69	30
6	24.8	95.3	69.0	89.1	43.8	121	43	58	46	32
7	39.0	100.0	53.3	95.3	29.8	77	41	75	43	47
8	24.8	95.3	93.0	100.0	34.1	121	43	43	40	41
9	69.8	42.7	88.9	46.0	22.2	43	96	45	87	63

Appendix Table S9: **Repeatability for SC3s clustring on CP data for Vemurafenib.** The whole clustring was run on 14 principal components with n_runs = 2000.

	Percentage of samples present in cluster for at least 9 of 10 replicates, %					Number of samples in cluster				
Replicate	sc3s_100	sc3s_110	sc3s_120	sc3s_150	sc3s_180	sc3s_100	sc3s_110	sc3s_120	sc3s_150	sc3s_180
0	2.9	33.1	27.1	3.9	4.6	138	124	133	51	65
1	4.4	63.1	1.8	4.9	3.1	90	65	113	41	96
2	6.0	28.1	35.6	2.9	2.1	67	139	104	68	47
3	2.1	30.1	71.2	6.9	4.3	141	136	52	29	69
4	2.1	50.0	62.7	2.1	5.6	192	82	59	97	54
5	4.3	31.3	60.7	4.2	7.3	93	128	61	48	41
6	5.5	75.9	32.5	7.1	6.8	73	54	114	28	44
7	5.2	29.3	43.5	2.9	3.2	77	140	85	69	93
8	3.0	93.2	25.3	5.7	42.9	101	44	146	35	7
9	4.4	58.6	33.3	2.4	3.3	91	70	111	83	90

Appendix Table S10: **Repeatability for SC3s clustring on CP data for Crizotinib.** The whole clustring was run on 14 principal components with n_runs = 2000.

	Percentage of samples present in cluster for at least 9 of 10 replicates, %					Number of samples in cluster				
Replicate	sc3s_100	sc3s_110	sc3s_120	sc3s_150	sc3s_180	sc3s_100	sc3s_110	sc3s_120	sc3s_150	sc3s_180
0	29.3	100.0	100.0	100.0	100.0	133	39	40	39	39
1	92.9	81.3	97.6	100.0	97.5	42	48	41	39	40
2	97.5	100.0	100.0	100.0	100.0	40	39	39	39	38
3	92.9	92.9	81.6	97.5	100.0	42	42	49	40	39
4	92.9	43.3	93.0	100.0	100.0	42	90	43	39	39
5	28.3	92.9	97.6	92.9	100.0	138	42	41	42	39
6	100.0	97.5	100.0	100.0	100.0	39	40	40	39	39
7	14.4	100.0	43.5	100.0	100.0	271	39	92	39	39
8	100.0	92.9	100.0	100.0	100.0	39	42	40	39	39
9	97.5	97.5	26.3	100.0	100.0	40	40	152	39	39

Appendix Table S11: **Repeatability for SC3s clustring on CP data for Panobinostat.** The whole clustring was run on 14 principal components with n_runs = 2000.

	Percentage of samples present in cluster for at least 9 of 10 replicates, %					Number of samples in cluster				
Replicate	sc3s_100	sc3s_110	sc3s_120	sc3s_150	sc3s_180	sc3s_100	sc3s_110	sc3s_120	sc3s_150	sc3s_180
0	46.5	94.0	95.9	72.0	70.0	101	50	49	50	50
1	94.0	94.0	94.0	90.0	70.0	50	50	50	40	50
2	38.8	94.0	92.2	97.3	100.0	121	50	51	37	35
3	97.9	97.9	100.0	100.0	96.8	48	48	47	32	31
4	95.9	88.7	92.2	75.0	68.6	49	53	51	48	51
5	92.2	95.9	94.0	90.0	97.2	51	49	50	40	36
6	94.0	97.9	97.6	87.8	97.2	50	48	41	41	36
7	100.0	94.0	45.2	90.0	70.0	47	50	104	40	50
8	97.5	97.2	94.0	70.6	70.0	40	36	50	51	50
9	94.0	94.0	94.0	70.6	58.3	50	50	50	51	60

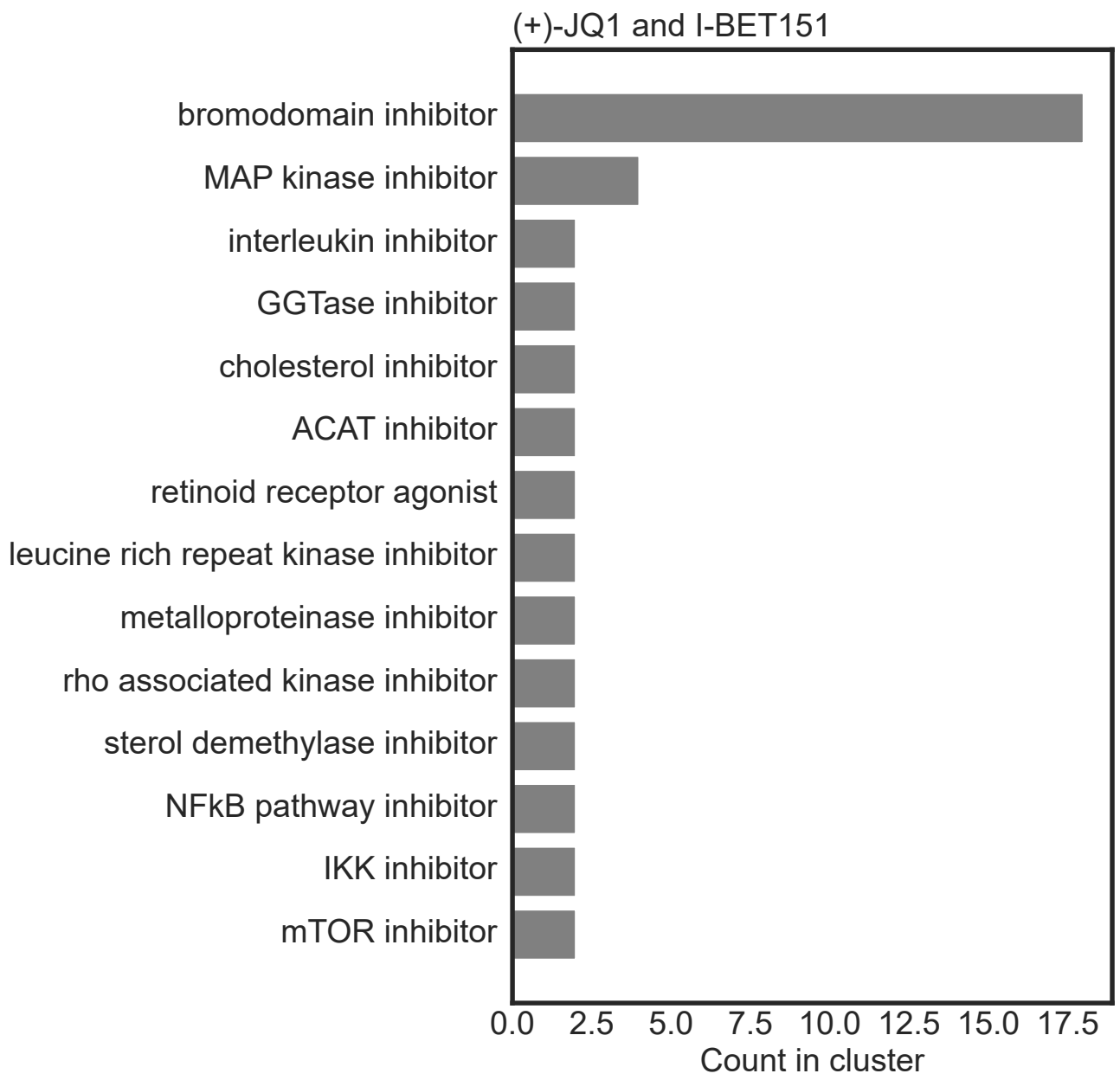

Appendix Figure S4: **Prevalence (count) of mechanism of action (MoA) annotations for compounds in CP cluster for (+)-JQ1 and I-BET151**

Appendix Table S12: **Betweenness centrality scores (BSC) and ranks for only CP cluster data, (+)-JQ1 and I-BET151.**

Gene name	Uniprot ID	BSC	Rank	Known target
ROCK1	Q13464	0.336508	1	
MMP9	P14780	0.287302	2	
BRD3	Q15059	0.266667	3	Yes
CDKN1A	P38936	0.190476	4	
MMP14	P50281	0.188889	5	
BRD4	O60885	0.147619	6	Yes
DMP1	Q13316	0.136508	7	
BRD2	P25440	0.117460	8	Yes
LRRK2	Q5S007	0.082540	9	
IKBKB	O14920	0.050794	10	
ROCK2	O75116	0.041270	11	
MMP2	P08253	0.026984	12	
MMP3	P08254	0.026984	13	
MTOR	P42345	0.014286	14	
MMP13	P45452	0.000000	15	
MAPK7	Q13164	0.000000	15	

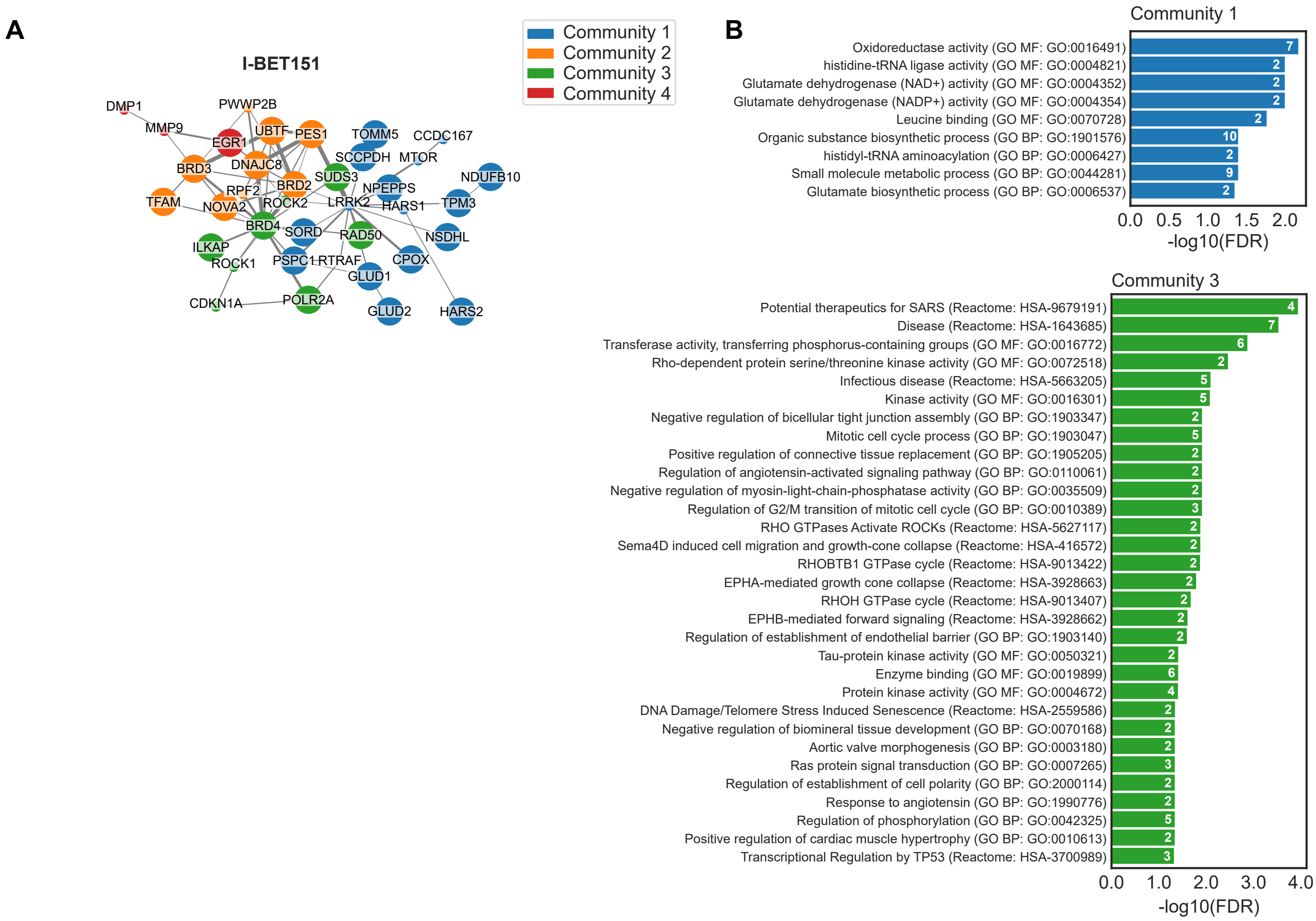

Appendix Figure S5: **Gene Onthology (GO) and Reactome enrichment analysis of I-BET151 PPI network graph. A.** Physical PPI network (same as in figure 3C) labeled by community. Communitites were identified using Clauset-Newman-Moore greedy modularity maximization. **B.** GO Molecular function (GO MF), GO Biological Process (GO BP), and Reactome pathway enrichment analysis for each community in A. Only enrichment with FDR ≤ 0.05 are shown.

Appendix Table S13: Top 10 proteins with highest betweenness centrality score (BCS) for I-BET151.

Uniprot ID	Gene name	Protein name	BCS	BCS rank change from TPP	Count in CP cluster
Q5S007 [2]	LRRK2	Leucine-rich repeat serine/threonine-protein kinase 2	0.59		2
O60885 [1]	BRD4	Bromodomain-containing protein 4	0.29	−1	14
P25440 [1]	BRD2	Bromodomain-containing protein 2	0.15	+4	4
Q15059 [1]	BRD3	Bromodomain-containing protein 3	0.11	+4	4
P14780 [2]	MMP9	Matrix metalloproteinase-9	0.06		2
O75937	DNAJC8	DnaJ homolog subfamily C member 8	0.06	−3	0
O75116 [2]	ROCK2	Rho-associated protein kinase 2	0.06		2
O00541	PES1	Pescadillo homolog	0.06	+2	0
Q8NBX0	SCCPDH	Saccharopine dehydrogenase-like oxidoreductase	0.06		0
P42345 [2]	MTOR	Serine/threonine-protein kinase mTOR	0.06		2

[1]Known target for I-BET151.

[2]Added from CP cluster

Appendix Table S14: **Significantly stabilized/destabilized proteins in ITDR TPP upon treatment with Vemurafenib.**

Gene names	Uniprot ID	Stabilized/Destabilized rep 1	Stabilized/Destabilized rep 2
ACOX1	K7ESC7	stabilized	NA
RAB2A	H7C125	stabilized	NA
BRAF	Q75MQ8	stabilized	stabilized
CNDP2	L8EAZ5	stabilized	NA
ATP1A1	Q5TC05	NA	destabilized
RBMX	H3BUY5	NA	destabilized
TRAP1	Q9BV61	destabilized	NA
DHRS4	H0YNP7	stabilized	NA
FECH	Q8TD50	stabilized	stabilized
NCL	L8EAF1	NA	destabilized
STX7	O15400	NA	stabilized
SPR	Q9UEC5	stabilized	NA
CTSB	Q8TAC7	destabilized	NA
NUCB1	H7BZI1	stabilized	stabilized
DSTN	F6RFD5	stabilized	NA
PIP4K2A	S4R320	NA	stabilized
PRKCSH	K7EPW7	stabilized	NA
IFITM1	A0A7P0Z452	NA	destabilized
PTPRC	Q9H3X6	NA	destabilized
P4HB	Q96C96	stabilized	NA
NUCB2	Q2L696	stabilized	stabilized
FLOT1	Q6IB58	NA	destabilized
ZAK	Q9NYL2	NA	stabilized
GART	Q71VH3	destabilized	NA
SDF4	H0Y3T6	NA	stabilized
RPS8	Q5JR95	NA	destabilized
PDCD10	H7C5M9	destabilized	NA
PCTP	Q549N3	stabilized	NA
MUTED	Q8TDH9	stabilized	NA
FTH1	Q6NZ44	NA	destabilized
LOC728658	NA	NA	destabilized
SARS	Q0VGA5	destabilized	NA
PPOX	Q96TC9	NA	stabilized
HNRPA1L-2	NA	NA	destabilized
ACAA1	H7C131	stabilized	NA
HSD17B4	E7ET17	stabilized	NA
PTGES2	X6RJ95	destabilized	NA
HPGD	E9PD69	NA	stabilized
DCK	D6RG38	NA	stabilized
TMEM159	Q96B96	NA	destabilized
ADFP	Q6FHZ7	destabilized	NA
BLVRB	M0R192	NA	destabilized
SEC22B	I1VE20	stabilized	NA
RPL5	R4GNJ2	NA	destabilized
ALDH4A1	Q5TF55	stabilized	stabilized
HADH	J3KR89	stabilized	stabilized
MTHFS	Q96EE9	destabilized	NA
MAP2K1	H3BRW9	destabilized	NA
AK3L1	A0A8I5KW96	destabilized	NA
HNRNPU	Q5RI18	NA	destabilized
RAD9A	F5H8A2	stabilized	NA
ALDH18A1	A0A2Z4QJH0	stabilized	NA
SLC1A5	Q71UA6	NA	destabilized
PIP4K2C	H0YIJ6	NA	stabilized
PRDX4	V9HW63	stabilized	NA
PTGFRN	Q4QQP8	NA	destabilized

Appendix Table S15: Betweenness centrality scores (BSC) and ranks for only TPP data, Vemurafenib.

Gene name	Uniprot ID	BSC	Rank	Known target
HNRNPU	Q00839	0.490493	1	
BRAF	P15056	0.461676	2	Yes
ACAA1	P09110	0.447415	3	
HSD17B4	P51659	0.383838	4	
NCL	P19338	0.359477	5	
RAB2A	P61019	0.169638	6	
P4HB	P07237	0.169340	7	
HADH	Q16836	0.165775	8	
TRAP1	Q12931	0.114082	9	
SPR	P35270	0.114082	10	
HPGD	P15428	0.114082	11	
STX7	O15400	0.102793	12	
FLOT1	O75955	0.079917	13	
SCP2	P22307	0.078728	14	
MAP2K1	Q02750	0.062983	15	
PIP4K2C	Q8TBX8	0.058824	16	
DSTN	P60981	0.058824	17	
PRKCSH	P14314	0.058824	18	
AK3	Q9UIJ7	0.058824	19	
FECH	P22830	0.058824	20	Yes
BLVRB	P30043	0.058824	21	
RPS8	P62241	0.039810	22	
FTH1	P02794	0.004159	23	
ALDH4A1	P30038	0.000000	24	
ACOX1	Q15067	0.000000	24	
SEC22B	O75396	0.000000	24	
RPL5	P46777	0.000000	24	
RBMX	P38159	0.000000	24	
PPOX	P50336	0.000000	24	
PTPRC	P08575	0.000000	24	
DCK	P27707	0.000000	24	
ATP1A1	P05023	0.000000	24	
CTSB	P07858	0.000000	24	
PIP4K2A	P48426	0.000000	24	
PRDX4	Q13162	0.000000	24	

Appendix Table S16: **Betweenness centrality scores (BSC) and ranks for only CP cluster data, Vemurafenib.**

Gene name	Uniprot ID	BSC	Rank	Known target
ESR1	P03372	0.369588	1	
EGFR	P00533	0.336388	2	
DRD2	P14416	0.239080	3	
MAPK14	Q16539	0.234892	4	
EP300	Q09472	0.108256	5	
BRAF	P15056	0.097763	6	Yes
KDR	P35968	0.078955	7	
CSK	P41240	0.056827	8	
RAF1	P04049	0.041675	9	Yes
LCK	P06239	0.036646	10	
PDGFC	Q9NRA1	0.026928	11	
HTR1B	P28222	0.020690	12	
HTR2C	P28335	0.020690	13	
HTR2A	P28223	0.020690	14	
BTK	Q06187	0.018966	15	
PDGFRB	P09619	0.018141	16	
IRAK4	Q9NWZ3	0.013218	17	
LIMK1	P53667	0.012452	18	
FLT1	P17948	0.010171	19	
ESR2	Q92731	0.003585	20	
ATR	Q13535	0.000739	21	
BRD3	Q15059	0.000739	22	
CREBBP	Q92793	0.000739	23	
ACVR1	Q04771	0.000000	24	
HTR2B	P41595	0.000000	24	
FLT4	P35916	0.000000	24	
F2R	P25116	0.000000	24	
SMO	Q99835	0.000000	24	
RIPK2	O43353	0.000000	24	
NDC80	O14777	0.000000	24	
TRPV1	Q8NER1	0.000000	24	

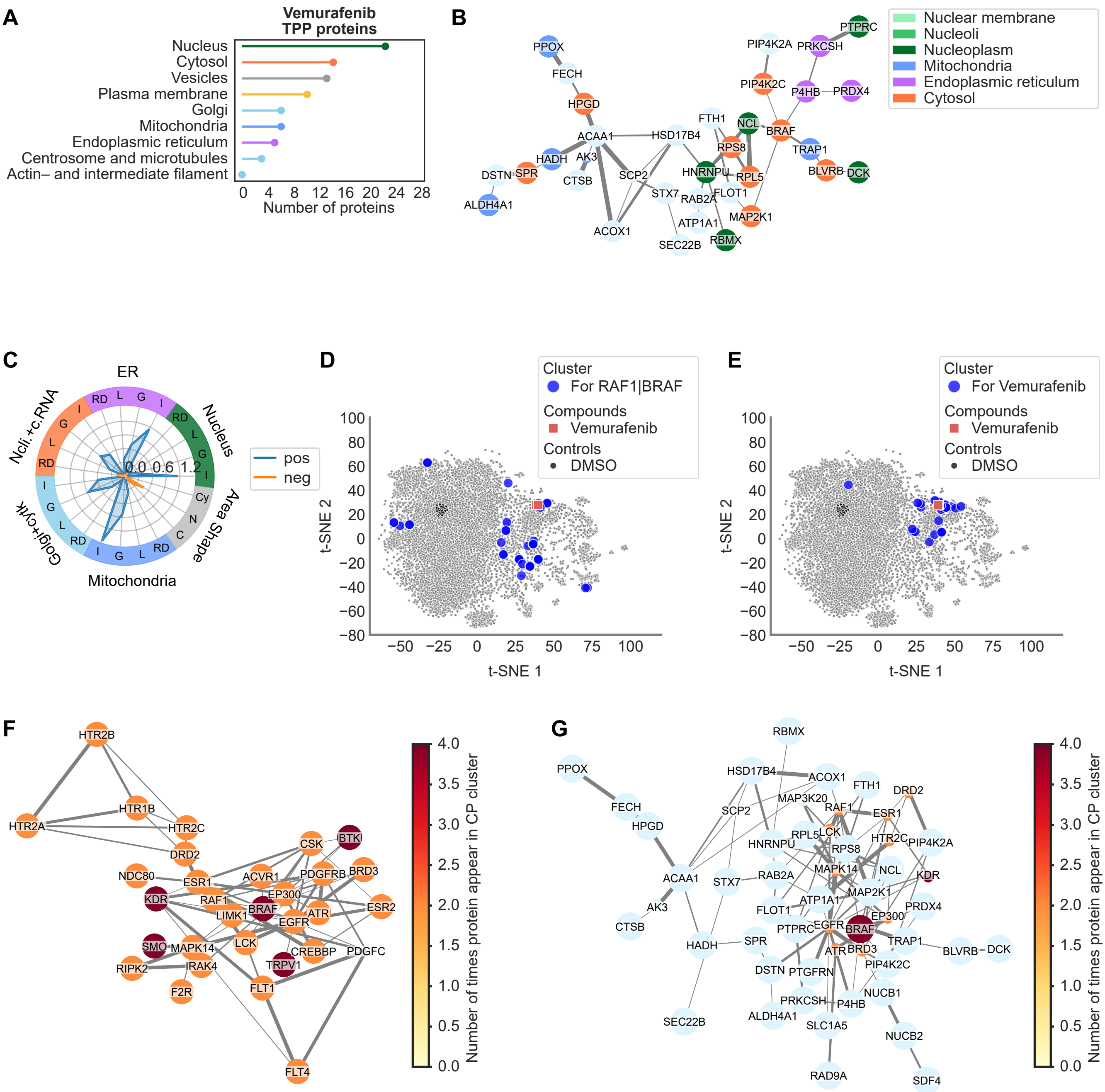
A
Vemurafenib TPP proteins
Nucleus
Cytosol
Vesicles
Plasma membrane
Golgi
Mitochondria
Endoplasmic reticulum
Centrosome and microtubules
Actin– and intermediate filament
Number of proteins
B
Nuclear membrane
Nucleoli
Nucleoplasm
Mitochondria
Endoplasmic reticulum
Cytosol
C
ER
Nucleus
Area Shape
Mitochondria
Golgi+cytk
Ncli.+c.RNA
pos
neg
D
Cluster
For RAF1|BRAF
Compounds
Vemurafenib
Controls
DMSO
t-SNE 1
t-SNE 2
E
Cluster
For Vemurafenib
Compounds
Vemurafenib
Controls
DMSO
t-SNE 1
t-SNE 2
F
Number of times protein appear in CP cluster
G
Number of times protein appear in CP cluster

Appendix Figure S6: **Thermal proteome profiling (TPP) and Cell Painting analysis of Vemurafenib treated cells. A.** Lollipop chart for subcellular location of the 56 proteins found to be either stabilized or destabilized in TPP for Vemurafenib. Subcellular location of each protein, based on immunohistochemistry and confocal microscopy, was retrieved from the Human Protein Atlas (proteinatlas.org). When a protein had more than one subcellular location assigned, that protein was included once per subcellular location in the graph. **B.** Physical protein-protein interaction (PPI) networks for the proteins found to be either stabilized or destabilized in TPP. PPI networks were retrieved from the STRING db. Nodes are colored by subcellular location (from Human Protein Atlas). Large nodes correspond to proteins detected by TPP, where small nodes are additionally added nodes from the STRING db during network retrieval. **C.** Radar chart for features in cell painting data for Vemurafenib. Features were grouped into categories based on two criteria: (i) Cell Profiler module, i.e. Intensity (I), Correlation (C), Granularity (G), Location (L) and RadialDistribution (RD); and (ii) stains, i.e. Nucleus (Hoechst), ER (Concanavalin A), Nucleoli and cytoplasmic RNA (SYTO14), Golgi apparatus and F-actin cytoskeleton (WGA and Phalloidin) and Mitochondria (Mitotracker). Features were only consider for the object Cell, except for features from the stain for Nucleus, which was only considered in the object Nucleus. Additionally, area-shape related features were grouped by cell compartment, i.e. Cell (C), Cytoplasm (Cy) and Nucleus (N). **D.** t-SNE for the morphological features in the SPECS cell painting data on U2OS cells. Blue dots show the location of all cells treated with compounds sharing at least one target with Vemurafenib (RAF1 or BRAF). **E.** t-SNE for CP SPECS data showing Vemurafenib (red) and a cluster of similar compounds (blue) identified using the consensus clustering algorithm SC3s on 13 principal components. K for the K-mean clustering algorithm was varied between 100 and 180. **F.** Physical PPI network for known protein targets of the compounds identified in the cluster in F. Vemurafenib was blinded from the compound list before retrieving known targets. Network is colored by the number of times a protein target is present within the cluster. Large nodes indicate proteins also detected in TPP. **G.** Physical PPI network combining proteins identified in TPP and CP cluster, after applying a betweeness centrality calculation on the nodes from CP clustering. The number of CP supplied nodes to keep was chosen as the minimum number of nodes needed to maximize connection between the TPP identified proteins. The nodes are colored by number of times a protein target is present within the cluster in E.

Appendix Table S17: Top 10 proteins with highest betweenness centrality score (BCS) for Vemurafenib.

Uniprot ID	Gene name	Protein name	BCS	Count in CP cluster
P00533 [2]	EGFR	Epidermal growth factor receptor	0.35	2
P04049 [1 2]	RAF1	RAF proto-oncogene serine/threonine-protein kinase	0.20	2
P09110	ACAA1	3-ketoacyl-CoA thiolase, peroxisomal	0.20	0
P15056 [1]	BRAF	Serine/threonine-protein kinase B-raf	0.17	4
Q15059 [1]	BRD3	Bromodomain-containing protein 3	0.15	4
Q15067	ACOX1	Peroxisomal acyl-coenzyme A oxidase 1	0.14	0
Q00839	HNRNPU	Heterogeneous nuclear ribonucleoprotein U	0.13	0
Q09472 [2]	EP300	Histone acetyltransferase p300	0.09	2
P60981	DSTN	Destrin	0.08	0
P61019	RAB2A	Ras-related protein Rab-2A	0.08	0

[1]Known target for Vemurafenib.

[2]Added from CP cluster

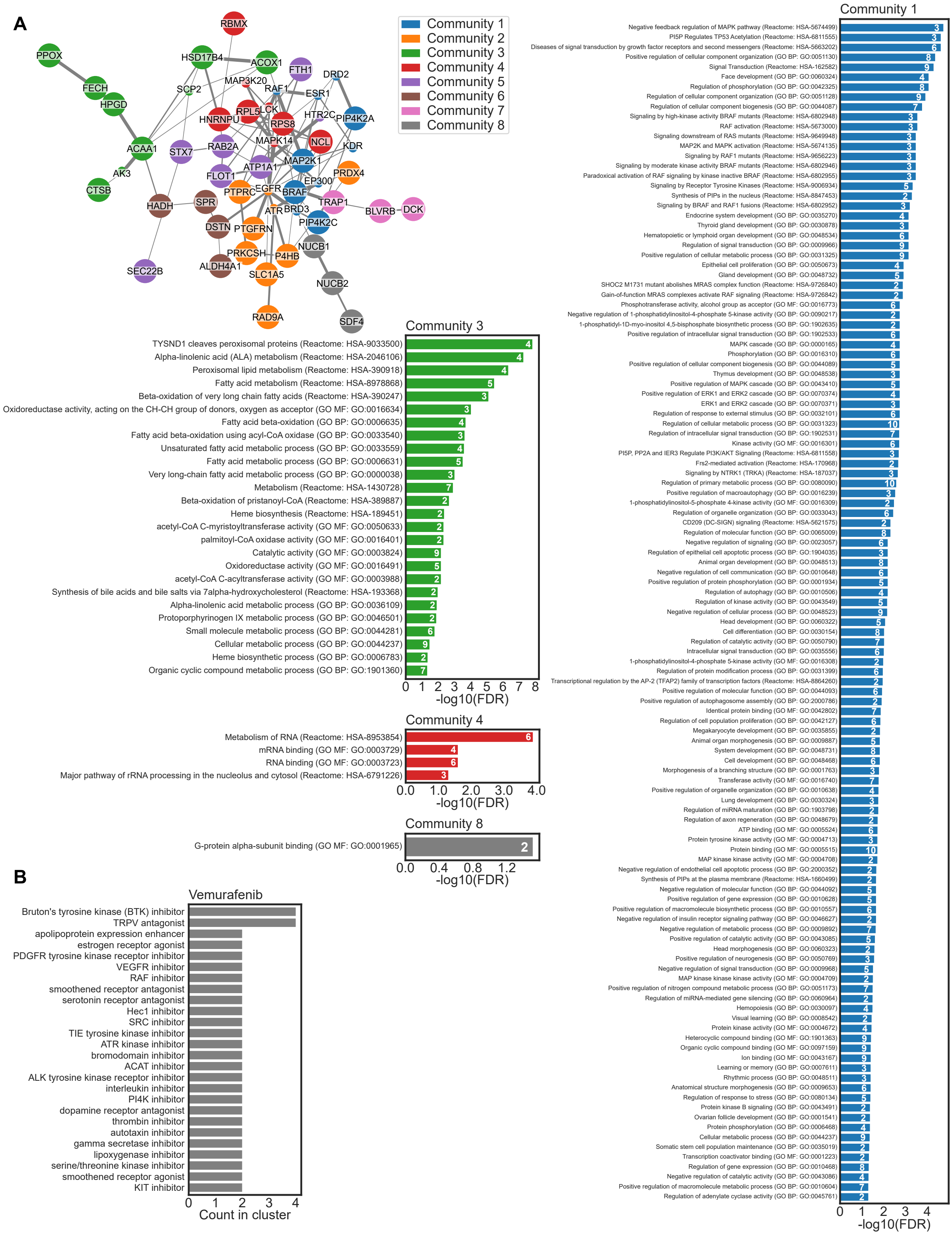
A
RBMX
PPOX
HSD17B4
ACOX1
FTH1
DRD2
MAP3K20
SCP2
FECH
RAF1
ESR1
HPGD
RPL5
LCK
HTR2C
PIP4K2A
HNRNPU
RPS8
MAPK14
NCL
KDR
ACAA1
STX7
RAB2A
AK3
ATP1A1
MAP2K1
FLOT1
PRDX4
CTSB
EP300
PTPRC
EGFR
BRAF
HADH
SPR
TRAP1
ATR
BRD3
BLVRB
DCK
DSTN
PTGFRN
PIP4K2C
PRKCSH
P4HB
NUCB1
SEC22B
ALDH4A1
SLC1A5
NUCB2
RAD9A
SDF4
Community 1
Community 2
Community 3
Community 4
Community 5
Community 6
Community 7
Community 8
Community 1
Negative feedback regulation of MAPK pathway (Reactome: HSA-5674499) 3
PI5P Regulates TP53 Acetylation (Reactome: HSA-6811555) 3
Diseases of signal transduction by growth factor receptors and second messengers (Reactome: HSA-5663202) 6
Positive regulation of cellular component organization (GO BP: GO:0051130) 8
Signal Transduction (Reactome: HSA-162582) 9
Face development (GO BP: GO:0060324) 4
Regulation of phosphorylation (GO BP: GO:0042325) 8
Regulation of cellular component organization (GO BP: GO:0051128) 9
Regulation of cellular component biogenesis (GO BP: GO:0044087) 7
Signaling by high-kinase activity BRAF mutants (Reactome: HSA-6802948) 3
RAF activation (Reactome: HSA-5673000) 3
Signaling downstream of RAS mutants (Reactome: HSA-9649948) 3
MAP2K and MAPK activation (Reactome: HSA-5674135) 3
Signaling by RAF1 mutants (Reactome: HSA-9656223) 3
Signaling by moderate kinase activity BRAF mutants (Reactome: HSA-6802946) 3
Paradoxical activation of RAF signaling by kinase inactive BRAF (Reactome: HSA-6802955) 3
Signaling by Receptor Tyrosine Kinases (Reactome: HSA-9006934) 5
Synthesis of PIPs in the nucleus (Reactome: HSA-8847453) 2
Signaling by BRAF and RAF1 fusions (Reactome: HSA-6802952) 3
Endocrine system development (GO BP: GO:0035270) 4
Thyroid gland development (GO BP: GO:0030878) 3
Hematopoietic or lymphoid organ development (GO BP: GO:0048534) 6
Regulation of signal transduction (GO BP: GO:0009966) 9
Positive regulation of cellular metabolic process (GO BP: GO:0031325) 9
Epithelial cell proliferation (GO BP: GO:0050673) 4
Gland development (GO BP: GO:0048732) 5
SHOC2 M1731 mutant abolishes MRAS complex function (Reactome: HSA-9726840) 2
Gain-of-function MRAS complexes activate RAF signaling (Reactome: HSA-9726842) 2
Phosphotransferase activity, alcohol group as acceptor (GO MF: GO:0016773) 6
Negative regulation of 1-phosphatidylinositol-4-phosphate 5-kinase activity (GO BP: GO:0090217) 2
1-phosphatidyl-1D-myo-inositol 4,5-bisphosphate biosynthetic process (GO BP: GO:1902635) 2
Positive regulation of intracellular signal transduction (GO BP: GO:1902533) 6
MAPK cascade (GO BP: GO:0000165) 4
Phosphorylation (GO BP: GO:0016310) 6
Positive regulation of cellular component biogenesis (GO BP: GO:0044089) 5
Thymus development (GO BP: GO:0048538) 3
Positive regulation of MAPK cascade (GO BP: GO:0043410) 5
Positive regulation of ERK1 and ERK2 cascade (GO BP: GO:0070374) 4
ERK1 and ERK2 cascade (GO BP: GO:0070371) 3
Regulation of response to external stimulus (GO BP: GO:0032101) 6
Regulation of cellular metabolic process (GO BP: GO:0031323) 10
Regulation of intracellular signal transduction (GO BP: GO:1902531) 7
Kinase activity (GO MF: GO:0016301) 6
PI5P, PP2A and IER3 Regulate PI3K/AKT Signaling (Reactome: HSA-6811558) 3
Frs2-mediated activation (Reactome: HSA-170968) 2
Signaling by NTRK1 (TRKA) (Reactome: HSA-187037) 3
Regulation of primary metabolic process (GO BP: GO:0080090) 10
Positive regulation of macroautophagy (GO BP: GO:0016239) 3
1-phosphatidylinositol-5-phosphate 4-kinase activity (GO MF: GO:0016309) 2
Regulation of organelle organization (GO BP: GO:0033043) 6
CD209 (DC-SIGN) signaling (Reactome: HSA-5621575) 2
Regulation of molecular function (GO BP: GO:0065009) 8
Negative regulation of signaling (GO BP: GO:0023057) 6
Regulation of epithelial cell apoptotic process (GO BP: GO:1904035) 3
Animal organ development (GO BP: GO:0048513) 8
Negative regulation of cell communication (GO BP: GO:0010648) 6
Positive regulation of protein phosphorylation (GO BP: GO:0001934) 5
Regulation of autophagy (GO BP: GO:0010506) 4
Regulation of kinase activity (GO BP: GO:0043549) 5
Negative regulation of cellular process (GO BP: GO:0048523) 9
Head development (GO BP: GO:0060322) 5
Cell differentiation (GO BP: GO:0030154) 8
Regulation of catalytic activity (GO BP: GO:0050790) 7
Intracellular signal transduction (GO BP: GO:0035556) 6
1-phosphatidylinositol-4-phosphate 5-kinase activity (GO MF: GO:0016308) 2
Regulation of protein modification process (GO BP: GO:0031399) 6
Transcriptional regulation by the AP-2 (TFAP2) family of transcription factors (Reactome: HSA-8864260) 2
Positive regulation of molecular function (GO BP: GO:0044093) 6
Positive regulation of autophagosome assembly (GO BP: GO:2000786) 2
Identical protein binding (GO MF: GO:0042802) 7
Regulation of cell population proliferation (GO BP: GO:0042127) 6
Megakaryocyte development (GO BP: GO:0035855) 2
Animal organ morphogenesis (GO BP: GO:0009887) 5
System development (GO BP: GO:0048731) 8
Cell development (GO BP: GO:0048468) 6
Morphogenesis of a branching structure (GO BP: GO:0001763) 3
Transferase activity (GO MF: GO:0016740) 7
Positive regulation of organelle organization (GO BP: GO:0010638) 4
Lung development (GO BP: GO:0030324) 3
Regulation of miRNA maturation (GO BP: GO:1903798) 2
Regulation of axon regeneration (GO BP: GO:0048679) 2
ATP binding (GO MF: GO:0005524) 6
Protein tyrosine kinase activity (GO MF: GO:0004713) 3
Protein binding (GO MF: GO:0005515) 10
MAP kinase kinase activity (GO MF: GO:0004708) 2
Negative regulation of endothelial cell apoptotic process (GO BP: GO:2000352) 2
Synthesis of PIPs at the plasma membrane (Reactome: HSA-1660499) 2
Negative regulation of molecular function (GO BP: GO:0044092) 5
Positive regulation of gene expression (GO BP: GO:0010628) 5
Positive regulation of macromolecule biosynthetic process (GO BP: GO:0010557) 6
Negative regulation of insulin receptor signaling pathway (GO BP: GO:0046627) 2
Negative regulation of metabolic process (GO BP: GO:0009892) 7
Positive regulation of catalytic activity (GO BP: GO:0043085) 5
Head morphogenesis (GO BP: GO:0060323) 2
Positive regulation of neurogenesis (GO BP: GO:0050769) 3
Negative regulation of signal transduction (GO BP: GO:0009968) 5
MAP kinase kinase kinase activity (GO MF: GO:0004709) 2
Positive regulation of nitrogen compound metabolic process (GO BP: GO:0051173) 7
Regulation of miRNA-mediated gene silencing (GO BP: GO:0060964) 2
Hemopoiesis (GO BP: GO:0030097) 4
Visual learning (GO BP: GO:0008542) 2
Protein kinase activity (GO MF: GO:0004672) 4
Heterocyclic compound binding (GO MF: GO:1901363) 9
Organic cyclic compound binding (GO MF: GO:0097159) 9
Ion binding (GO MF: GO:0043167) 9
Learning or memory (GO BP: GO:0007611) 3
Rhythmic process (GO BP: GO:0048511) 3
Anatomical structure morphogenesis (GO BP: GO:0009653) 6
Regulation of response to stress (GO BP: GO:0080134) 5
Protein kinase B signaling (GO BP: GO:0043491) 2
Ovarian follicle development (GO BP: GO:0001541) 2
Protein phosphorylation (GO BP: GO:0006468) 4
Cellular metabolic process (GO BP: GO:0044237) 9
Somatic stem cell population maintenance (GO BP: GO:0035019) 2
Transcription coactivator binding (GO MF: GO:0001223) 2
Regulation of gene expression (GO BP: GO:0010468) 8
Negative regulation of catalytic activity (GO BP: GO:0043086) 4
Positive regulation of macromolecule metabolic process (GO BP: GO:0010604) 7
Regulation of adenylate cyclase activity (GO BP: GO:0045761) 2
0 1 2 3 4
-log10(FDR)
Community 3
TYSND1 cleaves peroxisomal proteins (Reactome: HSA-9033500) 4
Alpha-linolenic acid (ALA) metabolism (Reactome: HSA-2046106) 4
Peroxisomal lipid metabolism (Reactome: HSA-390918) 4
Fatty acid metabolism (Reactome: HSA-8978868) 5
Beta-oxidation of very long chain fatty acids (Reactome: HSA-390247) 3
Oxidoreductase activity, acting on the CH-CH group of donors, oxygen as acceptor (GO MF: GO:0016634) 3
Fatty acid beta-oxidation (GO BP: GO:0006635) 4
Fatty acid beta-oxidation using acyl-CoA oxidase (GO BP: GO:0033540) 3
Unsaturated fatty acid metabolic process (GO BP: GO:0033559) 4
Fatty acid metabolic process (GO BP: GO:0006631) 5
Very long-chain fatty acid metabolic process (GO BP: GO:0000038) 3
Metabolism (Reactome: HSA-1430728) 7
Beta-oxidation of pristanoyl-CoA (Reactome: HSA-389887) 2
Heme biosynthesis (Reactome: HSA-189451) 2
acetyl-CoA C-myristoyltransferase activity (GO MF: GO:0050633) 2
palmitoyl-CoA oxidase activity (GO MF: GO:0016401) 2
Catalytic activity (GO MF: GO:0003824) 9
Oxidoreductase activity (GO MF: GO:0016491) 5
acetyl-CoA C-acyltransferase activity (GO MF: GO:0003988) 2
Synthesis of bile acids and bile salts via 7alpha-hydroxycholesterol (Reactome: HSA-193368) 2
Alpha-linolenic acid metabolic process (GO BP: GO:0036109) 2
Protoporphyrinogen IX metabolic process (GO BP: GO:0046501) 2
Small molecule metabolic process (GO BP: GO:0044281) 6
Cellular metabolic process (GO BP: GO:0044237) 9
Heme biosynthetic process (GO BP: GO:0006783) 2
Organic cyclic compound metabolic process (GO BP: GO:1901360) 7
0 1 2 3 4 5 6 7 8
-log10(FDR)
Community 4
Metabolism of RNA (Reactome: HSA-8953854) 6
mRNA binding (GO MF: GO:0003729) 4
RNA binding (GO MF: GO:0003723) 6
Major pathway of rRNA processing in the nucleolus and cytosol (Reactome: HSA-6791226) 3
0.0 1.0 2.0 3.0 4.0
-log10(FDR)
Community 8
G-protein alpha-subunit binding (GO MF: GO:0001965) 2
0.0 0.4 0.8 1.2
-log10(FDR)
B
Vemurafenib
Bruton's tyrosine kinase (BTK) inhibitor
TRPV antagonist
apolipoprotein expression enhancer
estrogen receptor agonist
PDGFR tyrosine kinase receptor inhibitor
VEGFR inhibitor
RAF inhibitor
smoothened receptor antagonist
serotonin receptor antagonist
Hec1 inhibitor
SRC inhibitor
TIE tyrosine kinase inhibitor
ATR kinase inhibitor
bromodomain inhibitor
ACAT inhibitor
ALK tyrosine kinase receptor inhibitor
interleukin inhibitor
PI4K inhibitor
dopamine receptor antagonist
thrombin inhibitor
autotaxin inhibitor
gamma secretase inhibitor
lipoxygenase inhibitor
serine/threonine kinase inhibitor
smoothened receptor agonist
KIT inhibitor
0 1 2 3 4
Count in cluster

Appendix Figure S7: **Gene Onthology (GO) and Reactome enrichment analysis of Vemurafenib PPI network graph. A.** Physical PPI network (same as in figure S4G) labeled by community. Communitites were identified using Clauset-Newman-Moore greedy modularity maximization. GO Molecular function (GO MF), GO Biological Process (GO BP), and Reactome pathway enrichment analysis for each community in graph are also shown as bar charts. Only enrichment with FDR ≤ 0.05 are shown. **B.** Prevalence (count) of mechanism of action (MoA) annotations for compounds in CP cluster for Vemurafenib (shown in figure S4E)

Appendix Table S18: **Significantly stabilized/destabilized proteins in TR-TPP upon treatment with Panobinostat.**

Gene names	Uniprot ID	Stabilized/Destabilized
BAG2	O95816	Stabilized
CDK16	H0YC60	Stabilized
H2AFV—H2AFZ	NA	Stabilized
HDAC10	Q08AP5	Stabilized
HDAC6	Q9BRX7	Stabilized
HDAC8	F8WCG4	Stabilized
IQSEC2	L8E757	Stabilized
NFAT5	J3QKS5	Stabilized
STX4	H3BMK2	Stabilized
TTC38	H7C2L7	Stabilized
ZFYVE28	Q49AA1	Stabilized
ACTR10	V9GYX7	Detabilized
ARMC8	H7C5H2	Detabilized
CCT2	V9HW96	Detabilized
CCT6A	A1JUI8	Detabilized
CCT7	Q6IBT3	Detabilized
DDX23	H0YIL9	Detabilized
KARS	Q15046	Detabilized
LONP1	Q2VPA0	Detabilized
MASTL	A0A087WUU7	Detabilized
MKLN1	F8WEY7	Detabilized
PSMC1	Q53XL8	Detabilized
PSMC4	A8K2M0	Detabilized
PSMC5	J3QSE0	Detabilized
PSMD1	Q05CW6	Detabilized
PSMD11	J3QS13	Detabilized
PSMD13	J3KNQ3	Detabilized
PSMD8	R4GMR5	Detabilized
PTAR1	X6R9N0	Detabilized
PTCD3	F8WE76	Detabilized
RANBP9	Q96S59	Detabilized
RMND5A	Q9H871	Detabilized
SLC29A1	C8KHU2	Detabilized
VPRBP	Q9Y4B6	Detabilized
WDR26	L8EAE8	Detabilized

Appendix Table S19: **Betweenness centrality scores (BSC) and ranks for only TPP data, Panobinostat.**

Gene name	Uniprot ID	BSC	Rank	Known target
HDAC6	Q9UBN7	0.413514	1	Yes
PSMC5	P62195	0.165622	2	
RMND5A	Q9H871	0.135159	3	
PSMD1	Q99460	0.126755	4	
HDAC8	Q9BY41	0.126482	5	Yes
PSMC4	P43686	0.094203	6	
ARMC8	Q8IUR7	0.093582	7	
BAG2	O95816	0.092264	8	
MKLN1	Q9UL63	0.061632	9	
LONP1	P36776	0.046189	10	
RANBP9	Q96S59	0.042490	11	
H2AZ2	P0C0S5	0.039526	12	
CCT7	Q99832	0.024374	13	
CCT2	O60583	0.024374	14	
PSMD13	Q9UNM6	0.000000	15	
PSMC1	P62191	0.000000	15	
PSMD11	O00231	0.000000	15	
CCDC74B	Q96LY2	0.000000	15	
PSMD8	P48556	0.000000	15	
HDAC10	Q969S8	0.000000	15	Yes
CCT6A	P40227	0.000000	15	
WDR26	Q9H7D7	0.000000	15	
PTCD3	Q96EY7	0.000000	15	
DCAF1	Q9Y4B6	0.000000	15	

Appendix Table S20: **Betweenness centrality scores (BSC) and ranks for only CP cluster data, Panobinostat.**

Gene name	Uniprot ID	BSC	Rank	Known target
ESR1	P03372	0.127914	1	
AKT1	P31749	0.116275	2	
STAT3	P40763	0.102652	3	
PIK3CA	P42336	0.099376	4	
HDAC1	Q13547	0.098429	5	Yes
CDK1	P06493	0.054980	6	
HDAC6	Q9UBN7	0.052027	7	Yes
INSR	P06213	0.048892	8	
JAK2	O60674	0.045440	9	
CDK9	P50750	0.042186	10	
HDAC3	O15379	0.041809	11	Yes
AURKB	Q96GD4	0.037413	12	
XPO1	O14980	0.036882	13	
HDAC2	Q92769	0.030245	14	Yes
HDAC8	Q9BY41	0.025831	15	Yes
PIK3CB	P42338	0.021761	16	
AR	P10275	0.021657	17	
AURKA	O14965	0.021489	18	
PRKDC	P78527	0.020497	19	
HDAC5	Q9UQL6	0.020139	20	Yes
CDK4	P11802	0.013559	21	
CDK2	P24941	0.012451	22	
HDAC9	Q9UKV0	0.009975	23	Yes
PTK2	Q05397	0.009922	24	
PRKACA	P17612	0.009497	25	
PIK3CG	P48736	0.008997	26	
H2BW2	P0C1H6	0.008718	27	
CDK7	P50613	0.008671	28	
HDAC7	Q8WUI4	0.004601	29	Yes
WEE1	P30291	0.003032	30	
HDAC4	P56524	0.002895	31	Yes
FLT3	P36888	0.002813	32	
HSPA1A	P0DMV8	0.002491	33	
RPS2	P15880	0.001648	34	
PKIA	P61925	0.001107	35	
HDAC11	Q96DB2	0.000552	36	Yes
HDAC10	Q969S8	0.000468	37	Yes
BCL2L1	Q07817	0.000443	38	
ESR2	Q92731	0.000408	39	
ALK	Q9UM73	0.000185	40	
AXL	P30530	0.000000	41	
PIK3CD	O00329	0.000000	41	
PRKCG	P05129	0.000000	41	
PTK2B	Q14289	0.000000	41	

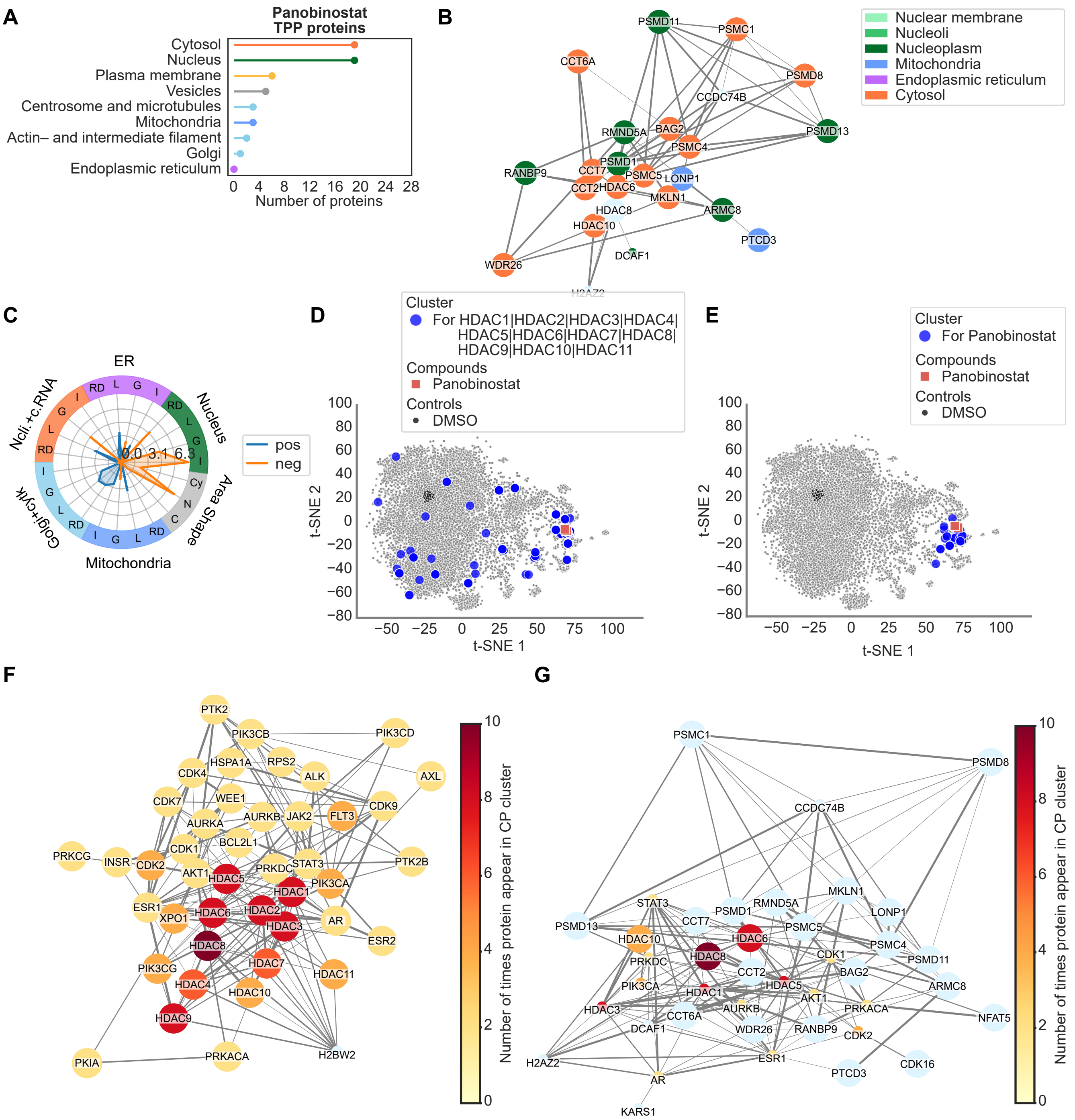
A
Panobinostat
TPP proteins
Cytosol
Nucleus
Plasma membrane
Vesicles
Centrosome and microtubules
Mitochondria
Actin– and intermediate filament
Golgi
Endoplasmic reticulum
Number of proteins
B
Nuclear membrane
Nucleoli
Nucleoplasm
Mitochondria
Endoplasmic reticulum
Cytosol
C
ER
Nucleus
Area Shape
Mitochondria
Golgi+cytk
Ncli.+c.RNA
pos
neg
D
Cluster
For HDAC1|HDAC2|HDAC3|HDAC4|HDAC5|HDAC6|HDAC7|HDAC8|HDAC9|HDAC10|HDAC11
Compounds
Panobinostat
Controls
DMSO
t-SNE 1
t-SNE 2
E
Cluster
For Panobinostat
Compounds
Panobinostat
Controls
DMSO
t-SNE 1
t-SNE 2
F
Number of times protein appear in CP cluster
G
Number of times protein appear in CP cluster

Appendix Figure S8: **Thermal proteome profiling (TPP) and Cell Painting analysis of Panobinostat treated cells. A.** Lollipop chart for subcellular location of the 35 proteins found to be either stabilized or destabilized in TPP for Panobinostat. Subcellular location of each protein, based on immunohistochemistry and confocal microscopy, was retrieved from the Human Protein Atlas (proteinatlas.org). When a protein had more than one subcellular location assigned, that protein was included once per subcellular location in the graph. **B.** Physical protein-protein interaction (PPI) networks for the proteins found to be either stabilized or destabilized in TPP. PPI networks were retrieved from the STRING db. Nodes are colored by subcellular location (from Human Protein Atlas). Large nodes correspond to proteins detected by TPP, where small nodes are additionally added nodes from the STRING db during network retrieval. **C.** Radar chart for features in cell painting data for Panobinostat. Features were grouped into categories based on two criteria: (i) Cell Profiler module, i.e. Intensity (I), Correlation (C), Granularity (G), Location (L) and RadialDistribution (RD); and (ii) stains, i.e. Nucleus (Hoechst), ER (Concanavalin A), Nucleoli and cytoplasmic RNA (SYTO14), Golgi apparatus and F-actin cytoskeleton (WGA and Phalloidin) and Mitochondria (Mitotracker). Features were only consider for the object Cell, except for features from the stain for Nucleus, which was only considered in the object Nucleus. Additionally, area-shape related features were grouped by cell compartment, i.e. Cell (C), Cytoplasm (Cy) and Nucleus (N). **D.** t-SNE for the morphological features in the SPECS cell painting data on U2OS cells. Blue dots show the location of all cells treated with compounds sharing at least one target with Panobinostat (RAF1 or BRAF). **E.** t-SNE for CP SPECS data showing Panobinostat (red) and a cluster of similar compounds (blue) identified using the consensus clustering algorithm SC3s on 13 principal components. K for the K-mean clustering algorithm was varied between 100 and 180. **F.** Physical PPI network for known protein targets of the compounds identified in the cluster in F. Panobinostat was blinded from the compound list before retrieving known targets. Network is colored by the number of times a protein target is present within the cluster. Large nodes indicate proteins also detected in TPP. **G.** Physical PPI network combining proteins identified in TPP and CP cluster, after applying a betweeness centrality calculation on the nodes from CP clustering. The number of CP supplied nodes to keep was chosen as the minimum number of nodes needed to maximize connection between the TPP identified proteins. The nodes are colored by number of times a protein target is present within the cluster in E.

Appendix Table S21: **Top 10 proteins with highest betweenness centrality score (BCS) for Panobinostat.**

Uniprot ID	Gene name	Protein name	BCS	Count in CP cluster
Q9UQL6 [2]	HDAC5	Histone deacetylase 5	0.13	8
P03372 [2]	ESR1	Estrogen receptor	0.11	2
P62195	PSMC5	26S proteasome regulatory subunit 8	0.10	0
Q9UBN7 [1]	HDAC6	Histone deacetylase 6	0.08	8
Q13547 [1 2]	HDAC1	Histone deacetylase 1	0.07	8
P10275 [2]	AR	Androgen receptor	0.06	2
P78527 [2]	PRKDC	DNA-dependent protein kinase catalytic subunit	0.05	2
P24941 [2]	CDK2	Cyclin-dependent kinase 2	0.05	4
P31749 [2]	AKT1	RAC-alpha serine/threonine-protein kinase	0.05	2
O15379 [1 2]	HDAC3	Histone deacetylase 3	0.04	8

[1]Known target for Panibinostat.

[2]Added from CP cluster

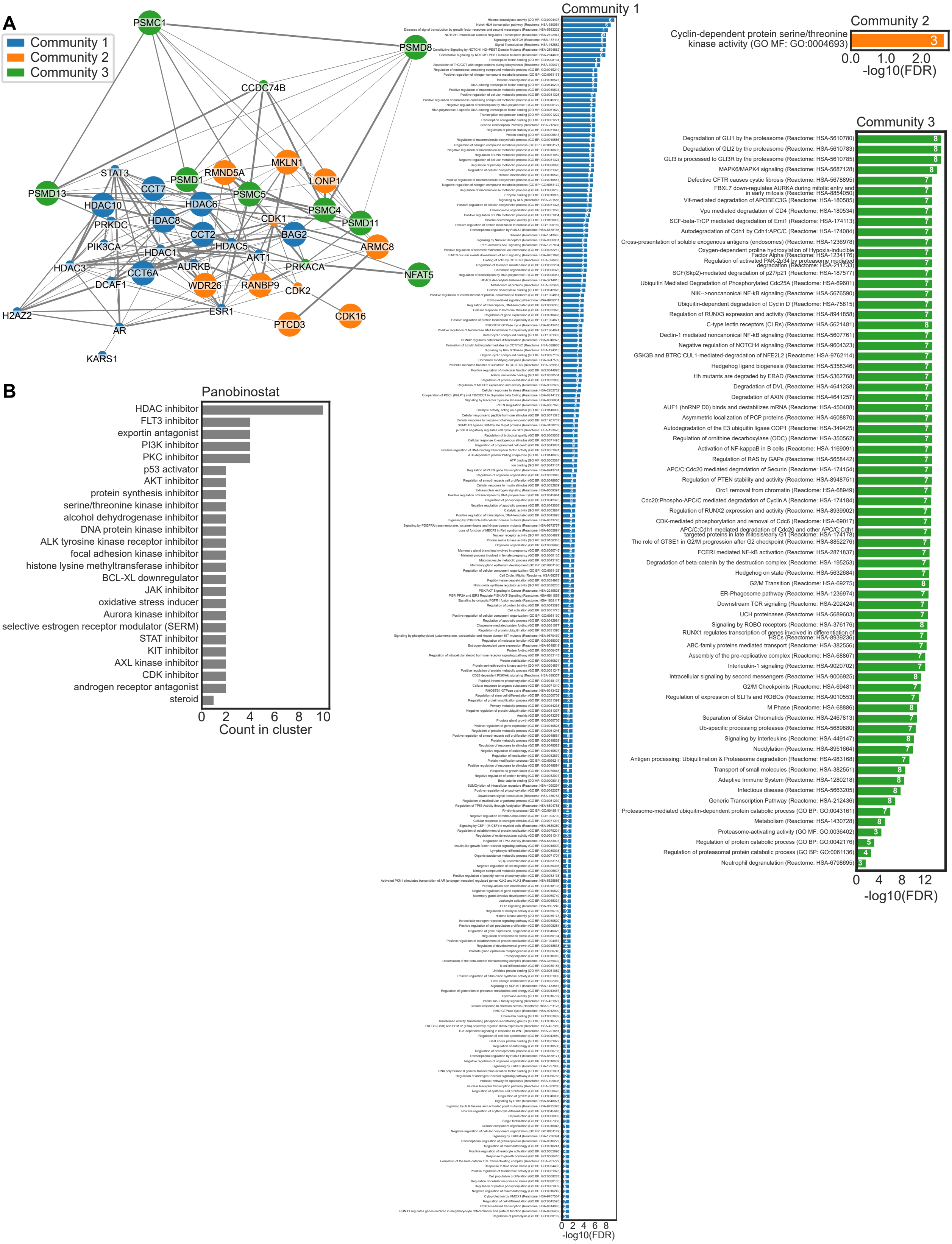

A
Community 1
Community 2
Community 3
PSMC1
PSMD8
CCDC74B
STAT3
PSMD1
RMND5A
MKLN1
LONP1
PSMD13
CCT7
PSMC5
HDAC10
HDAC6
PSMC4
PRKDC
HDAC8
CDK1
PSMD11
CCT2
BAG2
PIK3CA
HDAC5
ARMC8
HDAC1
AKT1
HDAC3
CCT6A
AURKB
PRKACA
NFAT5
DCAF1
WDR26
RANBP9
CDK2
ESR1
CDK16
H2AZ2
AR
PTCD3
KARS1
B
Panobinostat
HDAC inhibitor
FLT3 inhibitor
exportin antagonist
PI3K inhibitor
PKC inhibitor
p53 activator
AKT inhibitor
protein synthesis inhibitor
serine/threonine kinase inhibitor
alcohol dehydrogenase inhibitor
DNA protein kinase inhibitor
ALK tyrosine kinase receptor inhibitor
focal adhesion kinase inhibitor
histone lysine methyltransferase inhibitor
BCL-XL downregulator
JAK inhibitor
oxidative stress inducer
Aurora kinase inhibitor
selective estrogen receptor modulator (SERM)
STAT inhibitor
KIT inhibitor
AXL kinase inhibitor
CDK inhibitor
androgen receptor antagonist
steroid
0 2 4 6 8 10
Count in cluster
Community 1
0 2 4 6 8
-log10(FDR)
Community 2
Cyclin-dependent protein serine/threonine kinase activity (GO MF: GO:0004693)
3
0.0 1.0 2.0
-log10(FDR)
Community 3
Degradation of GLI1 by the proteasome (Reactome: HSA-5610780)
Degradation of GLI2 by the proteasome (Reactome: HSA-5610783)
GLI3 is processed to GLI3R by the proteasome (Reactome: HSA-5610785)
MAPK6/MAPK4 signaling (Reactome: HSA-5687128)
Defective CFTR causes cystic fibrosis (Reactome: HSA-5678895)
FBXL7 down-regulates AURKA during mitotic entry and in early mitosis (Reactome: HSA-8854050)
Vif-mediated degradation of APOBEC3G (Reactome: HSA-180585)
Vpu mediated degradation of CD4 (Reactome: HSA-180534)
SCF-beta-TrCP mediated degradation of Emi1 (Reactome: HSA-174113)
Autodegradation of Cdh1 by Cdh1:APC/C (Reactome: HSA-174084)
Cross-presentation of soluble exogenous antigens (endosomes) (Reactome: HSA-1236978)
Oxygen-dependent proline hydroxylation of Hypoxia-inducible Factor Alpha (Reactome: HSA-1234176)
Regulation of activated PAK-2p34 by proteasome mediated degradation (Reactome: HSA-211733)
SCF(Skp2)-mediated degradation of p27/p21 (Reactome: HSA-187577)
Ubiquitin Mediated Degradation of Phosphorylated Cdc25A (Reactome: HSA-69601)
NIK-->noncanonical NF-kB signaling (Reactome: HSA-5676590)
Ubiquitin-dependent degradation of Cyclin D (Reactome: HSA-75815)
Regulation of RUNX3 expression and activity (Reactome: HSA-8941858)
C-type lectin receptors (CLRs) (Reactome: HSA-5621481)
Dectin-1 mediated noncanonical NF-kB signaling (Reactome: HSA-5607761)
Negative regulation of NOTCH4 signaling (Reactome: HSA-9604323)
GSK3B and BTRC:CUL1-mediated-degradation of NFE2L2 (Reactome: HSA-9762114)
Hedgehog ligand biogenesis (Reactome: HSA-5358346)
Hh mutants are degraded by ERAD (Reactome: HSA-5362768)
Degradation of DVL (Reactome: HSA-4641258)
Degradation of AXIN (Reactome: HSA-4641257)
AUF1 (hnRNP D0) binds and destabilizes mRNA (Reactome: HSA-450408)
Asymmetric localization of PCP proteins (Reactome: HSA-4608870)
Autodegradation of the E3 ubiquitin ligase COP1 (Reactome: HSA-349425)
Regulation of ornithine decarboxylase (ODC) (Reactome: HSA-350562)
Activation of NF-kappaB in B cells (Reactome: HSA-1169091)
Regulation of RAS by GAPs (Reactome: HSA-5658442)
APC/C:Cdc20 mediated degradation of Securin (Reactome: HSA-174154)
Regulation of PTEN stability and activity (Reactome: HSA-8948751)
Orc1 removal from chromatin (Reactome: HSA-68949)
Cdc20:Phospho-APC/C mediated degradation of Cyclin A (Reactome: HSA-174184)
Regulation of RUNX2 expression and activity (Reactome: HSA-8939902)
CDK-mediated phosphorylation and removal of Cdc6 (Reactome: HSA-69017)
APC/C:Cdh1 mediated degradation of Cdc20 and other APC/C:Cdh1 targeted proteins in late mitosis/early G1 (Reactome: HSA-174178)
The role of GTSE1 in G2/M progression after G2 checkpoint (Reactome: HSA-8852276)
FCERI mediated NF-kB activation (Reactome: HSA-2871837)
Degradation of beta-catenin by the destruction complex (Reactome: HSA-195253)
Hedgehog on state (Reactome: HSA-5632684)
G2/M Transition (Reactome: HSA-69275)
ER-Phagosome pathway (Reactome: HSA-1236974)
Downstream TCR signaling (Reactome: HSA-202424)
UCH proteinases (Reactome: HSA-5689603)
Signaling by ROBO receptors (Reactome: HSA-376176)
RUNX1 regulates transcription of genes involved in differentiation of HSCs (Reactome: HSA-8939236)
ABC-family proteins mediated transport (Reactome: HSA-382556)
Assembly of the pre-replicative complex (Reactome: HSA-68867)
Interleukin-1 signaling (Reactome: HSA-9020702)
Intracellular signaling by second messengers (Reactome: HSA-9006925)
G2/M Checkpoints (Reactome: HSA-69481)
Regulation of expression of SLITs and ROBOs (Reactome: HSA-9010553)
M Phase (Reactome: HSA-68886)
Separation of Sister Chromatids (Reactome: HSA-2467813)
Ub-specific processing proteases (Reactome: HSA-5689880)
Signaling by Interleukins (Reactome: HSA-449147)
Neddylation (Reactome: HSA-8951664)
Antigen processing: Ubiquitination & Proteasome degradation (Reactome: HSA-983168)
Transport of small molecules (Reactome: HSA-382551)
Adaptive Immune System (Reactome: HSA-1280218)
Infectious disease (Reactome: HSA-5663205)
Generic Transcription Pathway (Reactome: HSA-212436)
Proteasome-mediated ubiquitin-dependent protein catabolic process (GO BP: GO:0043161)
Metabolism (Reactome: HSA-1430728)
Proteasome-activating activity (GO MF: GO:0036402)
Regulation of protein catabolic process (GO BP: GO:0042176)
Regulation of proteasomal protein catabolic process (GO BP: GO:0061136)
Neutrophil degranulation (Reactome: HSA-6798695)
0 4 8 12
-log10(FDR)

Appendix Figure S9: **Gene Onthology (GO) and Reactome enrichment analysis of Panobinostat PPI network graph. A.** Physical PPI network (same as in figure S6G) labeled by community. Communitites were identified using Clauset-Newman-Moore greedy modularity maximization. GO Molecular function (GO MF), GO Biological Process (GO BP), and Reactome pathway enrichment analysis for each community in graph are also shown as bar charts. Only enrichment with FDR ≤ 0.05 are shown. **B.** Prevalence (count) of mechanism of action (MoA) annotations for compounds in CP cluster for Panobinostat (shown in figure S6E)

Appendix Table S22: **Significantly stabilized/destabilized proteins in ITDR TPP upon treatment with Crizotinib.**

Gene names	Uniprot ID	Stabilized/Destabilized rep 1	Stabilized/Destabilized rep 2
Ataxin-10	NA	NA	stabilized
PICALM	L8ECD8	stabilized	NA
EZR	Q6NUR7	NA	stabilized
AASDHPPT	E9PNF3	stabilized	NA
YWHAB	Q4VY20	NA	stabilized
GLB1	F8WF40	NA	destabilized
TXNL1	V9HW51	NA	stabilized
HSPA4L	E9PDE8	destabilized	NA
PAIP1	H0YA44	NA	stabilized
MAPRE1	A2VCR0	NA	stabilized
CD2AP	Q9Y5K6	NA	stabilized
DCTN2	H0YI98	NA	stabilized
SERPINH1	H0YEP8	stabilized	NA
RCC2	A5PLK7	NA	stabilized
PPME1	Q9Y570	NA	stabilized
ARD1A	Q6P4J0	stabilized	NA
FAM49B	Q9NUQ9	stabilized	NA
ST13	Q9P1I4	NA	stabilized
CAP1	Q5T0S3	NA	stabilized
CACYBP	Q6NVY0	NA	stabilized
CIAPIN1	H3BV90	NA	stabilized
PTGES3	B4DDC6	NA	stabilized
TBCC	Q15814	NA	stabilized
LAMP1	P11279	NA	stabilized
RNH1	H0YCR7	stabilized	stabilized
WDR1	Q53GN4	stabilized	stabilized
NUCB2	Q2L696	NA	stabilized
PCMT1	H7C4X2	NA	stabilized
UBE2M	M0QYI6	NA	stabilized
PCBP1	Q53SS8	NA	stabilized
UBA2	U3KQ93	NA	stabilized
NUDC	A0A0A0MSU9	stabilized	NA
EPS15L1	M0R3I1	stabilized	NA
FERMT3	H0YFT5	NA	stabilized
PUF60	H0YEM1	NA	stabilized
FAM50A	B0S8I6	NA	stabilized
ANXA2	H0YNP5	NA	stabilized
CLIC1	Q5SRT3	NA	stabilized
EIF3G	Q6IAM0	NA	stabilized
LMNB1	Q6DC98	NA	stabilized
ATP6V1E1	Q53Y06	NA	stabilized
GRB2	Q6ICN0	NA	stabilized
CBFB	J3KTD8	NA	stabilized
COPE	M0R061	stabilized	NA
CTSB	Q8TAC7	NA	destabilized
HADH	J3KR89	stabilized	NA
TXNDC4	Q9BS26	NA	stabilized
ERH	G3V279	NA	stabilized
TPR	Q9UE33	NA	stabilized
AP1G1	Q9BTR5	NA	stabilized
API5	H0YER7	NA	stabilized
UBE2K	L8E874	NA	stabilized
SERPINB1	V9HWH1	stabilized	NA
NEU1	Q6Q4G9	NA	destabilized
AKR7A2	V9HWA2	stabilized	stabilized
GLOD4	K7ENF2	NA	stabilized
ATP6V1H	H0YB41	stabilized	NA
PPIF	R4GN99	stabilized	NA

Appendix Table S23: **Betweenness centrality scores (BSC) and ranks for only TPP data, Crizotinib.**

Gene name	Uniprot ID	BSC	Rank	Known target
UBE2M	P61081	0.482399	1	
GRB2	P62993	0.342786	2	
ANXA2	P07355	0.264396	3	
EPS15L1	Q9UBC2	0.198068	4	
PICALM	Q13492	0.124638	5	
YWHAB	P31946	0.119726	6	
EZR	P15311	0.116779	7	
CLIC1	O00299	0.107874	8	
PCBP1	Q15365	0.088905	9	
HSPA4L	O95757	0.088808	10	
ATP6V1E1	P36543	0.085990	11	
PTGES3	Q15185	0.078986	12	
PPME1	Q9Y570	0.065926	13	
GLOD4	Q9HC38	0.053623	14	
MAPRE1	Q15691	0.051401	15	
WDR1	O75083	0.045974	16	
DCTN2	Q13561	0.044928	17	
COPE	O14579	0.043478	18	
SERPINH1	P50454	0.043478	19	
CACYBP	Q9HB71	0.040821	20	
UBE2K	P61086	0.016425	21	
UBA2	Q9UBT2	0.015620	22	
CD2AP	Q9Y5K6	0.012963	23	
PUF60	Q9UHX1	0.010741	24	
EIF3G	O75821	0.008454	25	
PAIP1	Q9H074	0.007085	26	
NUDC	Q9Y266	0.006280	27	
ERH	P84090	0.002899	28	
FAM49B	Q9NUQ9	0.002528	29	
LMNB1	P20700	0.000966	30	
PCMT1	P22061	0.000000	31	
TXNL1	O43396	0.000000	31	
ST13	P50502	0.000000	31	
CBFB	Q13951	0.000000	31	
GAPT	Q8N292	0.000000	31	
ERP44	Q9BS26	0.000000	31	
NAA10	P41227	0.000000	31	
AP1G1	O43747	0.000000	31	
LAMP1	P11279	0.000000	31	
RNH1	O60930	0.000000	31	
NUCB2	P80303	0.000000	31	
CTSB	P07858	0.000000	31	
CAP1	Q01518	0.000000	31	
AKR7A2	O43488	0.000000	31	
ATP6V1H	Q9UI12	0.000000	31	
PPIF	P30405	0.000000	31	
ATXN10	Q9UBB4	0.000000	31	

Appendix Table S24: **Betweenness centrality scores (BSC) and ranks for only CP cluster data, Crizotinib.**

Gene name	Uniprot ID	BSC	Rank	Known target
ABL1	P00519	0.733333	1	Off-target
BCR	P11274	0.533333	2	Off-target
LYN	P07948	0.333333	3	
RAC1	P63000	0.333333	4	
SPESP1	Q6UW49	0.000000	5	
PDGFRB	P09619	0.000000	5	
RAC3	P60763	0.000000	5	

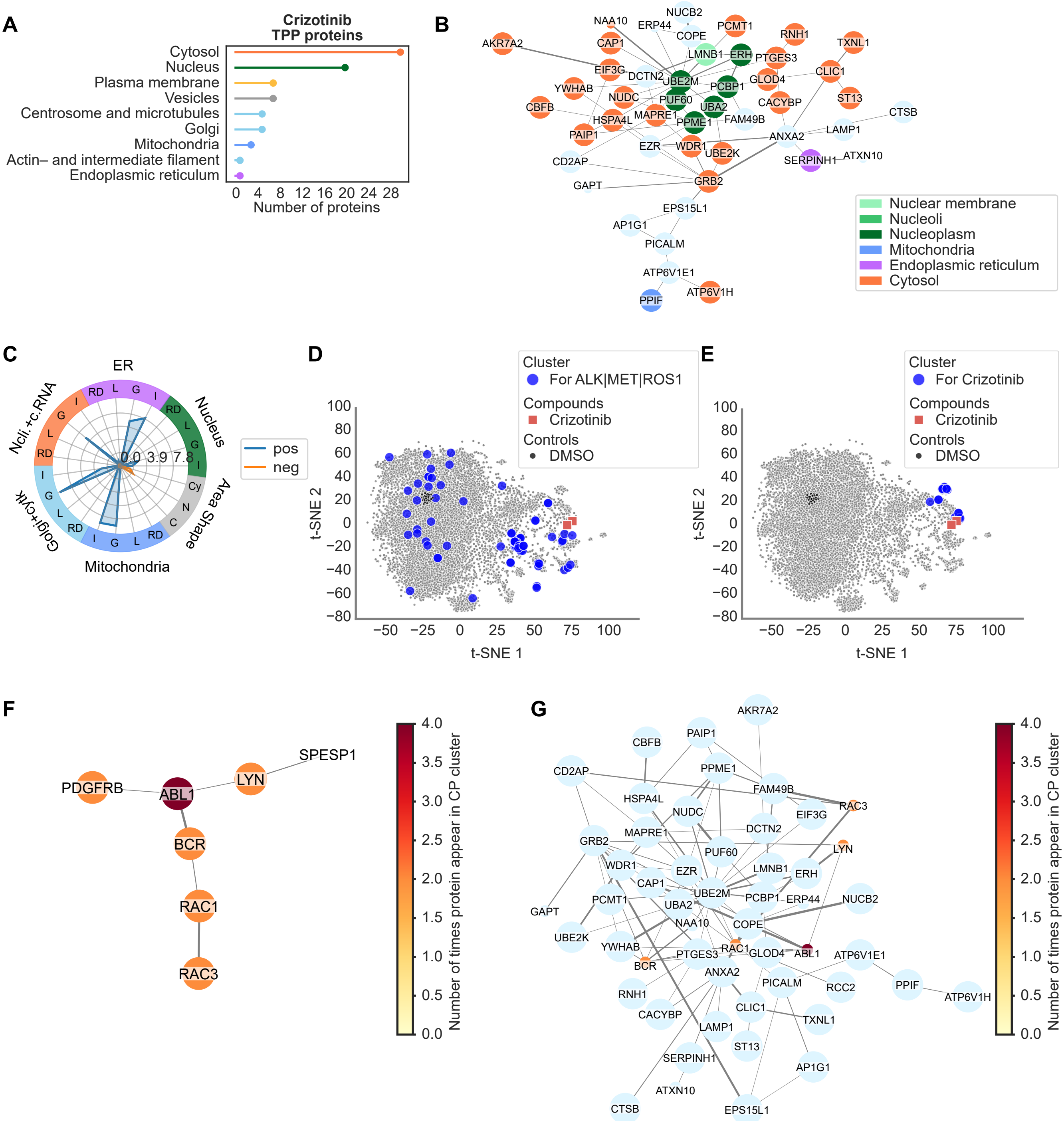
A
Crizotinib
TPP proteins
Cytosol
Nucleus
Plasma membrane
Vesicles
Centrosome and microtubules
Golgi
Mitochondria
Actin– and intermediate filament
Endoplasmic reticulum
Number of proteins
B
Nuclear membrane
Nucleoli
Nucleoplasm
Mitochondria
Endoplasmic reticulum
Cytosol
C
ER
Nucleus
Area Shape
Mitochondria
Golgi+cytk
Ncli.+c.RNA
pos
neg
D
Cluster
For ALK|MET|ROS1
Compounds
Crizotinib
Controls
DMSO
t-SNE 1
t-SNE 2
E
Cluster
For Crizotinib
Compounds
Crizotinib
Controls
DMSO
t-SNE 1
t-SNE 2
F
PDGFRB
ABL1
LYN
SPESP1
BCR
RAC1
RAC3
Number of times protein appear in CP cluster
G
Number of times protein appear in CP cluster

Appendix Figure S10: **Thermal proteome profiling (TPP) and Cell Painting analysis of Crizotinib treated cells.** **A.** Lollipop chart for subcellular location of the 58 proteins found to be either stabilized or destabilized in TPP for Crizotinib. Subcellular location of each protein, based on immunohistochemistry and confocal microscopy, was retrieved from the Human Protein Atlas (proteinatlas.org). When a protein had more than one subcellular location assigned, that protein was included once per subcellular location in the graph. **B.** Physical protein-protein interaction (PPI) networks for the proteins found to be either stabilized or destabilized in TPP. PPI networks were retrieved from the STRING db. Nodes are colored by subcellular location (from Human Protein Atlas). Large nodes correspond to proteins detected by TPP, where small nodes are additionally added nodes from the STRING db during network retrieval. **C.** Radar chart for features in cell painting data for Crizotinib. Features were grouped into categories based on two criteria: (i) Cell Profiler module, i.e. Intensity (I), Correlation (C), Granularity (G), Location (L) and RadialDistribution (RD); and (ii) stains, i.e. Nucleus (Hoechst), ER (Concanavalin A), Nucleoli and cytoplasmic RNA (SYTO14), Golgi apparatus and F-actin cytoskeleton (WGA and Phalloidin) and Mitochondria (Mitotracker). Features were only consider for the object Cell, except for features from the stain for Nucleus, which was only considered in the object Nucleus. Additionally, area-shape related features were grouped by cell compartment, i.e. Cell (C), Cytoplasm (Cy) and Nucleus (N). **D.** t-SNE for the morphological features in the SPECS cell painting data on U2OS cells. Blue dots show the location of all cells treated with compounds sharing at least one target with Crizotinib (RAF1 or BRAF). **E.** t-SNE for CP SPECS data showing Crizotinib (red) and a cluster of similar compounds (blue) identified using the consensus clustering algorithm SC3s on 13 principal components. K for the K-mean clustering algorithm was varied between 100 and 180. **F.** Physical PPI network for known protein targets of the compounds identified in the cluster in F. Crizotinib was blinded from the compound list before retrieving known targets. Network is colored by the number of times a protein target is present within the cluster. Large nodes indicate proteins also detected in TPP. **G.** Physical PPI network combining proteins identified in TPP and CP cluster, after applying a betweeness centrality calculation on the nodes from CP clustering. The number of CP supplied nodes to keep was chosen as the minimum number of nodes needed to maximize connection between the TPP identified proteins. The nodes are colored by number of times a protein target is present within the cluster in E.

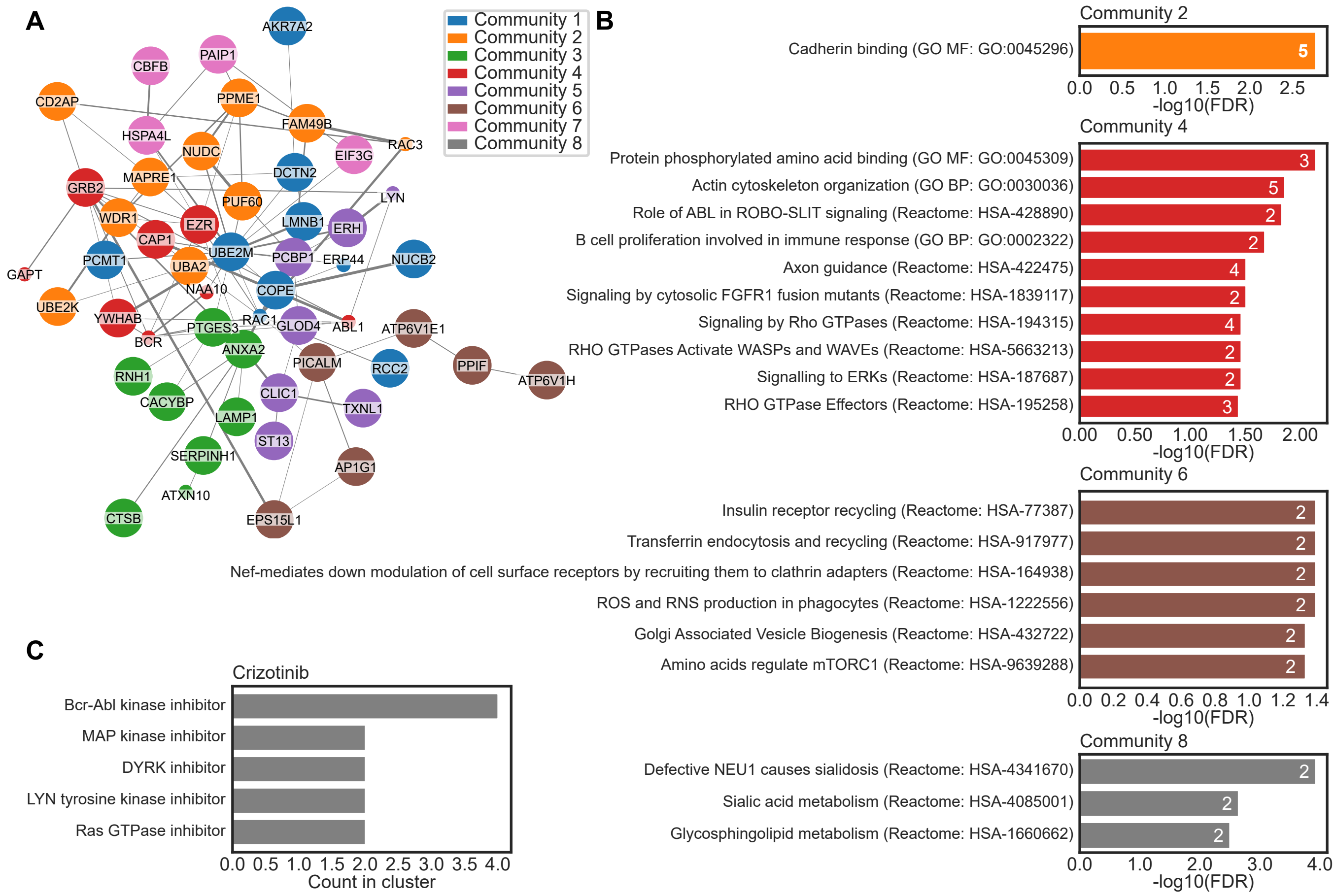

Appendix Figure S11: **Gene Onthology (GO) and Reactome enrichment analysis of Crizotinib PPI network graph. A.** Physical PPI network (same as in figure S8G) labeled by community. Communitites were identified using Clauset-Newman-Moore greedy modularity maximization. **B.** GO Molecular function (GO MF), GO Biological Process (GO BP), and Reactome pathway enrichment analysis for each community in A. Only enrichment with FDR ≤ 0.05 are shown. **C.** Prevalence (count) of mechanism of action (MoA) annotations for compounds in CP cluster for Crizotinib (shown in figure S8E)

Appendix Table S25: Top 10 proteins with highest betweenness centrality score (BCS) for Crizotinib.

Uniprot ID	Gene name	Protein name	BCS	Count in CP cluster
P61081	UBE2M	NEDD8-conjugating enzyme Ubc12	0.37	0
P63000 [3]	RAC1	Ras-related C3 botulinum toxin substrate 1	0.32	2
P07355	ANXA2	Annexin A2	0.25	0
P62993 [2]	GRB2	Growth factor receptor-bound protein 2	0.18	0
Q13492	PICALM	Phosphatidylinositol-binding clathrin assembly protein	0.14	0
O00299	CLIC1	Chloride intracellular channel protein 1	0.09	0
P15311	EZR	Ezrin	0.08	0
P36543	ATP6V1E1	V-type proton ATPase subunit E 1	0.08	0
Q15185	PTGES3	Prostaglandin E synthase 3	0.06	0
O95757	HSPA4L	Heat shock 70 kDa protein 4L	0.06	0

[1]Known target for Crizotinib.

[2]Interacts with MET

[3]Added from CP cluster

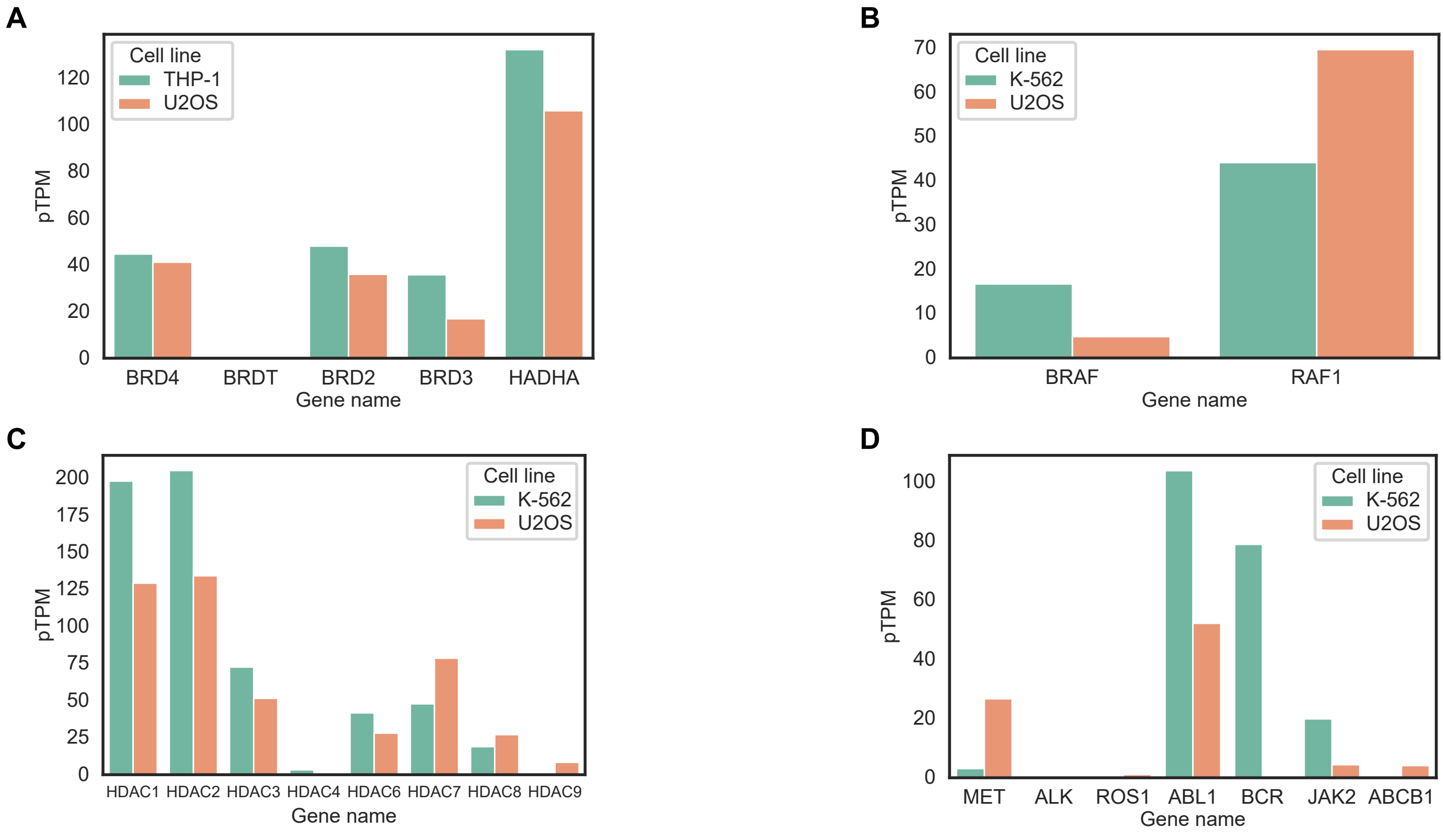

Appendix Figure S12: mRNA expression of known target and off-target proteins in the cell lines used for TPP and CP experiments for: A. (+)-JQ1 and I-BET151 **B.** Vemurafenib **C.** Panobinostat **D.** Crizotinib. mRNA expression profiles were retrieved from the Human Protein Atlas (www.proteinatlas.org)

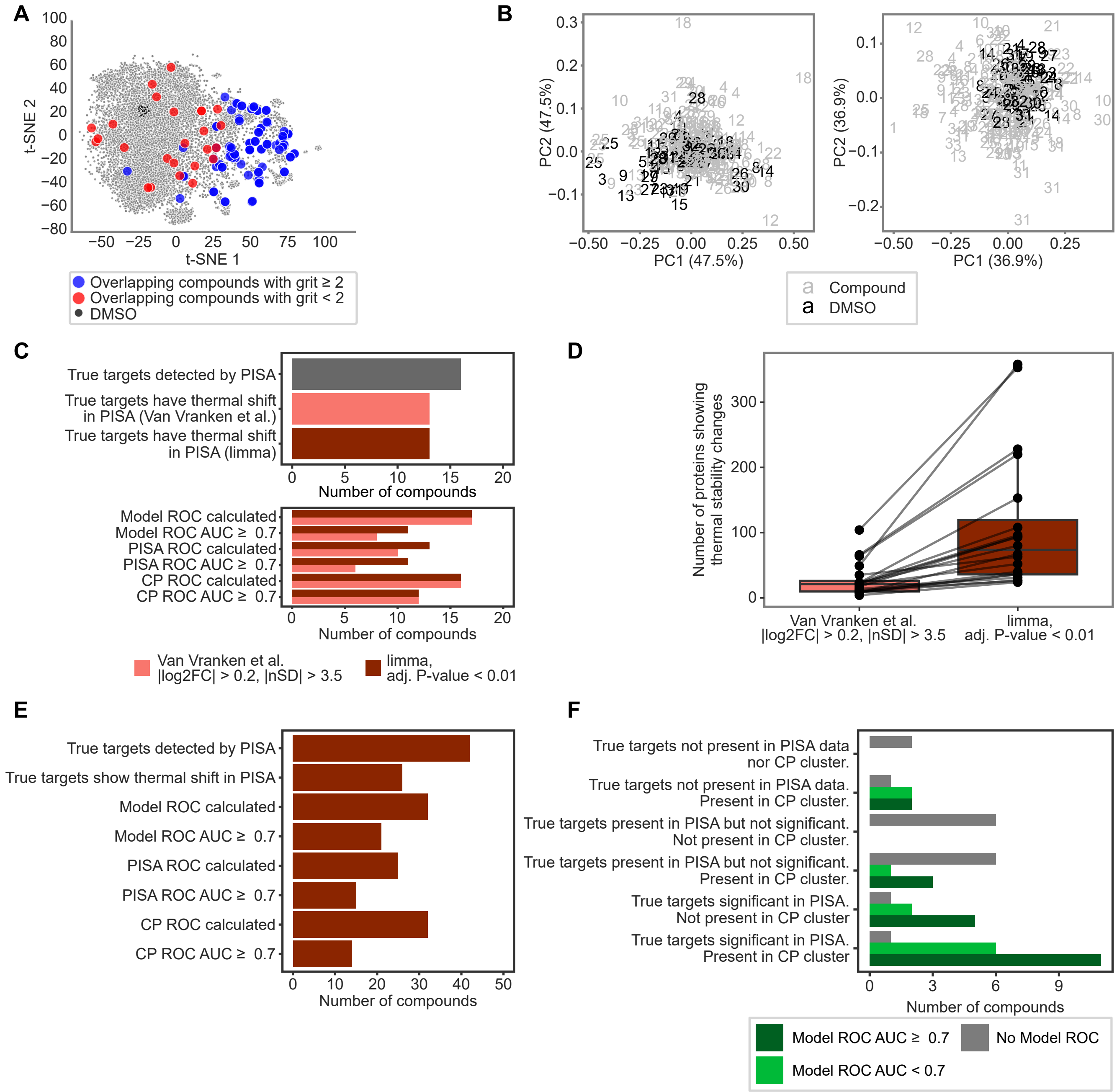
A
t-SNE 2
t-SNE 1
Overlapping compounds with grit ≥ 2
Overlapping compounds with grit < 2
DMSO
B
PC2 (47.5%)
PC1 (47.5%)
PC2 (36.9%)
PC1 (36.9%)
Compound
DMSO
C
True targets detected by PISA
True targets have thermal shift in PISA (Van Vranken et al.)
True targets have thermal shift in PISA (limma)
Number of compounds
Model ROC calculated
Model ROC AUC ≥ 0.7
PISA ROC calculated
PISA ROC AUC ≥ 0.7
CP ROC calculated
CP ROC AUC ≥ 0.7
Number of compounds
Van Vranken et al. |log2FC| > 0.2, |nSD| > 3.5
limma, adj. P-value < 0.01
D
Number of proteins showing thermal stability changes
Van Vranken et al. |log2FC| > 0.2, |nSD| > 3.5
limma, adj. P-value < 0.01
E
True targets detected by PISA
True targets show thermal shift in PISA
Model ROC calculated
Model ROC AUC ≥ 0.7
PISA ROC calculated
PISA ROC AUC ≥ 0.7
CP ROC calculated
CP ROC AUC ≥ 0.7
Number of compounds
F
True targets not present in PISA data nor CP cluster.
True targets not present in PISA data. Present in CP cluster.
True targets present in PISA but not significant. Not present in CP cluster.
True targets present in PISA but not significant. Present in CP cluster.
True targets significant in PISA. Not present in CP cluster
True targets significant in PISA. Present in CP cluster
Number of compounds
Model ROC AUC ≥ 0.7
No Model ROC
Model ROC AUC < 0.7

Appendix Figure S13: **Validation of workflow performance using a high-throughput PISA data set. A.** Of the 64 compounds in the PISA data set [1] which were also present in the CP data, 54 had a grit score ≥ 2. Of these, 49 compounds were selected for evaluation of our workflow. **B.** Principal component analysis of PISA data before (left) and after (right) accounting for batch effect using the R-package `limma` [2]. Compounds are shown in grey and the DMSO negative controls are shown in black. Numbers correspond to 32 experimental batches. `limma` successfully reduces batch effects in the PISA data. For downstream analysis, all DMSO controls were assumed to be derived from the same population after accounting for batch effects, and `limma` modified t-test was calculated for each compound duplicate compared to all DMSO controls across the whole PISA assay. **C, D** Comparison of workflow performance for the first 20 compounds when thermal shifts in PISA data have been calculated using either the empirical filtering described by Van Vranken et al. [1] or using `limma` modified t-test. For each compound, true targets were defined as the complete list of known targets annotated in the PISA publication [1]. **C.** The number of compounds where at least one true target had a significant thermal shift did not differ between the Van Vranken et al. empirical filtering and `limma` (top). However, detecting thermal stability shifts with `limma` resulted in an improved target prediction with our workflow as illustrated by an increased ROC AUC of the final combined model (bottom). **D.** `limma` identified more thermal stability changes per compound compared to the Van Vranken et al. empirical filtering (a median of 73.5 versus 21 shifts). **E, F** Workflow performance on true target prediction for all 49 compounds showed that combining PISA and CP data resulted in more compounds having a ROC AUC ≥ 0.7 compared to when analysing only PISA or only CP data. **F.** The combined model did not include any of the true targets for 17 compounds. For eight of these, true targets were not present in CP cluster and either not detected by PISA at all or detected but not displaying significant thermal stability shifts, which makes it impossible for our workflow to detect these targets.

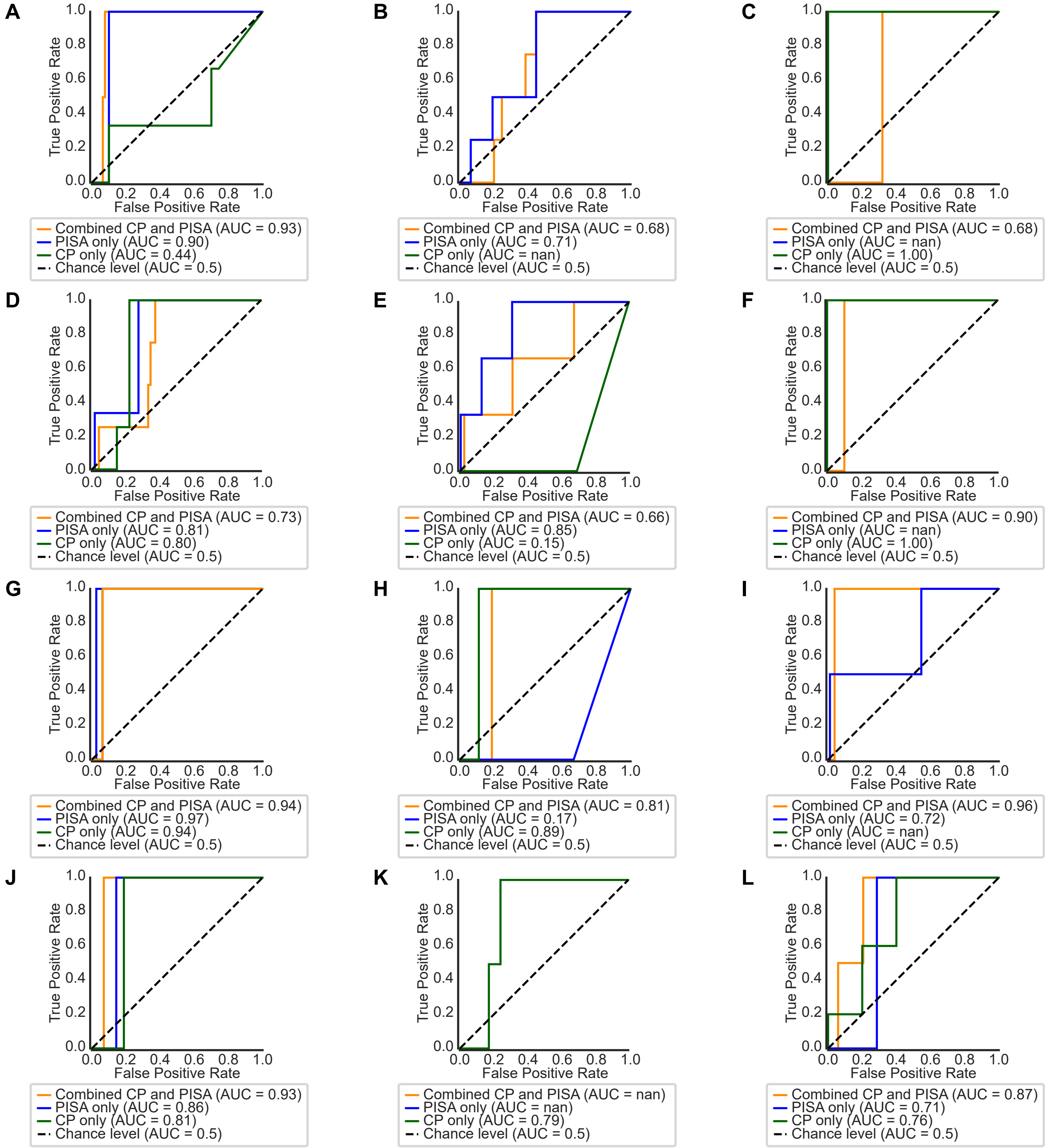

Appendix Figure S14: Receiver operating characteristic (ROC) curves for detection of true targets using our workflow on compounds 1–12 out of 40 compounds in the PISA data from Van Vranken et al [1] where targets were detected by at lest one method. A. Nilotinib **B.** Dasatinib **C.** Erlotinib **D.** Vorinostat **E.** BI 2536 **F.** Crizotinib **G.** Rucaparib (Camsylate) **H.** Doramapimod **I.** MK-2206 **J.** Alisertib **K.** Vemurafenib **L.** Pictilisib.

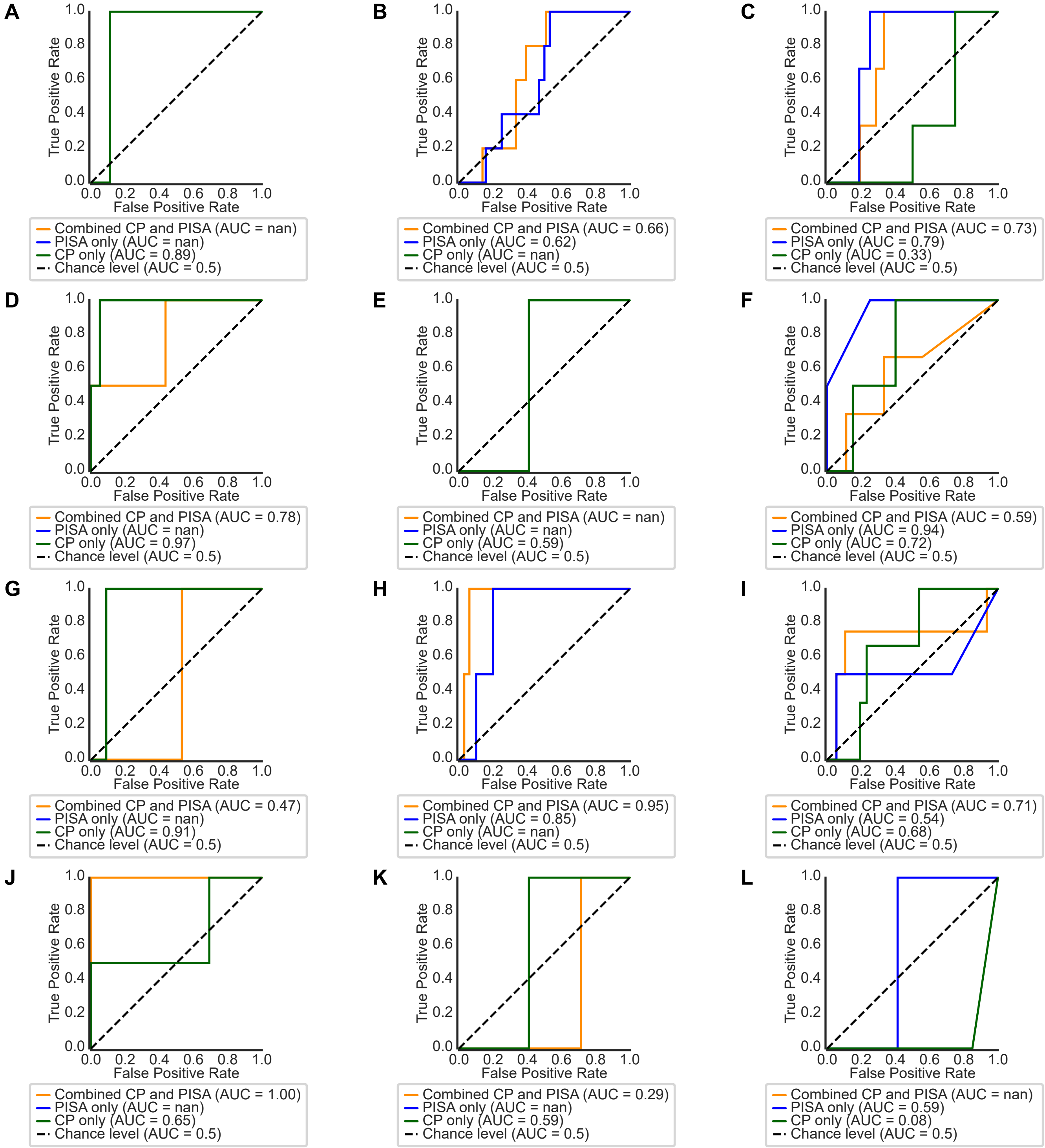

Appendix Figure S15: Receiver operating characteristic (ROC) curves for detection of true targets using our workflow on compounds 13–24 out of 40 compounds in the PISA data from Van Vranken et al [1] where targets were detected by at lest one method. A. Gandotinib **B.** Sotrastaurin **C.** Tubastatin A **D.** Lapatinib **E.** SB 525334 **F.** Tozertib **G.** Mubritinib **H.** Silmitisertib **I.** AZD-5438 **J.** CCT128930 **K.** SD-208 **L.** Ispinesib.

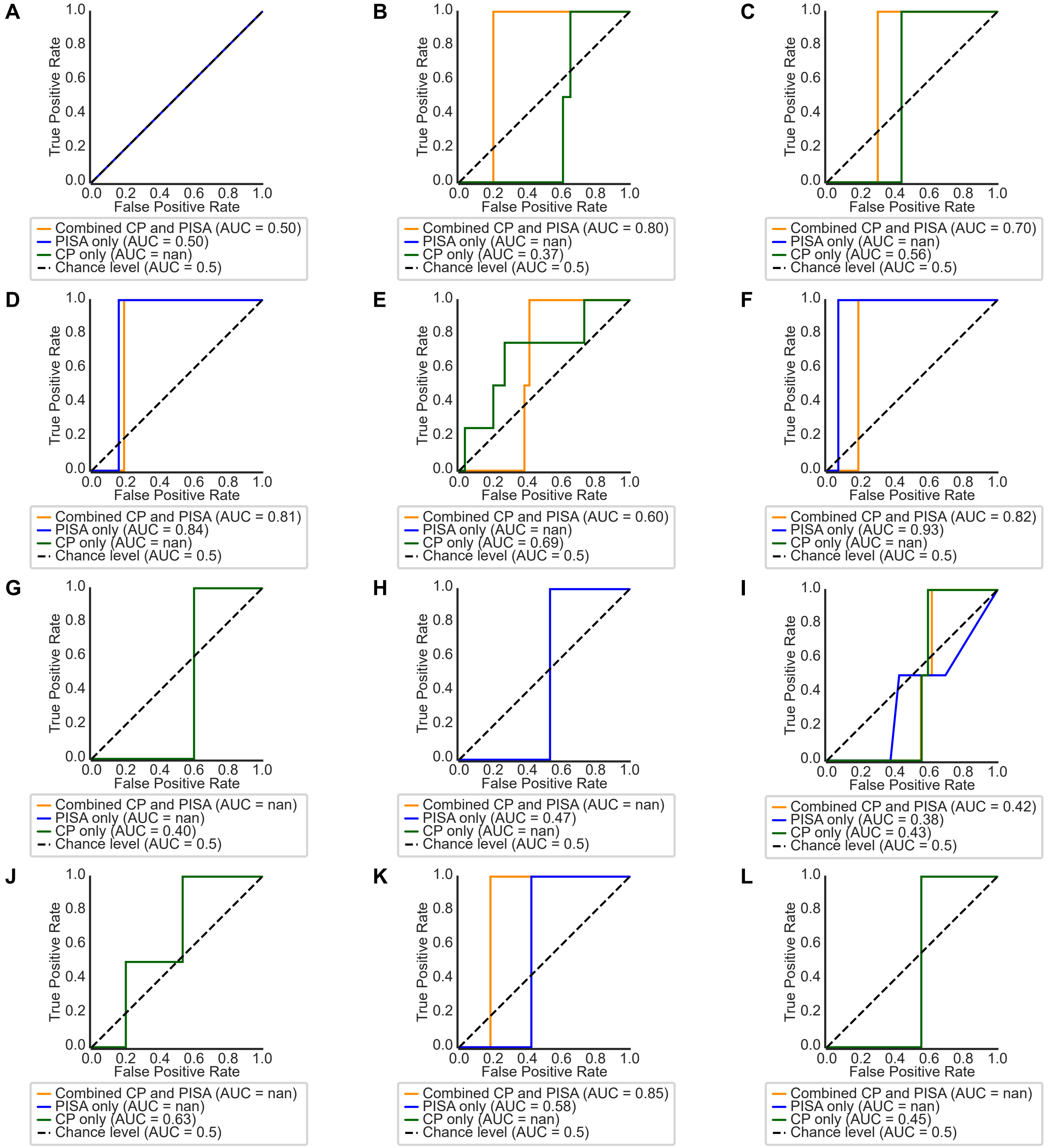

Appendix Figure S16: Receiver operating characteristic (ROC) curves for detection of true targets using our workflow on compounds 25–36 out of 40 compounds in the PISA data from Van Vranken et al [1] where targets were detected by at lest one method. A. GSK429286A **B.** AZ 628 **C.** TAK-285 **D.** NVP-TAE 226 **E.** Duvelisib **F.** GSK2334470 **G.** AZ20 **H.** PF-3758309 **I.** Y-33075 (dihydrochloride) **J.** AZD1152 **K.** Defactinib **L.** CC-401 (hydrochloride).

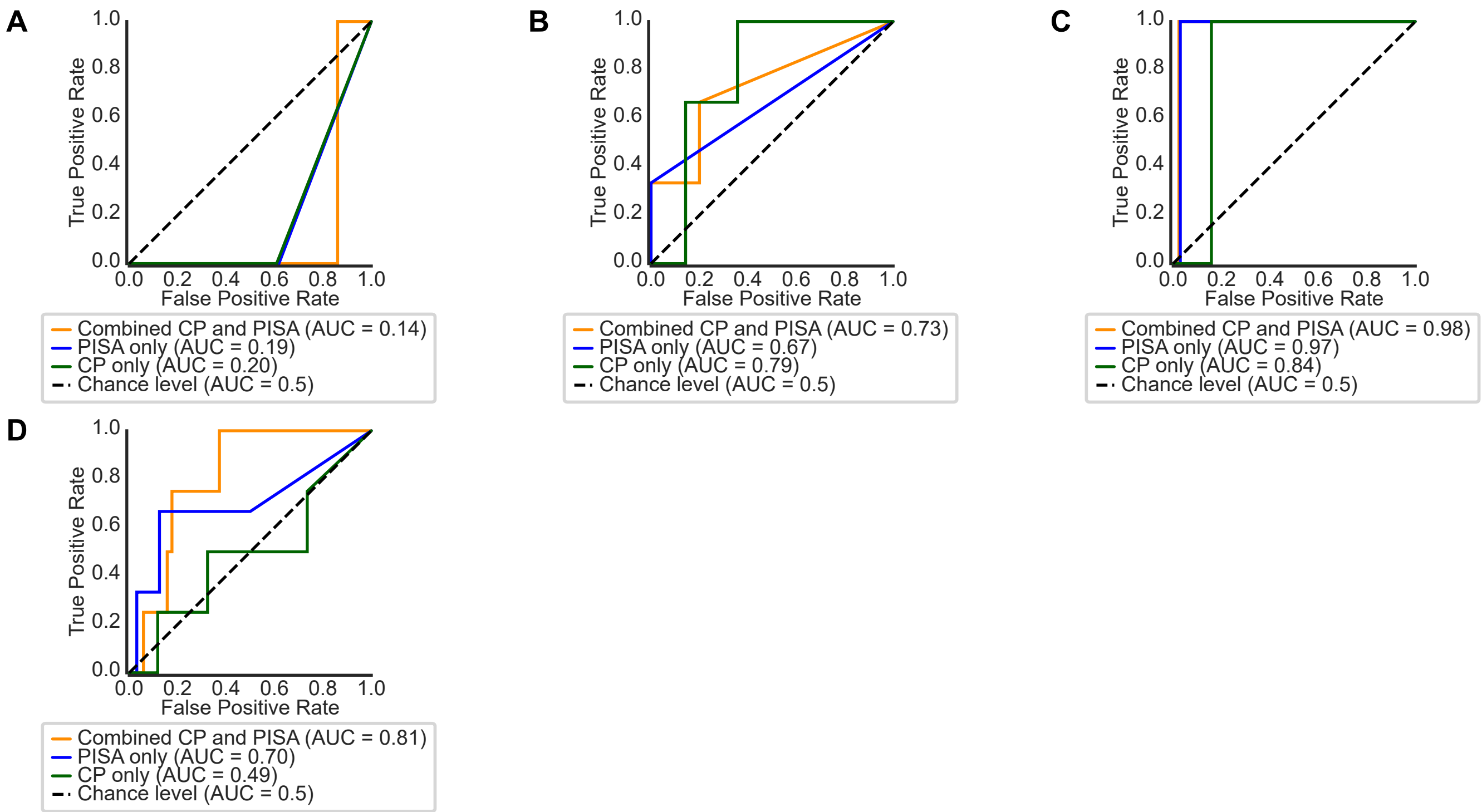

Appendix Figure S17: **Receiver operating characteristic (ROC) curves for detection of true targets using our workflow on compounds 37–40 out of 40 compounds in the PISA data from Van Vranken et al [1] where targets were detected by at lest one method. A.** Filanesib **B.** CPI-203 **C.** Ly2090314 **D.** Nexturastat A.

References

[1] J.G. Van Vranken, J. Li, J. Mintseris, T.Y. Wei, C.M. Sniezek, M. Gadzuk-Shea, S.P. Gygi, D.K. Schweppe, Large-scale characterization of drug mechanism of action using proteome-wide thermal shift assays. eLife **13**, RP95595 (2024). https://doi.org/10.7554/eLife.95595. URL https://doi.org/10.7554/eLife.95595

[2] M.E. Ritchie, B. Phipson, D. Wu, Y. Hu, C.W. Law, W. Shi, G.K. Smyth, limma powers differential expression analyses for rna-sequencing and microarray studies. Nucleic acids research **43**(7), e47–e47 (2015)
