# Supplementary material for: Cell painting and thermal proteome profiling for inference of drug targets and mechanism of action
Source: Mol Syst Biol. 2026 May 15;22(8):1270–91. doi: 10.1038/s44320-026-00214-9 (PMC13434728; doi:10.1038/s44320-026-00214-9)
Supplement: Supplementary file 3 [file 44320_2026_214_MOESM8_ESM.pdf]

Expanded View Figures

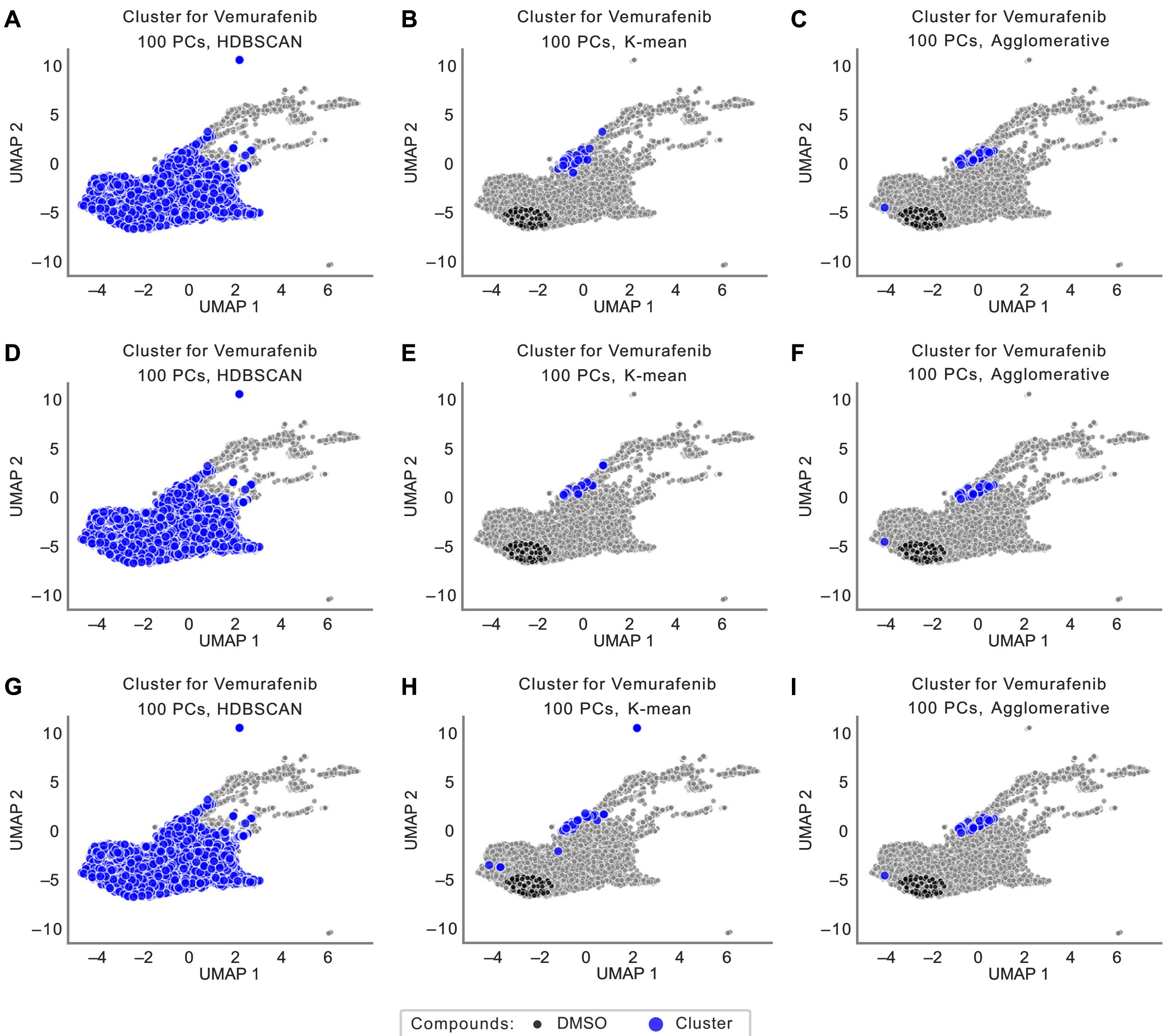

Fig. EV1. Comparison of clustering algorithms for CP data on the compound Vemurafenib.

UMAP representations of clustering Vemurafenib with (**A**, **D**, **G**) HDBSCAN clustering; (**B**, **E**, **H**) *k*-means clustering; (**C**, **F**, **I**) agglomerative clustering. Each panel for HDBSCAN and *k*-means clustering was generated with random number seeds, while agglomerative clustering is deterministic which is showed with three identical panels. HDBSCAN was unable to cluster Vemurafenib while both *k*-means and agglomerative clustering generated clusters of ~20 compounds.

© The Author(s)

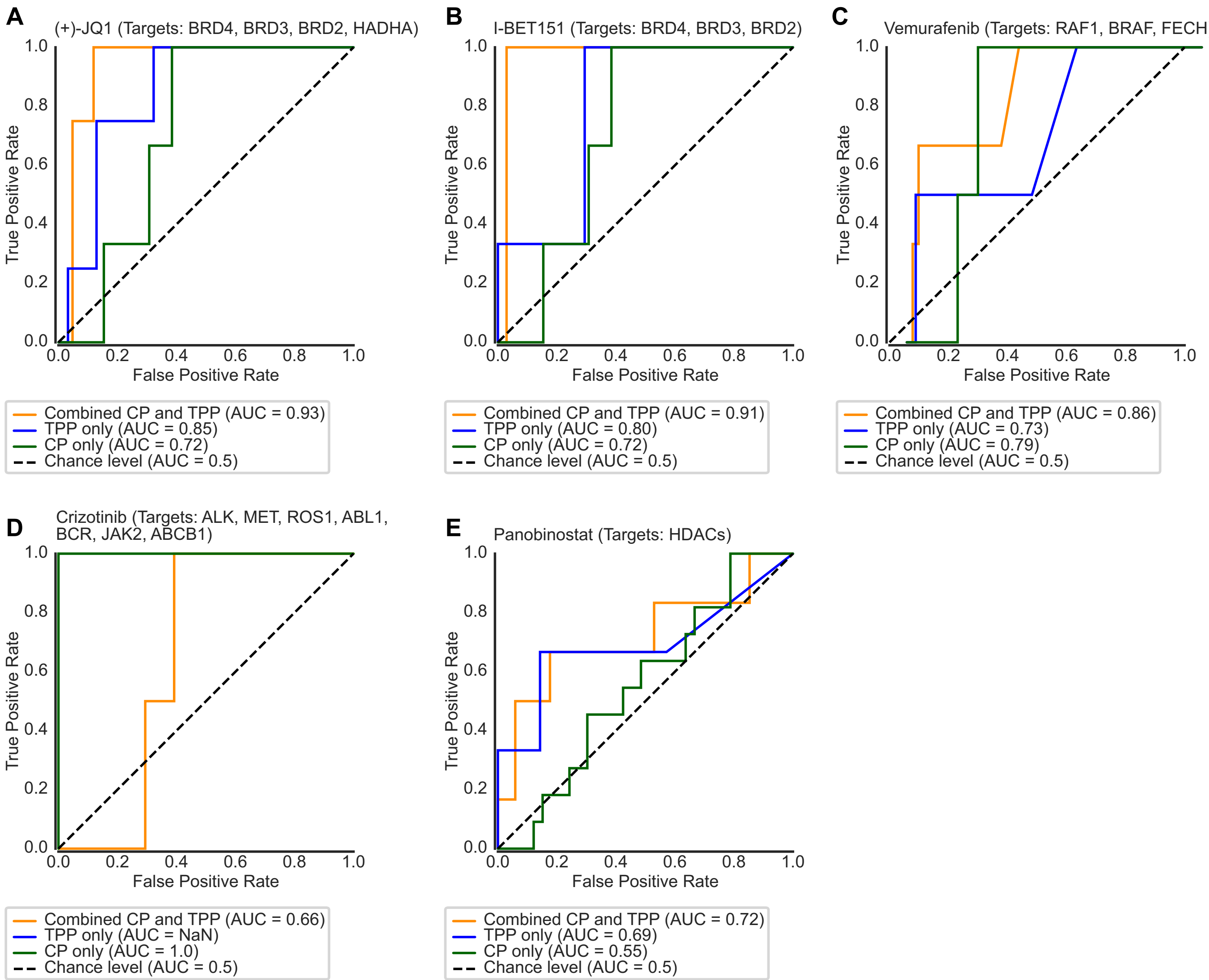

Fig. EV2. Combining cell painting (CP) and thermal protein profiling (TPP) generally improves target identification using betweenness centrality scores.

Receiver operating characteristic curves were constructed from PPI networks using betweenness centrality scores as ranks. (**A**) (+)-JQ1 (known targets: BRD4, BRD3, BRD2 and HADHA), (**B**) I-BET151 (known targets: BRD4, BRD3 and BRD2), (**C**) Vemurafenib (known targets: RAF1, BRAF and FECH), (**D**) Crizotinib (known targets: ALK, MET, ROS1, ABL1, BCR, JAK2 and ABCB1), (**E**) Panobinostat (known targets: HDACs).

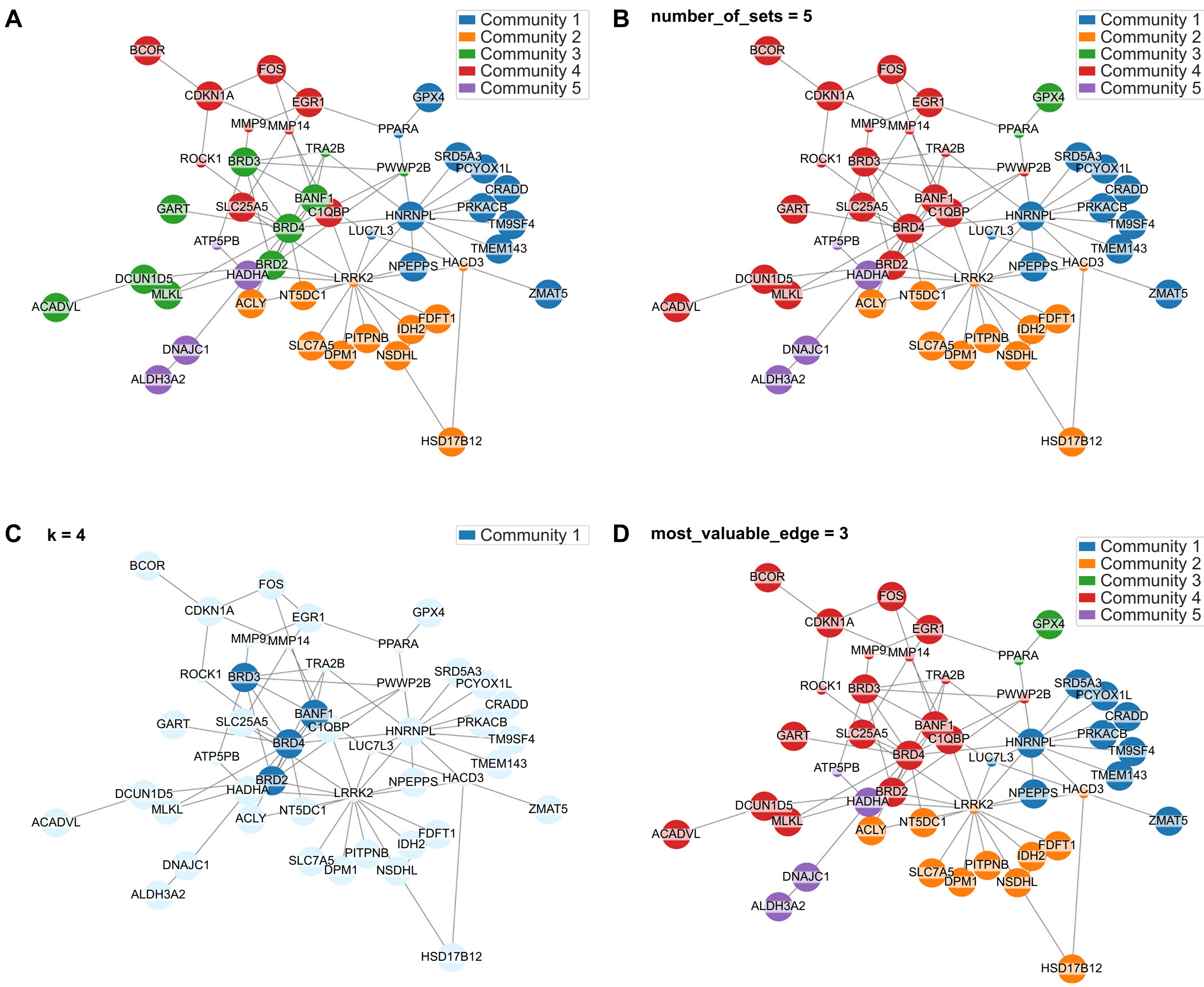

Fig. EV3. Evaluation of four different community detection algorithms on the final PPI networks of (+)-JQ1.

(**A**) Clauset-Newman-Moore greedy modularity maximization. (**B**) Girvan-Newman algorithm with number_of_sets set to 5. (**C**) *k*-clique communities algorithm with *k* set to 4. (**D**) Edge betweenness partition with most_valuable_edge set to 3. All four algorithms were executed using the NetworkX Python package.

 © The Author(s)
